# CCNE1 stabilizes ANLN by counteracting FZR1-mediated the ubiquitination modification to promotes triple negative breast cancer cell stemness and progression

**DOI:** 10.1038/s41420-025-02518-5

**Published:** 2025-05-09

**Authors:** Sujuan Dai, Lin Li, Guangxiu Guo, Yun Peng, Huozhong Yuan, Juntao Li

**Affiliations:** 1 Department of Pathology, The Affiliated Ganzhou Hospital of Nanchang University, Ganzhou, Jiangxi Province China; 2 Department of Pharmacy Intravenous Admixture Service, The Affiliated Ganzhou Hospital of Nanchang University, Ganzhou, Jiangxi Province China; 3 Department of Breast and Thyroid Surgery, The Affiliated Ganzhou Hospital of Nanchang University, Ganzhou, Jiangxi Province China

**Keywords:** Breast cancer, Cell biology, Molecular biology

## Abstract

Triple-negative breast cancer (TNBC) is an aggressive subtype lacking targeted therapies. In this study, we aimed to investigate the pivotal role of cyclin E1 (CCNE1) in the onset and progression of TNBC using comprehensive bioinformatic analysis and functional validation. We found significantly elevated CCNE1 expression in TNBC tissues compared to normal, which correlated with poor prognosis. Functional assessments in vitro and in vivo demonstrated that knockdown of CCNE1 impaired the proliferative, migratory, and invasive capacities of TNBC cells, promoted apoptosis, and reduced tumorigenicity. Furthermore, CCNE1 sustains the stem-like properties of TNBC cells and fuels malignant progression through Anillin (ANLN). Mechanistically, CCNE1 interacted with ANLN and stabilized its protein levels by counteracting Fizzy-related protein 1 (FZR1)-mediated the ubiquitination modification in TNBC. Mutation of the ubiquitination site in ANLN affected CCNE1’s regulatory functions but not ANLN’s intrinsic properties. Taken together, these findings underscore the role of CCNE1 in promoting TNBC cell stemness and progression via competitive inhibition of FZR1-mediated ANLN ubiquitination. Consequently, targeting CCNE1 emerges as a promising therapeutic approach for breast cancer.

## Introduction

Breast cancer is the most common malignancy among women and the leading cause of cancer-related death worldwide [1]. It is a heterogeneous disease characterized by different molecular profiles and clinicopathological features, and is classified into various subtypes based on receptor status, including receptor-positive (luminal A, luminal B, normal-like, and human epidermal growth factor receptor 2-positive, HER-2) and receptor-negative (triple-negative breast cancer, TNBC) subtypes [2]. TNBC, which lacks estrogen receptor (ER), progesterone receptor (PR), and human epidermal growth factor receptor 2 (HER2) expression, accounts for 10–20% of all breast cancers and is associated with a poor prognosis due to its aggressive nature and limited treatment options [3]. The high mortality and limited treatment options for TNBC highlight the urgent need to explore its molecular mechanisms and identify new therapeutic targets.

Cyclin E orchestrates cell cycle progression through precisely timed transcriptional activation and proteasomal degradation [4]. As the archetypal E-type cyclin, cyclin E1 (CCNE1; Gene ID:898) resides at chromosome 19q12, encoding a 410-amino acid regulatory protein that complexes with CDK2 to catalyze G1/S phase transition [5]. Mounting evidence positions CCNE1 overexpression as a dual oncogenic driver - inducing replication stress-mediated tumorigenesis while serving as a prognostic indicator across gynecological malignancies. This oncogenic signature manifests most prominently in endometrial intraepithelial neoplasia [6], ovarian clear cell carcinoma [7], and high-grade serous ovarian cancer [8], establishing CCNE1 as a pan-gynecological cancer biomarker.

In breast carcinogenesis, CCNE1 exhibits striking subtype-dependent pathobiology. Clinical cohort analyses consistently correlate its overexpression with aggressive clinicopathological features particularly in TNBC [9] and BLBC [10] subtypes-a phenomenon mechanistically linked to enhanced sensitivity to Wee1 kinase inhibitors in preclinical TNBC models [11]. The functional divergence between E-cyclin isoforms further enriches this molecular landscape: whereas CCNE2 governs NPAT-mediated transcriptional regulation in normal mammary epithelium [12], CCNE1 emerges as the predominant effector of cell cycle dysregulation during malignant progression. The therapeutic implications of these discoveries are further underscored by emerging evidence in hormone receptor-positive disease. As demonstrated by Scaltriti et al., differential expression patterns of E-cyclins in estrogen receptor-positive (ER+) breast cancers suggest their potential as predictive biomarkers and therapeutic targets [13]. These findings collectively highlight the context-dependent oncogenicity of CCNE1 across breast cancer subtypes, yet the precise molecular mechanisms underlying its subtype-specific pathogenicity remain incompletely characterized.

In this study, the significance of CCNE1 in TNBC from clinical, cellular and molecular levels was revealed. Our data demonstrated that CCNE1 knockdown inhibited the progression of TNBC by restraining proliferation, migration and tumor growth as well as enhancing apoptosis. These results suggested the role of CCNE1 as an effective molecular therapeutic target for TNBC.

## Result

### The correlation between CCNE1 expression and clinicopathological features in TNBC

**Fig. 1:**
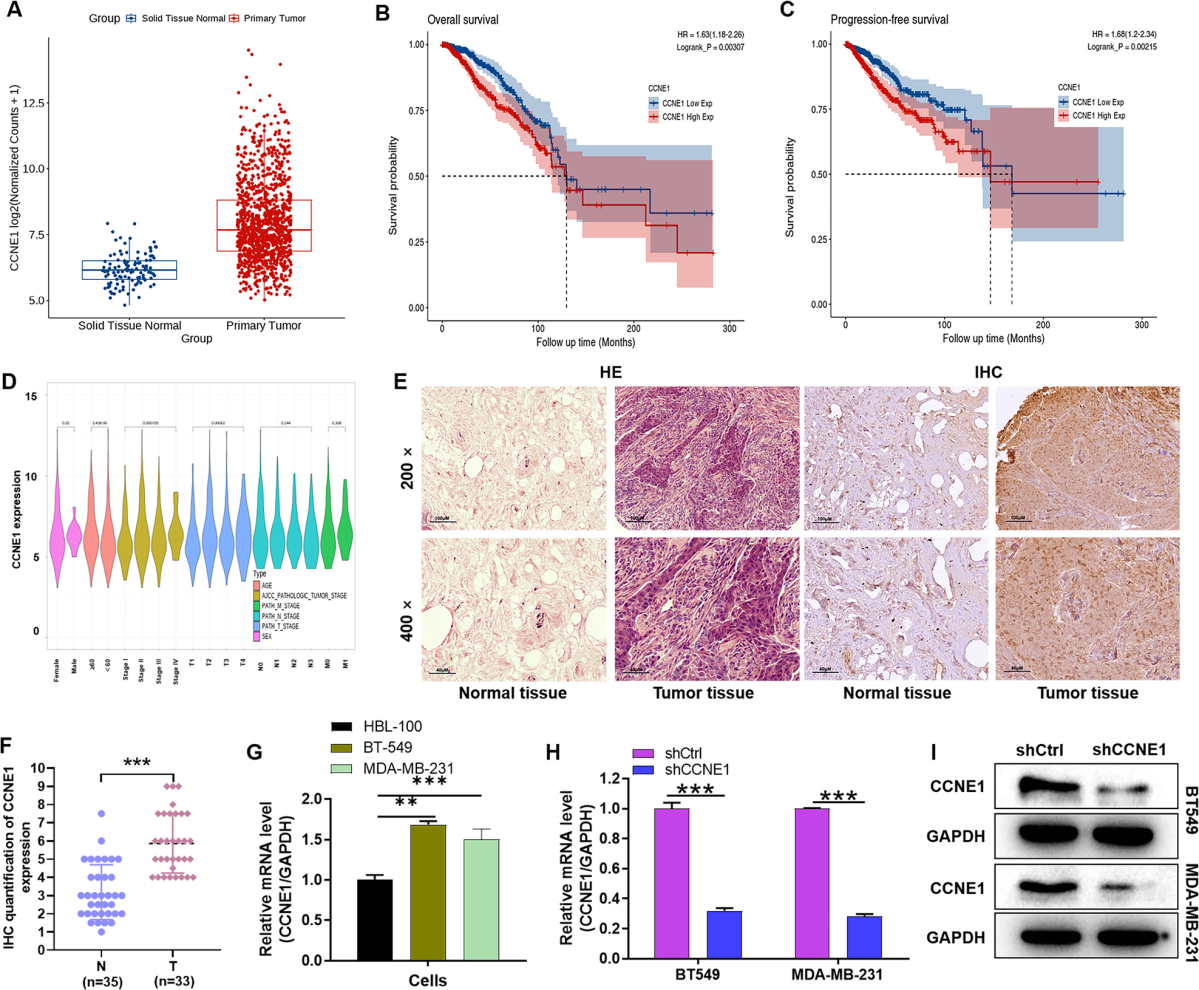
Expression of CCNE1 in TNBC and its correlation with prognosis. **A** The expression of CCNE1 in normal and tumor tissues was analyzed based on TCGA-TNBC information. **B**, **C** Kaplan–Meier analysis of the effect of CCNE1 expression on the overall survival and progression-free survival of TNBC patients from TCGA. **D** Cox multivariate regression analysis of the correlation between CCNE1 expression and clinical features of TNBC patients from TCGA. **E** Immunohistochemistry staining for quantification of CCNE1 expression in para-carcinoma tissues (*n* = 35) and tumor tissues (n = 33) of breast cancer. The “N” indicates normal tissues, “T” is for tumor tissues. **F** Typical images of hematoxylin & eosin (HE) staining and immunohistochemistry staining of para-carcinoma tissues and tumor tissues in TNBC patient. **G** The CCNE1 expression in TNBCcells BT-549 and MDA-MB-231 and normal breast cells HBL-100 was evaluated by qRT-PCR. (**H, I**) The knockdown efficiency of CCNE1 in TNBCcell lines on the mRNA and protein levels were evaluated by (**A**) qRT-PCR and (**B**) WB analysis, respectively. *n* = 3, **P < 0.01, ***P < 0.001.

To gain insights into the pivotal factors influencing the onset and progression of TNBC, a meticulous analysis was conducted utilizing the Cancer Genome Atlas (TCGA) database. Our findings unveiled a notable disparity in CCNE1 expression levels between breast cancer tissues and their normal counterparts (*P* < 0.001; Fig. 1A). To stratify the TNBC samples based on CCNE1 expression, the median value was adopted as the cutoff, thereby categorizing them into two distinct groups: high CCNE1 expression and low CCNE1 expression. Subsequently, the Kaplan–Meier analysis and log-rank test were employed to compare overall survival (Fig. 1B) and progression-free survival (Fig. 1C) between these groups. The results indicated that patients with high CCNE1 expression exhibited a markedly shorter overall survival duration compared to those with low expression. Cox multivariate regression analysis further substantiated the significant correlation between CCNE1 expression and prognosis, establishing CCNE1 as an independent prognostic indicator (Fig. 1D).

To visually confirm and elucidate the expression and distribution of CCNE1 in TNBC, hematoxylin and eosin (HE) staining and immunohistochemical (IHC) staining were conducted on tissue microarray chips comprising normal tissues (*n* = 35) and tumor tissues (*n* = 33) from TNBC patients. According to the IHC staining scores, samples with scores exceeding the median of 5 were classified as having high CCNE1 expression (positive staining), whereas those below the median were deemed negative. Notably, CCNE1 expression was predominantly observed in 51.5% (17 of 33) of the tumor tissues, in stark contrast to the mere 5.7% (2 of 35) positivity in corresponding normal tissues (*P* < 0.01; Fig. 1E, Table 1). As illustrated in Fig. 1F, CCNE1 staining was predominantly localized in the cytoplasm and nucleus-cytoplasm junction of tumor cells, with scarce positive signals detected in matched para-carcinoma tissues. Consistent with these observations, TNBC cell lines BT-549 and MDA-MB-231 exhibited significantly higher CCNE1 expression compared to the normal breast cell line HBL-100 (*P* < 0.01; Fig. 1G).

**Table 1:** Expression patterns in breast cancer tissues and para-carcinoma tissues were revealed by immunohistochemistry analysis.

CCNE1 expression	Tumor tissue		Para-carcinoma tissue		*P* value
	Cases	Percentage	Cases	Percentage	
Low	16	48.5%	33	94.3%	<0.001
High	17	51.5%	2	5.7%	

Furthermore, an examination of the relationship between CCNE1 expression and clinicopathological features in 33 breast cancer cases is presented in Table 2. The data revealed a statistically significant correlation between positive CCNE1 expression and tumor grade (*P* = 0.002) as well as T infiltrate (*P* = 0.035). However, no notable associations were found between CCNE1 expression and other clinicopathological characteristics, including age (*P* = 0.470), American Joint Committee on Cancer (AJCC) stage (*P* = 0.261), and lymphatic metastasis (*P* = 0.914). Spearman correlation analysis further corroborated the positive correlation between elevated CCNE1 expression and both T infiltrate (*P* = 0.033) and grade (*P* = 0.001) in TNBC patients (Table 3). Collectively, these data underscore the potential association between CCNE1 expression and poor prognosis in TNBC patients.

**Table 2:** Relationship between CCNE1 expression and tumor characteristics in patients with breast cancer.

Features	Patients number	CCNE1 expression		*P* value
		Low	High	
All patients	33	16	17	
Age (years)	33	16	17	0.470
Grade				0.002
II	7	7	0	
III	26	9	17	
AJCC stage				0.261
1	10	7	3	
2	20	7	13	
3	2	2	0	
4	1	0	1	
T Infiltrate				0.035
1	13	9	4	
2	18	7	11	
3	1	0	1	
4	1	0	1	
lymphatic metastasis (N)				0.914
0	22	11	11	
1	8	3	5	
2	1	1	0	
3	2	1	1

**Table 3:** Relationship between CCNE1 expression and tumor characteristics in patients with breast cancer.

		CCNE1
T Infiltrate	Spearman correlation coefficient	0.372
	Significance (two-tailed)	0.033
	N	33
Grade	Spearman correlation coefficient	0.535
	Significance (two-tailed)	0.001
	N	33

### Knockdown of CCNE1 inhibits TNBC cell growth and metastasis in vitro and in vivo

To investigate the biological function of CCNE1 in TNBC, lentiviral vectors (shCtrl or shCCNE1) were employed to specifically knockdown CCNE1 expression in BT-549 and MDA-MB-231 cell lines. The knockdown efficiency was confirmed at both mRNA and protein levels using qRT-PCR (Fig. 1H) and Western blot (Fig. 1I) analysis, indicating successful establishment of the CCNE1-knockdown TNBC cell model.

**Fig. 2:**
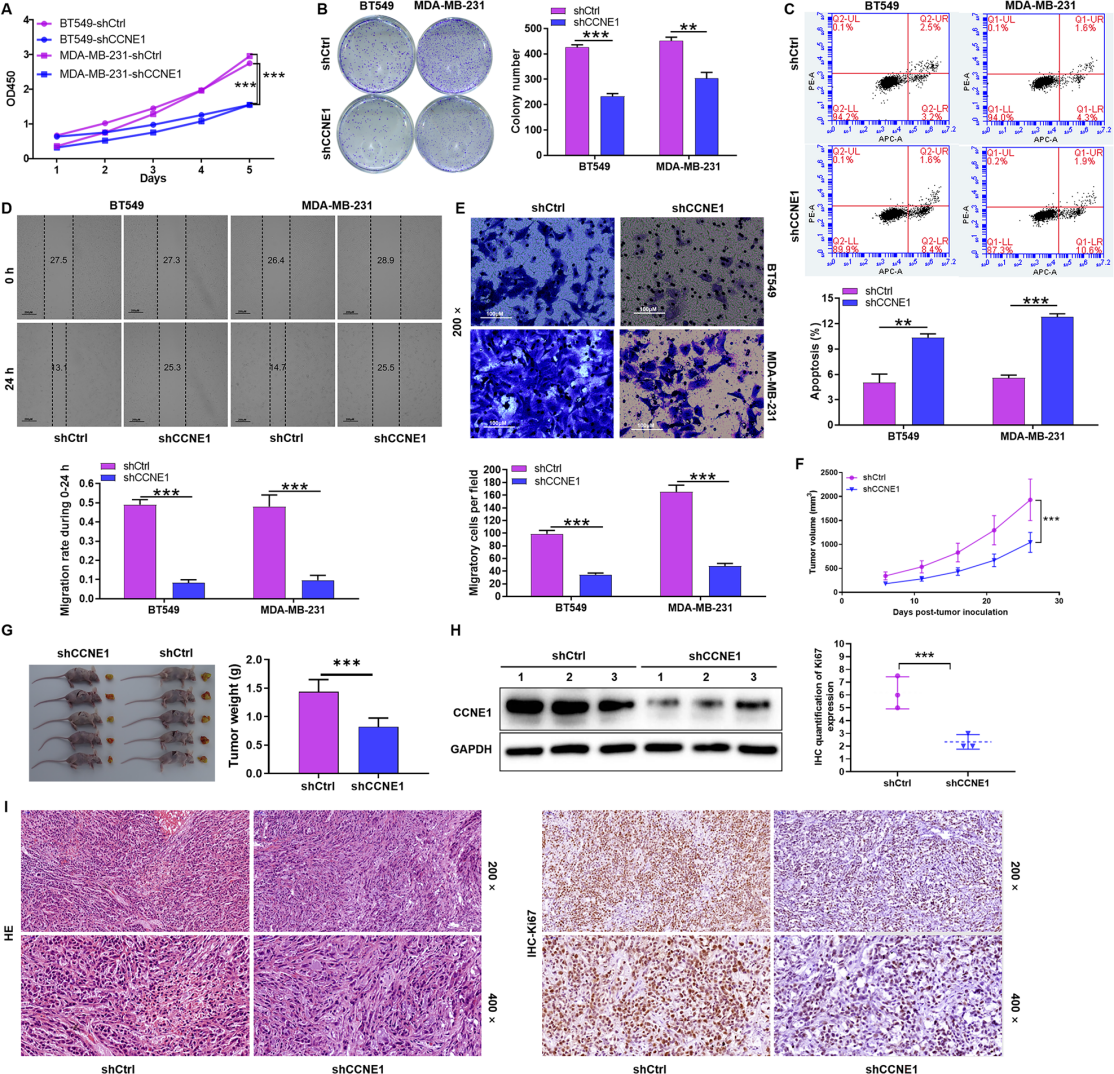
Knockdown of CCNE1 inhibits TNBC cell growth and metastasis in vitro and in vivo. The proliferative ability of BT-549 and MDA-MB-231 cells transfected with lentivirus shCtrl or shCCNE1 was evaluated by (**A**) MTT assay and (**B**) cell clonal formation assay, respectively. **C** Cell apoptosis of BT-549 and MDA-MB-231 following CCNE1 knockdown was measured by flow cytometry. **D** The migration of BT-549 and MDA-MB-231 cells transfected with lentivirus shCtrl or shCCNE1 was evaluated by wound-healing assay. **E** The invasion of BT-549 and MDA-MB-231 cells transfected with lentivirus shCtrl or shCCNE1 was evaluated by Transwell assay. *n* = 3, **P < 0.01, ***P < 0.001. MDA-MB-231 cells into the right forelimb axillary of nude mice. **F** On day 26, the mice were placed in the In Vivo Imaging System for total fluorescence intensity of mice tumor. **G** After tumor formation, body weight of mice was recorded, and tumor dimensions were measured twice weekly using digital calipers during the experimental period, respectively. **H** After the mice tumor tissue protein was extracted and performed WB analysis to detected the expression of CCNE1 in shCCNE1 groups and shCtrl groups. **I** Typical images of HE staining and immunohistochemistry staining of mice tumor tissue in shCCNE1 groups and shCtrl groups. *n* = 5, *P < 0.05, ***P < 0.001.

Functional assessments revealed that CCNE1 knockdown impaired the proliferative capacity of TNBC cells. Specifically, MTT assays (Fig. 2A) and cell clonal formation (Fig. 2B) demonstrated decreased proliferation rates and colony formation in both BT-549 and MDA-MB-231 cells transfected with shCCNE1 compared to shCtrl. Flow cytometry analysis further showed increased apoptosis in shCCNE1-transfected BT-549 (from 3.2% to 8.4%) and MDA-MB-231 cells (from 4.3% to 10.6%) (Fig. 2C). Moreover, CCNE1 knockdown suppressed TNBC cell migration and invasion. Wound-healing and Transwell assays indicated reduced migration rates by approximately 80% (Fig. 2D) and impaired invasion capacity by approximately 64.9% and 70.9% in BT-549 and MDA-MB-231 cells, respectively (Fig. 2E).

To validate the oncogenic role of CCNE1 in vivo, xenograft tumor mouse models were established by subcutaneous injection of shCtrl or shCCNE1 MDA-MB-231 cells. On day 26, In Vivo Imaging System analysis revealed significantly weaker total fluorescence intensity in the shCCNE1 group compared to the shCtrl group (Fig. 2F). Consistently, tumor volumes and weights were significantly decreased in mice injected with CCNE1-knockdown cells (Fig. 2G). Western blot analysis of tumor tissue protein extracts confirmed downregulation of CCNE1 expression in the shCCNE1 groups (Fig. 2H). Histological examination and immunohistochemistry staining of the nodules identified them as tumors and demonstrated reduced KI67 expression in the shCCNE1 group compared to the shCtrl group (Fig. 2I). Collectively, these findings demonstrate that CCNE1 knockdown inhibits tumorigenicity in vivo, further underscoring its critical role in TNBC progression.

### CCNE1 sustains the stem-like properties of TNBC cells and fuels malignant progression through ANLN

To elucidate the regulatory role of CCNE1 in TNBC cells, we utilized the STRING database to predict specific interacting molecules with CCNE1. Our analysis indicated Anillin (ANLN) as the protein with the highest interaction score with CCNE1 (Supplementary Table S2). Data from the TCGA database further indicated that ANLN is highly expressed in breast cancer and significantly correlates with overall and progression-free survival (Supplementary Fig. S1A–C).

**Fig. 3:**
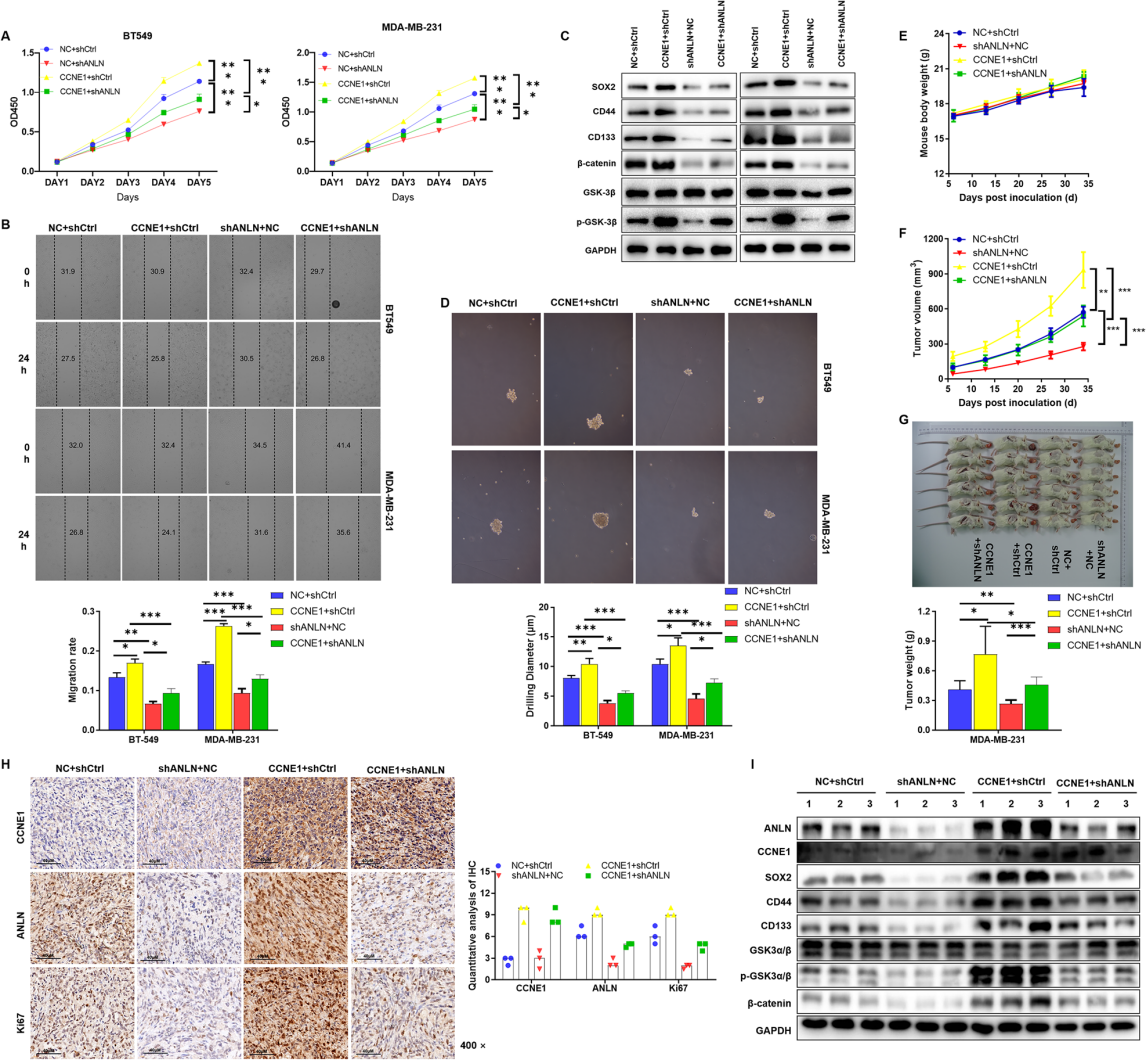
CCNE1 sustains the stem-like properties of TNBC cells and fuels malignant progression through ANLN. TNBCcells BT-549 and MDA-MB-231 with CCNE1 overexpression and ANLN knockdown were constructed. **A** The proliferative ability of TNBCcells was evaluated by MTT assay. **B** The migration of TNBCcells was tested by wound-healing assay. **C** WB assay was used to detect stemness markers protein expression in BT-549 and MDA-MB-231 cells. **D** The sphere-forming ability was detected in BT-549 and MDA-MB-231 cells. *n* = 3, *P < 0.05, **P < 0.01, ***P < 0.001. **E–G** MDA-MB-231 cells with overexpression of CCNE1 and knockdown of ANLN were injected subcutaneously into mice to establish xenograft tumor model, and the growth and tumor size of mice were observed. **H** Typical images of immunohistochemistry staining of mice tumor tissue in overexpression of CCNE1 and knockdown of ANLN. **I** The protein expression levels of CCNE1, ANLN, and stemness markers in the tumor tissues was evaluated by WB analysis. *n* = 6, *P < 0.05, ***P < 0.001.

To investigate this interaction, we established TNBC cell models with ANLN knockdown (Supplementary Fig. S1D, E) and CCNE1 overexpression (Supplementary Fig. S1F, G), and conducted functional recovery experiments in vitro. Our results demonstrated that ANLN knockdown partially reversed the proliferative (Fig. 3A) and migratory (Fig. 3B) enhancements induced by CCNE1 overexpression in BT-549 and MDA-MB-231 cells.

Previous studies have reported ANLN’s role in enhancing TNBC stemness [14]. Consistently, our findings showed that ANLN knockdown inhibited TNBC stem cell properties, as evidenced by reduced expression of stemness markers (Fig. 3C) and sphere-forming ability (Fig. 3D). Notably, in the presence of CCNE1, the expression of stemness markers and sphere-forming capacity were augmented, while ANLN knockdown mitigated this regulation by CCNE1 (Fig. 3C, D). These results suggest that CCNE1 influences TNBC progression by regulating stemness properties through ANLN.

Furthermore, we established xenograft tumor mouse models by subcutaneous injection of MDA-MB-231 cells with CCNE1 overexpression and ANLN knockdown. In vivo data revealed that ANLN knockdown partially reversed the tumor growth-promoting effect of CCNE1 overexpression (Fig. 3E–G). Histological examination and immunohistochemical staining confirmed the tumorigenic nature of the nodules, with expression of CCNE1, ANLN, and Ki67 (Fig. 3H). Consistent with our in vitro findings, protein expression levels of CCNE1, ANLN, and stemness markers in tumors across all groups further supported the notion that CCNE1 regulates TNBC progression by influencing stemness properties through ANLN (Fig. 3I). Taken together, these findings demonstrate that CCNE1 sustains the stem-like properties of TNBC cells and fuels malignant progression through ANLN.

### CCNE1 stabilizes ANLN by counteracting FZR1-mediated the ubiquitination modification in TNBC

**Fig. 4:**
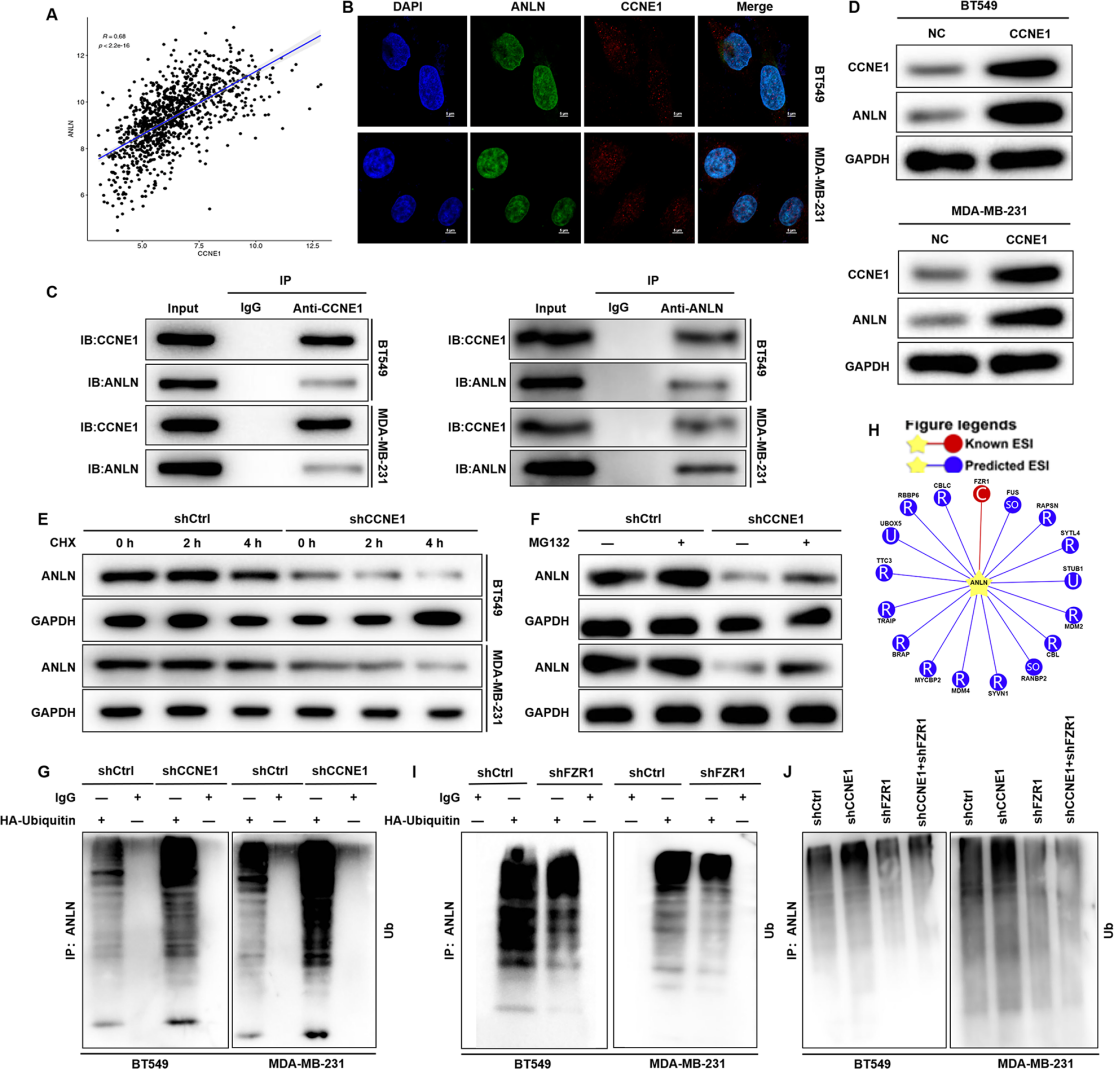
Molecular mechanism of CCNE1 stabilization of ANLN protein expression. **A** Pearson correlation analysis from TCGA demonstrated a significant positive correlation between CCNE1 and ANLN expression levels. **B** Triple immunofluorescence staining of CCNE1 (red), ANLN (green), and the cell nucleus (DAPI, blue) demonstrated that CCNE1 and ANLN primarily colocalize within the nuclei of TNBC cells. **C** Endogenous co-immunoprecipitation assay from TNBCcells revealed protein interactions between CCNE1 and ANLN. **D** Western blot experiment conducted on TNBC cells demonstrated that CCNE1 has the ability to enhance the expression of ANLN protein. **E** The stability of ANLN protein was tested after treatment of CCNE1-knockdown BT-549 and MDA-MB-231 cells with the 40 μM protein synthesis inhibitor cycloheximide (CHX). **F** Upon treatment with the proteasome inhibitor 10 μM MG132, ANLN protein stability was tested in CCNE1-knockdown BT-549 and MDA-MB-231 cells. **G** Ubiquitination assay of ANLN in CCNE1-knockdown BT-549 and MDA-MB-231 cells treated for 4 h with 10 μM MG132. **H** The E3 ligases that can regulate ANLN substrates were searched based on UbiBrowser2 database. **I**, **J** Ubiquitination assay of ANLN in (**H**) FZR1-knockdown or (**I**) CCNE1/FZR1 knock down coexisting BT-549 and MDA-MB-231 cells treated for 4 h with 10 μM MG132.

Our analysis of TNBC samples in the TCGA database revealed a significant positive correlation between CCNE1 and ANLN expression levels using Pearson correlation analysis (Fig. 4A). Triple immunofluorescence staining of CCNE1 (red), ANLN (green), and the cell nucleus (DAPI, blue) demonstrated that CCNE1 and ANLN primarily colocalize within the nuclei of TNBC cells (Fig. 4B). Immunoprecipitation using a CCNE1 antibody successfully precipitated not only the CCNE1 protein but also the ANLN protein, confirming an interaction between CCNE1 and ANLN, and vice versa (Fig. 4C). Furthermore, overexpression of CCNE1 upregulated ANLN protein expression (Fig. 4D) without affecting its mRNA expression (Supplementary Figure S2A), suggesting that CCNE1 may regulate ANLN expression through post-transcriptional modifications.

With increasing treatment duration of the protein synthesis inhibitor cycloheximide (CHX), TNBC cells (BT-549 and MDA-MB-231) with CCNE1 knockdown exhibited a greater decrease in ANLN protein levels compared to control cells (Fig. 4D). This indicated that ANLN protein half-life was reduced following CCNE1 knockdown, suggesting that CCNE1 depletion destabilizes ANLN. Intriguingly, treatment with the proteasome inhibitor MG132 partially alleviated the effect of CCNE1 knockdown on ANLN protein stability (Fig. 4F). These findings suggest that CCNE1 may regulate ANLN protein expression through the proteasome pathway.

Subsequent immunoblotting using a ubiquitin antibody showed a marked increase in ANLN ubiquitination upon downregulation of CCNE1 (Fig. 4G). A search in the UbiBrowser2 database identified Fizzy-related protein 1 (FZR1) as a potential E3 ubiquitin ligase regulating the substrate protein ANLN (Fig. 4H), and FZR1 expression was negatively correlated with ANLN expression (Supplementary Fig. S2B). As shown in Fig. 4I, knockdown of FZR1 resulted in a significant reduction in CCNE1 ubiquitination, indicating that FZR1 regulates CCNE1 ubiquitination. Simultaneous knockdown of CCNE1 in the context of FZR1 knockdown effectively reversed FZR1’s regulation of CCNE1 ubiquitination (Fig. 4J), suggesting that CCNE1 and FZR1 compete for ubiquitination of the substrate protein ANLN. Consistently, analysis of TNBC samples in the TCGA database showed a negative correlation between CCNE1 and FZR1 expression (Supplementary Fig. S2C). Collectively, these results demonstrate that high expression of CCNE1 in TNBC stabilizes ANLN protein levels by preventing FZR1-mediated ubiquitination of ANLN.

In addition, given the high structural and functional similarity between CCNE1 and CCNE2, both genes are essential for tumor cell proliferation. This study revealed that CCNE2 expression was significantly lower in TNBC cell lines compared to CCNE1 (Supplementary Fig. S2D). Furthermore, CCNE1 and CCNE2 generally influence tumor development and progression through the regulation of RB phosphorylation. However, our findings indicate that while CCNE1 knockdown reduced RB phosphorylation, neither CCNE2 knockdown nor overexpression altered RB phosphorylation levels (Supplementary Fig. S2E), suggesting a lack of compensatory mechanisms involving CCNE2 in TNBC.

### CCNE1 exerts regulatory functions in TNBC through the ubiquitination modification of ANLN

**Fig. 5:**
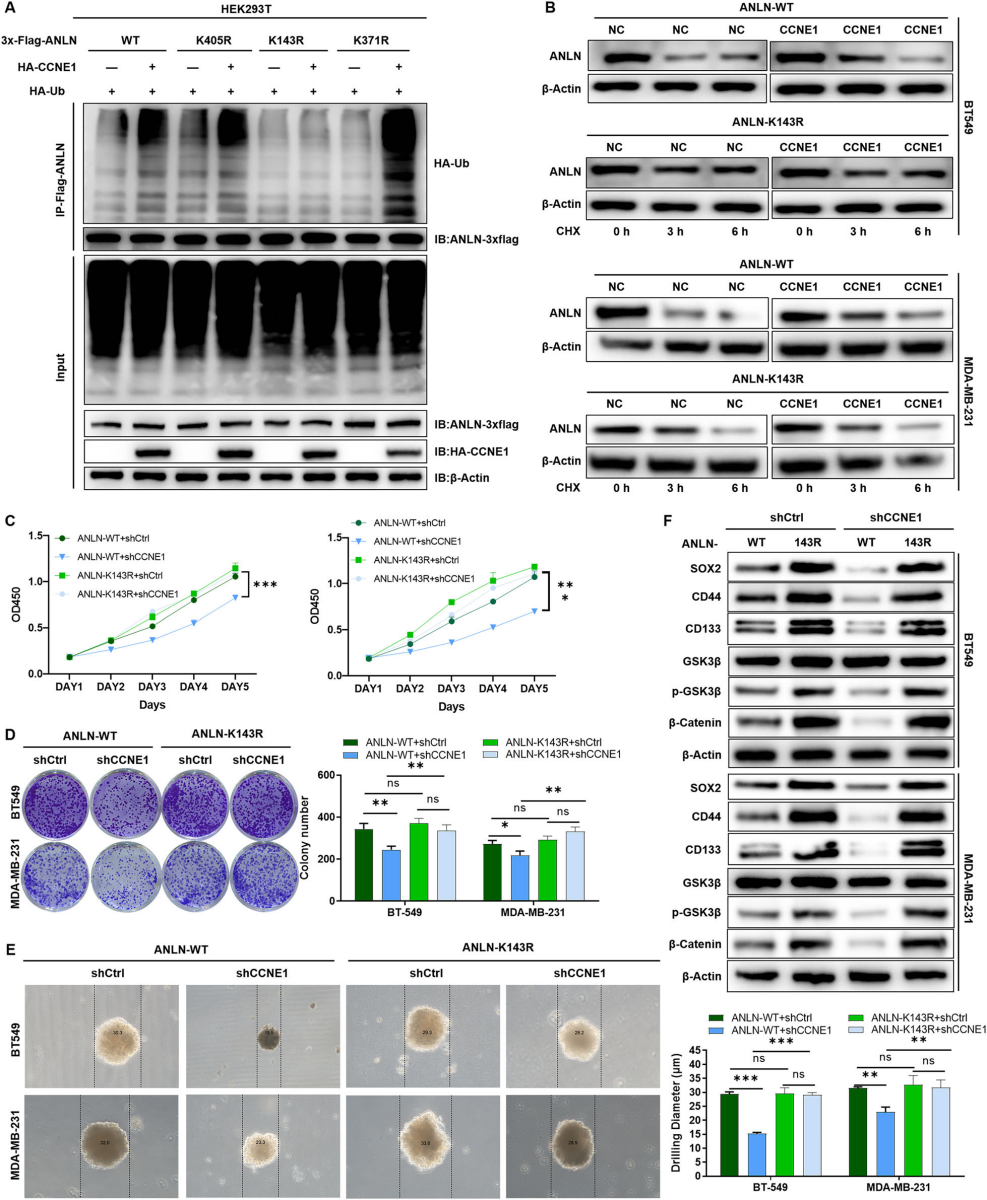
CCNE1 exerts regulatory functions in TNBC through the ubiquitination modification of ANLN. **A** The E3-specific ubiquitination sites of the substrate protein ANLN, specifically lysine residues 143, 371, and 405, were mutated to arginine. Flag-tagged plasmids K143R, K371R, and K405R were subsequently constructed, followed by the execution of ubiquitination detection experiments. **B** After addition of the 40 μM CHX, the stability of ANLN protein was tested in BT-549 and MDA-MB-231 cells with overexpressing either wild-type ANLN (ANLN-WT) or the ubiquitination site-mutated ANLN (ANLN-K143R). The proliferative ability of TNBC cells overexpressing either wild-type ANLN (ANLN-WT) or the ubiquitination site-mutated ANLN (ANLN-K143R) was evaluated by (**C**) MTT assay and (**D**) clonal formation assay, respectively. Plasmid overexpressing wild-type ANLN (ANLN-WT) or ANLN with ubiquitination site mutation (ANLN-K143R) was transfected into CCNE1 knockdown and control TNBC cells, and (**E**) the ball forming ability and (**F**) expression of stem cell markers were detected. *n* = 3, *P < 0.05, **P < 0.01, ***P < 0.001.

To investigate the molecular mechanisms and regulatory roles of ubiquitination in ANLN, we employed the GPS-Uber (GPS-Ubiquitin-protein ligase Enzymes-substrate relationship prediction) database to predict the E3-specific ubiquitination sites within the substrate protein ANLN, identifying lysine residues 143, 371, and 405 as potential targets. Subsequently, these lysine residues were mutated to arginine individually, and distinct plasmids tagged with Flag were constructed, namely K143R, K371R, and K405R. As illustrated in Fig. 5A, overexpression of CCNE1 significantly altered the ubiquitination levels of the corresponding ANLN variants, with the ubiquitination modification of ANLN being unaffected by CCNE1 expression in the presence of the K143 lysine mutant. Collectively, these findings indicate that CCNE1-mediated ubiquitination of the substrate protein ANLN occurs specifically at lysine 143. Furthermore, CCNE1 solely facilitated the degradation of wild-type ANLN protein, losing this pro-degradative capability upon mutation of lysine 143, suggesting that the K143 ubiquitination site is crucial for ANLN protein degradation (Fig. 5B).

To explore the functional impact of mutating the ubiquitination site K143 on CCNE1 regulation in TNBC, plasmids overexpressing either wild-type ANLN (ANLN-WT) or the ubiquitination site-mutated ANLN (ANLN-K143R) were transfected into TNBC cells with CCNE1 knockdown and corresponding controls, followed by appropriate assays. As shown in Fig. 5C, D, there were virtually no differences in cell proliferation and clonogenic formation abilities between ANLN-WT and ANLN-K143R, indicating that mutation of the ubiquitination site K143 does not affect the intrinsic function of CCNE1 in regulating cellular phenotypes. Notably, the inhibition of TNBC cell proliferation and clonogenic formation upon CCNE1 knockdown was more pronounced in the ANLN-WT background compared to the ANLN-K143R background. Consistently, similar regulatory trends were observed in TNBC cell spheroid formation capabilities and the expression of stemness-related markers under these conditions (Fig. 5E, F). In summary, these results demonstrate that mutation of the ubiquitination site in ANLN does not alter the properties of ANLN itself but impacts the functionality of CCNE1, revealing that “CCNE1-mediated ubiquitination of ANLN” is pivotal for CCNE1 to exert its regulatory functions in TNBC.

## Discussion

Our study elucidates the multifaceted oncogenic role of CCNE1 in TNBC progression through mechanistically distinct pathways, advancing current understanding of cyclin-driven carcinogenesis in three critical dimensions. First, we establish CCNE1 as a subtype-specific prognostic determinant in TNBC, contrasting with prior studies that primarily focused on its pan-cancer roles [15]. While earlier work identified CCNE1 overexpression as a poor prognostic factor in ER+ breast cancer [13], our TCGA analysis and tissue validation specifically implicate its clinical relevance in TNB -a particularly aggressive subtype lacking targeted therapies. This subtype specificity resolves the paradox observed in genome-wide association studies where CCNE1 amplification showed variable prognostic significance across breast cancer subtypes [10, 16, 17].

Our confirmation of CCNE1’s prognostic value in TNBC corroborates preclinical observations by Ha et al. [9, 18], but their focus on Wee1 inhibitor sensitivity differs from our mechanistic exploration of stemness regulation. Our discovery of the CCNE1-Anilin actin-binding protein (ANLN) regulatory axis introduces a novel mechanism underlying TNBC stemness maintenance. Previous investigations positioned TWIST1 and BMP2 as essential genes that mediate ANLN’s function in TNBC stemness [14], but our functional rescue experiments reveal its unexpected role as a downstream effector of CCNE1-mediated stem cell maintenance. This finding contrasts with the canonical view of cyclins as purely cell cycle regulators, expanding their functional repertoire to include direct modulation of stemness pathways. Notably, our identification of K143 as the critical ubiquitination site on ANLIN provides structural specificity to this interaction, addressing a key knowledge gap in post-translational regulation of stemness factors.

Third, the elucidation of the CCNE1-Fizzy-related protein 1 (FZR1) competitive ubiquitination mechanism represents a paradigm shift in understanding cyclin E’s non-canonical functions. While previous studies implicated FZR1 in cell cycle [19, 20], our competitive ubiquitination model introduces spatial-temporal regulation of substrate stabilization during tumor progression. Additionally, while CCNE1-CDK2 complexes are known to phosphorylate substrates like RB [21], our demonstration of CCNE1’s E3 ligase-antagonizing activity through FZR1 competition reveals an entirely new regulatory layer. This finding aligns with emerging evidence of cyclin moonlighting functions [22] but extends the concept to ubiquitination pathway modulation. Importantly, the absence of compensatory CCNE2 effects in our system (Supplementary Fig. S2D, E) challenges the prevailing assumption of E-cyclin functional redundancy, suggesting evolutionary divergence in their oncogenic mechanisms.

In conclusion, the clinical implications of our study offer promising avenues for the management of TNBC. Specifically, the CCNE1 high/ANLN high signature emerges as a potential stratify for TNBC patients eligible for targeted therapy, addressing a critical unmet need in this aggressive subtype. Furthermore, the identification of the K143 ubiquitination site presents a novel druggable interface that could be targeted by small-molecule inhibitors, thereby expanding the therapeutic arsenal against TNBC. Additionally, the expression levels of FZR1 may serve as a predictive biomarker for response to CCNE1-targeted therapies, enhancing the precision of treatment strategies. However, several study limitations must be acknowledged. Firstly, our xenograft models, while informative, cannot fully replicate the complex tumor microenvironment of TNBC, limiting the generalizability of our findings. Secondly, the relatively small size of the clinical cohort (*n* = 33) necessitates validation in larger, multi-center cohorts to consolidate the robustness of our observations. Lastly, although FZR1 is established as the primary E3 ligase in this context, the potential for cooperativity with other ubiquitination enzymes remains an unexplored area.

In light of these considerations, several future directions are envisioned to build upon our current findings. Firstly, developing K143-specific ANLN mutants for in vivo validation will be crucial to substantiate the role of this ubiquitination site in TNBC progression. Secondly, screening for compounds that disrupt the CCNE1-ANLN interaction could lead to the discovery of novel therapeutic agents. Thirdly, investigating the microenvironmental factors that modulate this axis will provide deeper insights into the biological mechanisms underlying TNBC resistance and progression. Ultimately, the development of CCNE1/ANLN dual-targeting strategies could synergize with existing CDK4/6 inhibitors, offering a potential pathway to overcome therapeutic resistance in TNBC, thereby advancing the field towards more effective and personalized treatment approaches.

## Materials and methods

### Ethical statement

All experimental procedures were conducted in accordance with the Declaration of Helsinki and approved by the Institutional Review Board of The Affiliated Ganzhou Hospital of Nanchang University. Written informed consent was obtained from all participants prior to tissue collection.

### Immunohistochemistry (IHC) analysis

A tissue microarray (TMA) containing 33 TNBC specimens and 35 matched normal tissues (5 μm thickness) was obtained from The Affiliated Ganzhou Hospital of Nanchang University. TMA sections were dewaxed in xylene (Sigma-Aldrich, USA, #214736) thrice (15 min each) and rehydrated through graded ethanol series (100–70%). Heat-mediated epitope retrieval was performed in citrate buffer (10 mM, pH 6.0; Abcam, UK, #ab93678) using a pressure cooker (121 °C, 15 min). Endogenous peroxidase activity was quenched with 3% H2O2 (Beyotime, China, #ST2069) in PBS-Tween 20 (0.1% v/v; Solarbio, China, #T8220) for 20 min at room temperature (RT). Sections were incubated with rabbit anti-CCNE1 monoclonal antibody (Abcam, UK, #ab33912; 1:50 dilution in PBS/1% BSA) overnight at 4 °C in a humidified chamber. After PBS washes, sections were treated with HRP-conjugated goat anti-rabbit IgG (Abcam, UK, #ab6721; 1:400) for 2 h at RT. Diaminobenzidine (DAB) substrate (Dako, Denmark, #K3468) was applied for 10 min under microscopic monitoring. Hematoxylin (Sigma-Aldrich, #HHS32) staining (5 min) preceded dehydration through ethanol-xylene series and permanent mounting with neutral balsam (Solarbio, #G8590). CCNE1 expression was evaluated using a semi-quantitative H-score system [23] incorporating staining intensity (0–3: negative/weak/moderate/strong) and percentage of positive cells. Final scores (range 0–300) were calculated as: H-score= (∑Intensity Percentage of cells). Samples were stratified into high (≥ median score) and low (<median) expression groups for statistical analysis.

### Cell culture

BT-549 (RRID: CVCL_1092) and MDA-MB-231 (RRID: CVCL_0062) TNBC cell lines, along with HBL-100 normal breast cells, were obtained from the American Type Culture Collection (ATCC, USA). These cells were cultured in a humidified incubator (Thermo Fisher Scientific, USA) at 37 °C with 5% CO₂, authenticated by STR profiling and tested for mycoplasma Contamination. The culture medium used was Dulbecco’s Modified Eagle Medium (DMEM, Sigma-Aldrich, USA) supplemented with 10% fetal bovine serum (FBS, Gibco, USA), 100 IU/mL penicillin, and 100 µg/mL streptomycin.

### RNA extraction and quantitative real-time PCR (qRT-PCR)

Cellular RNA was extracted using TRIzol reagent (Invitrogen, USA) according to the manufacturer’s instructions. RNA concentration was measured using a NanoDrop 100 spectrophotometer (Thermo Fisher Scientific, USA). Reverse transcription was performed using the HiScript QRT SuperMix Kit (Vazyme, China) following the manufacturer’s protocol. Quantitative real-time PCR (qRT-PCR) was conducted using the SYBR Green Master Mix Kit (Vazyme, China) on the Applied Biosystems 7500 Sequence Detection System. The mRNA levels were quantified using the 2⁻ΔΔCt method, normalized to GAPDH. The primer sequences used were as follows: CCNE1-forward: 5′-CTGGATGTTGACTGCCTTGAA-3′ and reverse: 5′-CGCACCACTGATACCCTGAAA-3′; GAPDH-forward: 5′-TGACTTCAACAGCGACACCCA-3′ and reverse: 5′-CACCCTGTTGCTGTAGCCAAA-3′.

### Plasmid construction, and cell transfection

Negative control scramble sequences (shCtrl: 5′-TTCTCCGAACGTGTCACGT-3′) and short hairpin RNA (shRNA) targeting CCNE1 (shCCNE1-1: 5′-CTGGACAAAGCCCGAGCAAAG-3′, shCCNE1-2: 5′-CTGGGCAAATAGAGAGGAAGT-3′, shCCNE1-3: 5′-CAGGGTATCAGTGGTGCGACA-3′) were designed and synthesized. These sequences were then cloned into lentiviral vectors LV-002 (YBR, China) at the EcoR I and Age I cleavage sites using T4 DNA ligase. Recombinant lentivirus containing the target sequences (shCtrl and shCCNE1) was transfected into BT-549 and MDA-MB-231 cells using Lipofectamine 3000 (Invitrogen, USA) in DMEM culture medium with 10% FBS at a multiplicity of infection (MOI) of 10 for 30 min. The cells were incubated at 37 °C for 72 h, and stable cell lines were selected using puromycin at a concentration of 5 μg/mL.

### Protein extraction and western blotting (WB)

Total cellular protein was extracted using cell lysis buffer (Promega, USA) and quantified using the BCA Protein Assay Kit (Pierce, USA). Equal amounts of protein were separated by 10% SDS-PAGE and transferred to a PVDF membrane (Millipore Corporation, USA). The membrane was blocked with TBST solution containing 5% skimmed milk at RT for 1 h. After washing with PBS, the membrane was incubated with primary antibodies (Supplementary Table S1) overnight at 4 °C on a rocker, followed by incubation with HRP-coupled secondary antibodies (Supplementary Table S1) for 1 h at RT. Immunoreactive proteins were visualized using the enhanced chemiluminescence ECL+PlusTM detection system (Amersham Pharmacia Biotech, USA).

### MTT assay

BT-549 and MDA-MB-231 cells were plated in 96-well plates at a density of 5 × 10³ cells/well. After 1, 2, 3, 4, or 5 days of incubation, 20 µL of MTT solution (Genview) was added to each well and incubated for 4 h. Subsequently, the cells were treated with 100 µL of dimethyl sulfoxide (DMSO), shaken for 5 min at RT, and the optical density (OD) of each well was measured at 450 nm using a microplate reader (Tecan Infinite M200 PRO, Switzerland).

### Colony formation assay

BT-549 and MDA-MB-231 cells were cultured in DMEM (Sigma-Aldrich, USA) supplemented with 10% fetal bovine serum (FBS, Gibco, USA), 100 IU/mL penicillin, and 100 µg/mL streptomycin. For the colony formation assay, cells were trypsinized, resuspended, and inoculated in six-well plates at 500 cells/well in triplicate. They were maintained in DMEM containing 10% FBS at 37 °C with 5% CO₂. After 14 days, cells were fixed with 4% paraformaldehyde for 30 min, stained with Giemsa for 20 min, and photographed. The number of colonies containing more than 50 cells per well was evaluated using an inverted light microscope (Olympus, Japan).

### Detection of cell apoptosis by flow cytometry

Cell apoptosis was measured using the Annexin V apoptosis kit (eBioscience, USA) according to the manufacturer’s instructions. Briefly, BT-549 and MDA-MB-231 cells were washed with 1× binding buffer, resuspended in 200 µL of binding buffer, and incubated with 10 µL of Annexin V-APC at RT for 15 min in the dark. The apoptotic rate was then assessed using a flow cytometer (Millipore, USA).

### Wound-healing assay

BT-549 and MDA-MB-231 cells were digested with pancreatic enzymes, resuspended in DMEM with 10% FBS, and plated at 5 × 10⁴ cells/well in 96-well plates. On the second day, cells were starved with serum-free DMEM medium and a scratch was made in the center of each well using a wounding replicator (VP Scientific, USA). The initial state was photographed and recorded as 0 h. Subsequently, images were obtained 24 h later using a Cellomics ArrayScan VTI analyzer (Thermo Fisher Scientific, USA), and the migration ability was calculated using the formula: migration area = 24 h cell area − 0 h cell area.

### Transwell invasion assay

The invasive potential of BT-549 and MDA-MB-231 cells was evaluated using Transwell plates with an 8.0 µm pore size (Corning, USA). Cells were starved in serum-free DMEM medium and inoculated into the upper chamber coated with Matrigel. The lower chamber contained 600 µL of medium with 30% fetal bovine serum. After 24 h of incubation at 37 °C, cells on the upper surface of the membrane were removed with cotton swabs, while cells on the lower surface were fixed with 4% paraformaldehyde for 30 min and stained with 0.1% crystal violet for 5 min at RT. Cells in five visual fields of each well were photographed and counted under a light microscope (Olympus, Japan).

### Xenograft tumor in mice

The animal experiments were conducted in accordance with the ethical regulations of The Affiliated Ganzhou Hospital of Nanchang University and were approved by the experimental committee (SYXK 2021-0004). Four-week-old female BALB/c nude mice were obtained from SLAC Laboratory Animal Co., Ltd (Jiangsu, China) and maintained in a sterile environment with standard laboratory conditions.

For the xenograft tumor assay, MDA-MB-231 cells transfected with shRNA lentivirus (shCtrl, *n* = 5 or shCCNE1, *n* = 5; NC+shCtrl, *n* = 6, shANLN + NC, *n* = 6, CCNE1 + shCtrl, *n* = 6, and CCNE1+shANLN, *n* = 6) were trypsinized, resuspended in DMEM (Sigma-Aldrich, USA) containing 10% fetal bovine serum (FBS, Gibco, USA), and injected subcutaneously into the right forelimb axillary of nude mice at a density of 1 × 10^7^ cells/ml (200 μl per mouse). During the experimental period, body weight of the mice was recorded, and tumor dimensions were measured twice weekly using digital calipers. Tumor volumes (in mm³) were calculated using the formula: *π*/6 × *L* × *W*², where *L* is the long axis and W is the short axis of the tumor. Finally, the mice were anesthetized via intraperitoneal injection of 0.7% pentobarbital sodium at a dose of 10 μL/g and imaged using the Multispectral in Vivo Imaging System (Perkin Elmer, USA).

After the experiment, the mice were euthanized by carbon dioxide inhalation, and tumor tissues were surgically removed. The protein expression levels of CCNE1, ANLN, and stemness markers in the tumor tissues was evaluated by WB analysis using the Amersham Pharmacia Biotech ECL+PlusTM detection system (Amersham Pharmacia Biotech, USA). For the IHC assay, tumor sections were incubated with monoclonal rabbit anti-CCNE1 (1:100; Abcam, USA), anti-ANLN (1:100; Abcam, USA), anti-KI67 (1:200; Abcam, USA) overnight at 4 °C, followed by incubation with goat anti-rabbit secondary antibody conjugated to horseradish peroxidase enzyme (1:100; Abcam, USA) for 1 h at 37 °C in the dark. KI67 expression was then evaluated using the aforementioned immunohistochemistry analysis.

### Data analysis

Each experiment was independently repeated at least three times, and data were presented as mean ± standard deviation (SD). Statistical analyses were performed using SPSS version 23.0 software (Chicago, IL, USA). Differences between two groups were assessed using the unpaired Student’s *t*-test. *P*-values less than 0.05 were considered statistically significant. The Chi-square test, Fisher exact probability, and Spearman correlation analysis were used to evaluate the correlation between CCNE1 expression and clinicopathological features of TNBC.

## Supplementary information

Supplementary figures

Table S1

Table S2

Ethical approval certificate

WB inages

## Data Availability

Data can be obtained through the author
