# Supplementary material for: CCNE1 stabilizes ANLN by counteracting FZR1-mediated the ubiquitination modification to promotes triple negative breast cancer cell stemness and progression
Source: Cell Death Discov. 2025 May 9;11:228. doi: 10.1038/s41420-025-02518-5 (PMC12064766; doi:10.1038/s41420-025-02518-5)
Supplement: Supplementary file 1 — Supplementary figures [file 41420_2025_2518_MOESM1_ESM.docx]

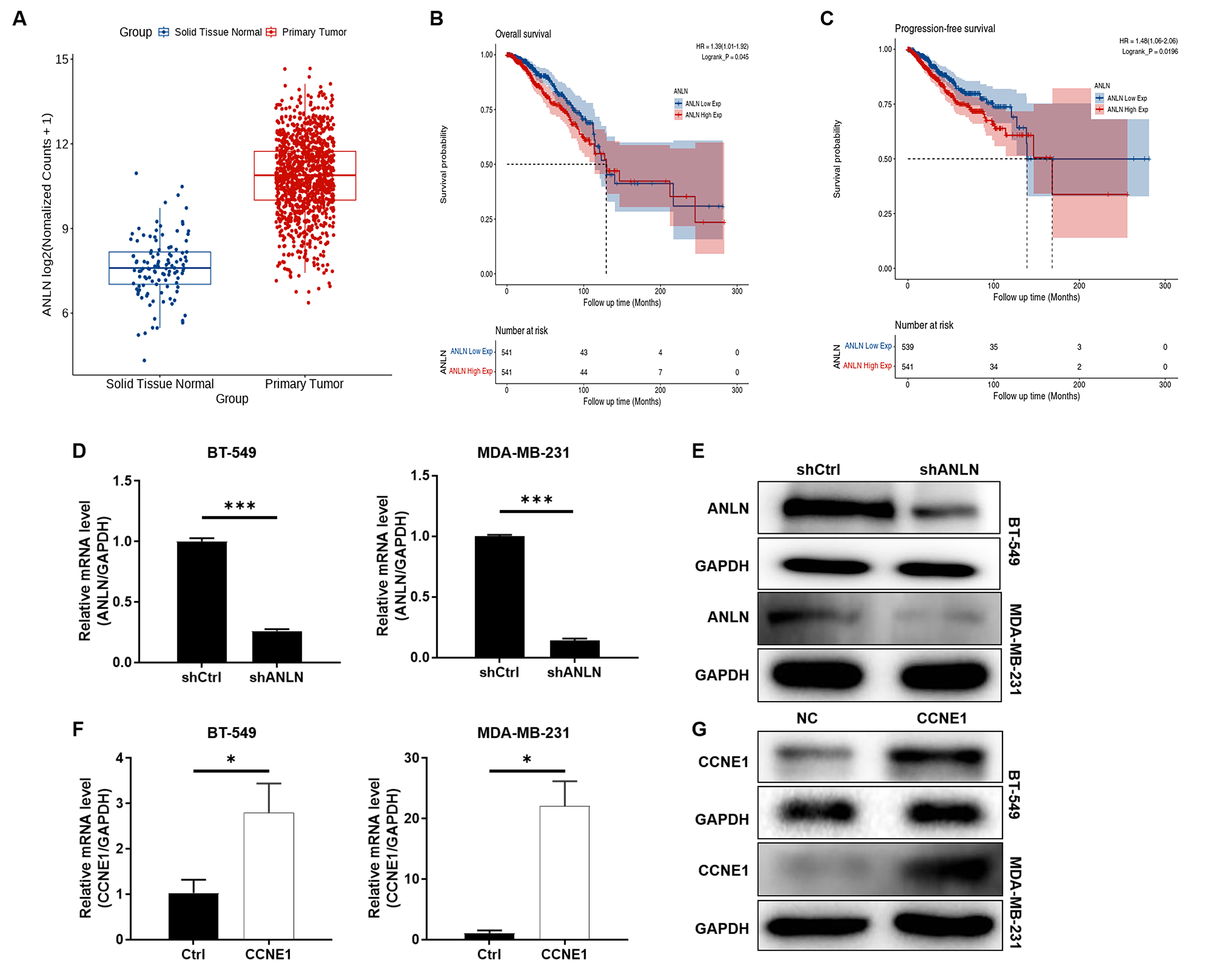


**Fig S1** **Investigation of the regulation of expression among CCNE1, FZR1, ANLN.**

(A) The expression of ANLN in normal and tumor tissues was analyzed based on TCGA-TNBC information. (B-C) Kaplan–Meier analysis of the effect of ANLN expression on the overall survival and progression-free survival of breast cancer patients from TCGA-TNBC. (D-G) The amplified expression efficiency of CCNE1 in breast cancer cell lines on the mRNA and protein levels were evaluated by qRT-PCR and WB analysis, respectively. n=3, *P<0.05, ***P < 0.001.


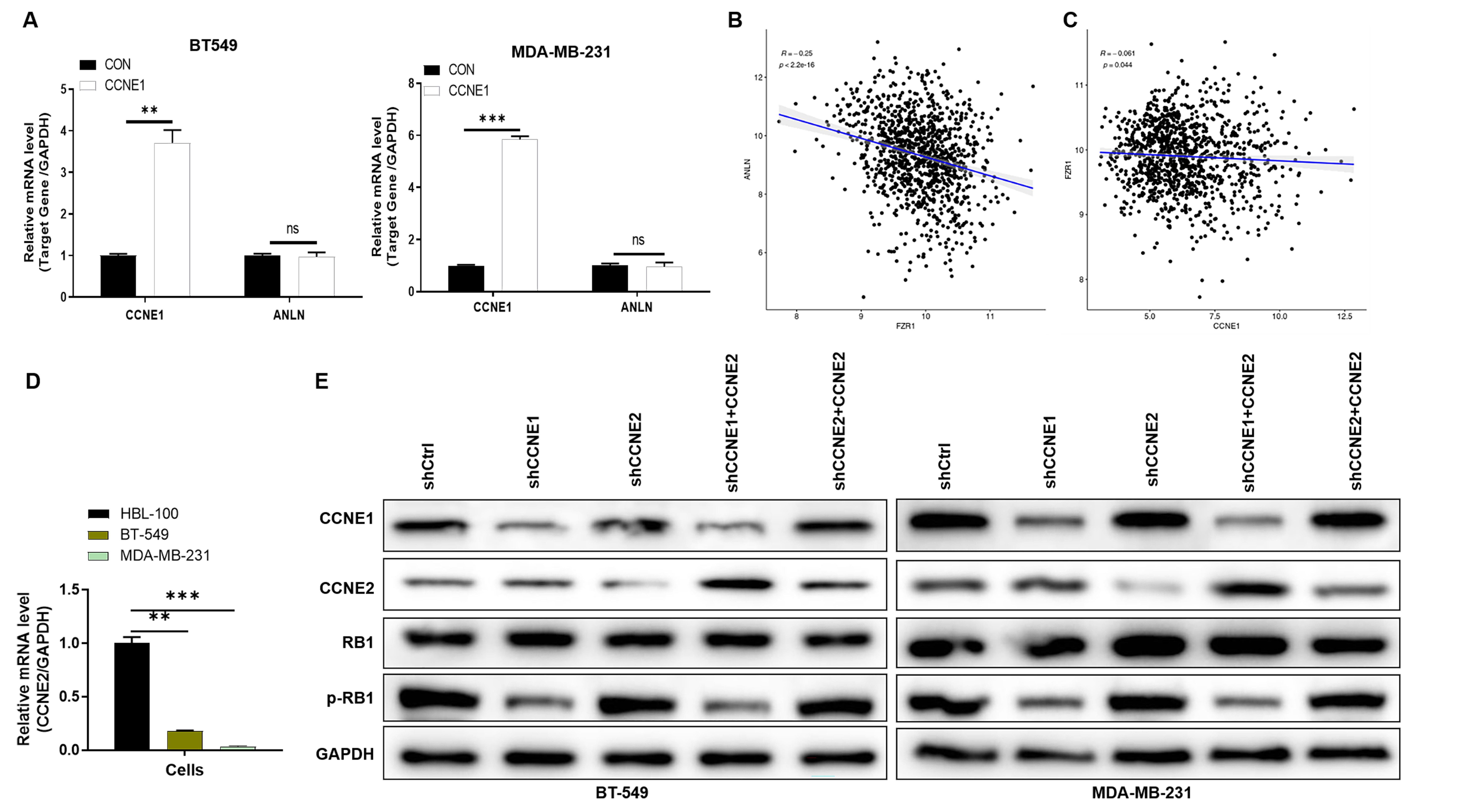


**Fig S2 Investigation of the regulation of expression among CCNE1, CCNE2 ANLN.**

(A) The regulation of ANLN mRNA expression by CCNE1 was detected by qRT-PCR.

(B-C) Pearson correlation analysis from TCGA demonstrated a significant negative correlation between FZR1 and ANLN, as well as CCNE1 and FZR1 expression levels. (D) The expression of CCNE2 in TNBC cell lines BT-549 and MDA-MB-231 and normal breast cell line HBL-100 was analyzed by qRT-PCR. (E) Knockdown CCNE1 and CCNE2 in BT-549 and MDA-MB-231 cells to determine the compensatory level of E2 when knockdown E1 and E2 alone. n=3, ns: no significance, ***P < 0.001.
