# Supplementary material for: CCNE1 stabilizes ANLN by counteracting FZR1-mediated the ubiquitination modification to promotes triple negative breast cancer cell stemness and progression
Source: Cell Death Discov. 2025 May 9;11:228. doi: 10.1038/s41420-025-02518-5 (PMC12064766; doi:10.1038/s41420-025-02518-5)
Supplement: Supplementary file 2 — Table S1 [file 41420_2025_2518_MOESM2_ESM.docx]

| **Table S1 Antibody information used in this study** | | | | | |
| --- | --- | --- | --- | --- | --- |
| Primary antibody | Size (kDa) | Dilution ratio | Source | Corporation | Article No. |
| CCNE1 | 47 | 1/1000 | Rabbit | Proteintech | 11554-1-AP |
| ANLN | 120 | 1/1000 | Rabbit | ABclonal | A6524 |
| Ubiquitin | / | 1/2000 | Mouse | Santa Cruz | sc-8017 |
| SOX2 | 34 | 1/500 | Mouse | Santa Cruz | sc-365823 |
| CD44 | 82 | 1/2000 | Rabbit | Proteintech | 15675-1-AP |
| CD133 | 97/120 | 1/1000 | Rabbit | Abcam | ab216323 |
| β-catenin | 86 | 1/2000 | Rabbit | Proteintech | 51067-2-AP |
| GSK-3β | 46 | 1/1000 | Rabbit | CST | 12456S |
| p-GSK-3β | 48 | 1/1000 | Mouse | Proteintech | 67558-1-Ig |
| GAPDH | 36 | 1/3000 | Mouse | Proteintech | 60004-1-lg |
| Secondary antibody | / | Dilution ratio | Source | Corporation | Article No. |
| Goat Anti-Rabbit | / | 1/3000 | Rabbit | Beyotime | A0208 |
| Goat Anti-Mouse | / | 1/3000 | Mouse | Beyotime | A0216 |
|  |  |  |  |  |  |
