# Supplementary figures and images for: CCNE1 stabilizes ANLN by counteracting FZR1-mediated the ubiquitination modification to promotes triple negative breast cancer cell stemness and progression
Source: Cell Death Discov. 2025 May 9;11:228. doi: 10.1038/s41420-025-02518-5 (PMC12064766; doi:10.1038/s41420-025-02518-5)

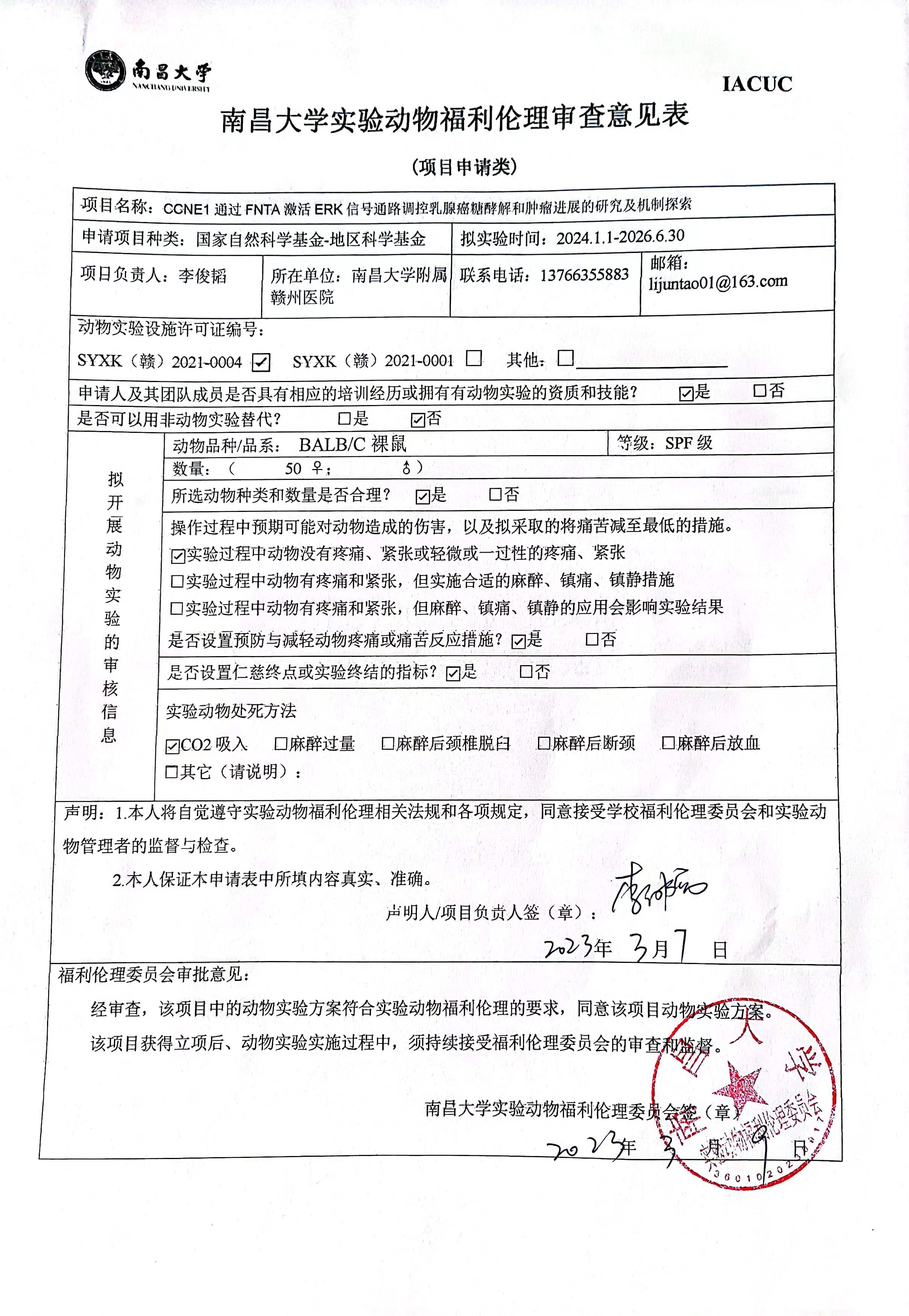

Supplement: Supplementary file 4 — Ethical approval certificate [file 41420_2025_2518_MOESM4_ESM.jpg]
