# Supplementary material for: CCNE1 stabilizes ANLN by counteracting FZR1-mediated the ubiquitination modification to promotes triple negative breast cancer cell stemness and progression
Source: Cell Death Discov. 2025 May 9;11:228. doi: 10.1038/s41420-025-02518-5 (PMC12064766; doi:10.1038/s41420-025-02518-5)

BT-549

CCNE1

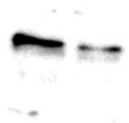

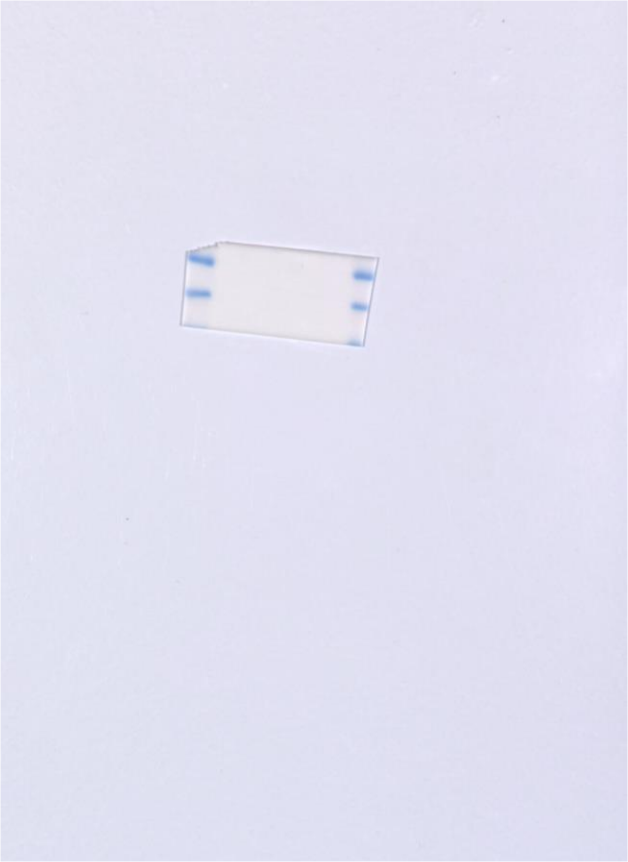

GAPDH

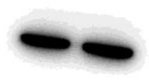

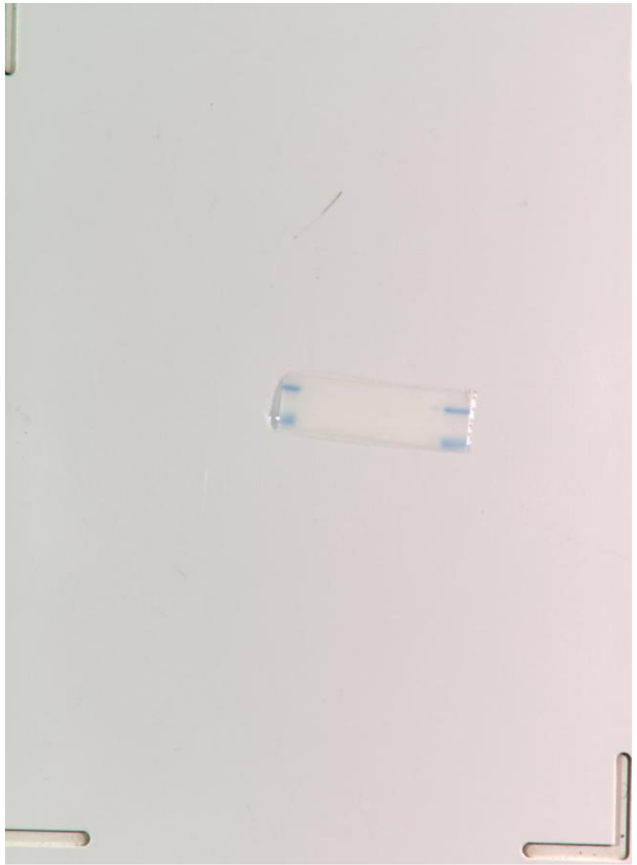

MDA-MB-231

CCNE1

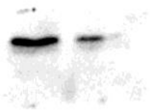

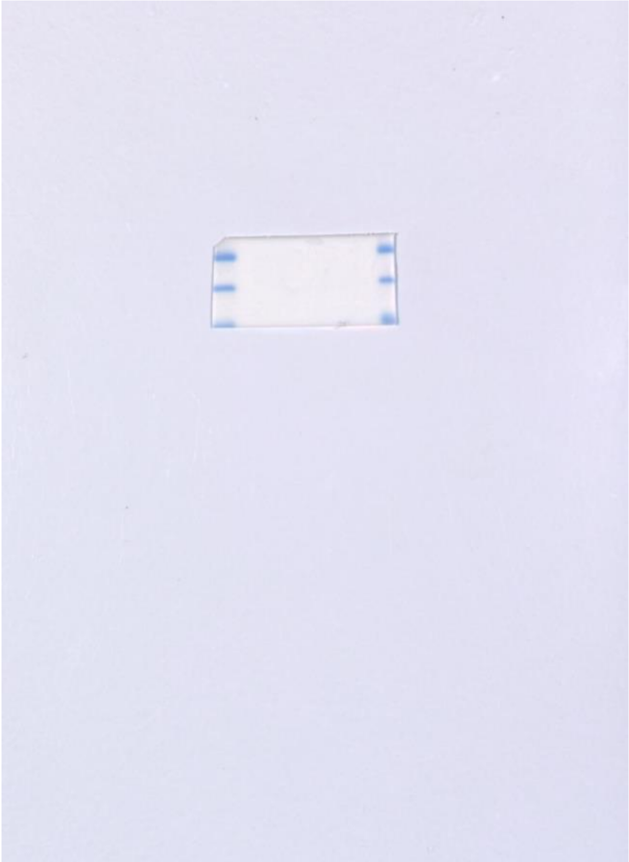

GAPDH

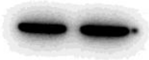

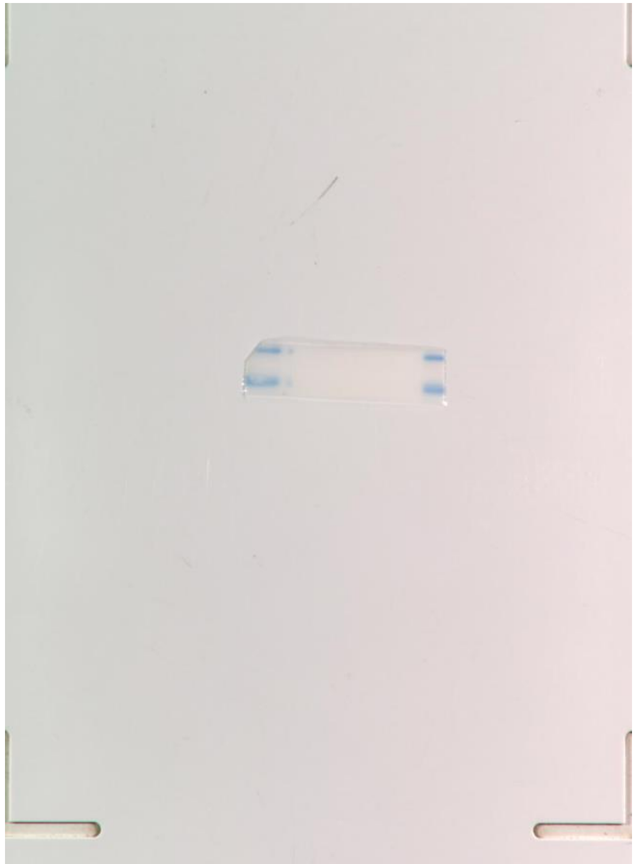

CCNE1

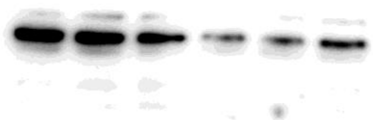

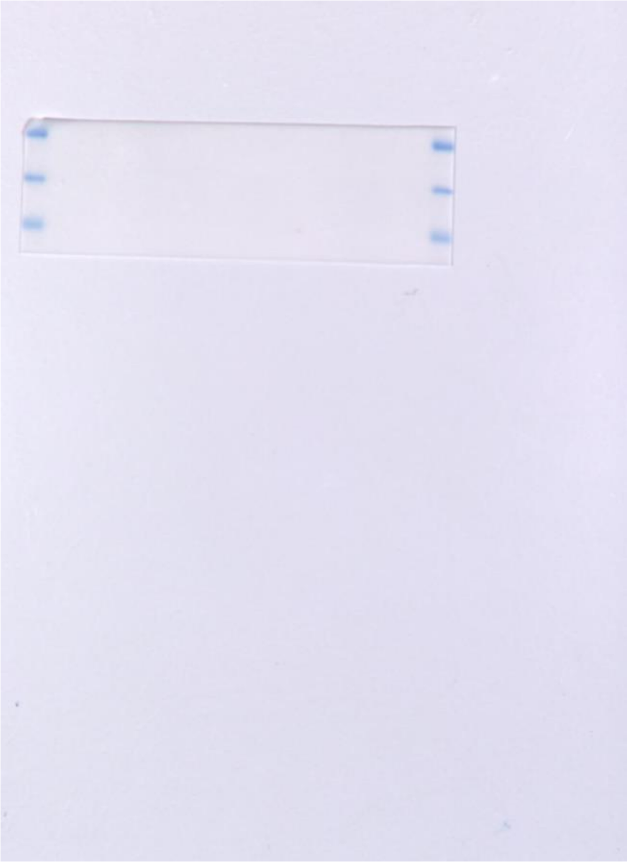

GAPDH

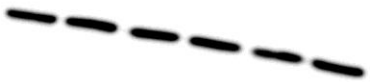

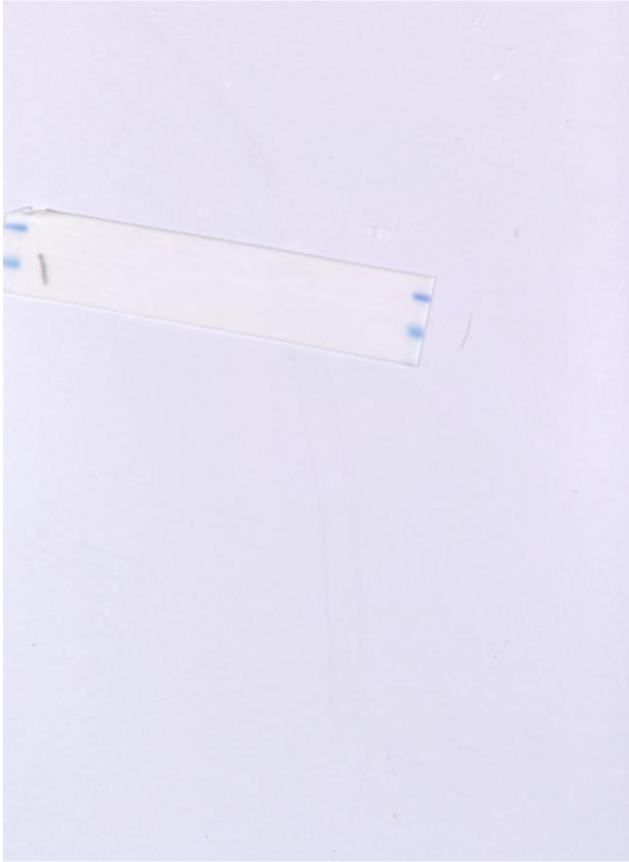

BT549

CD44

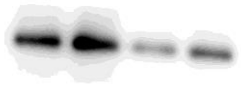

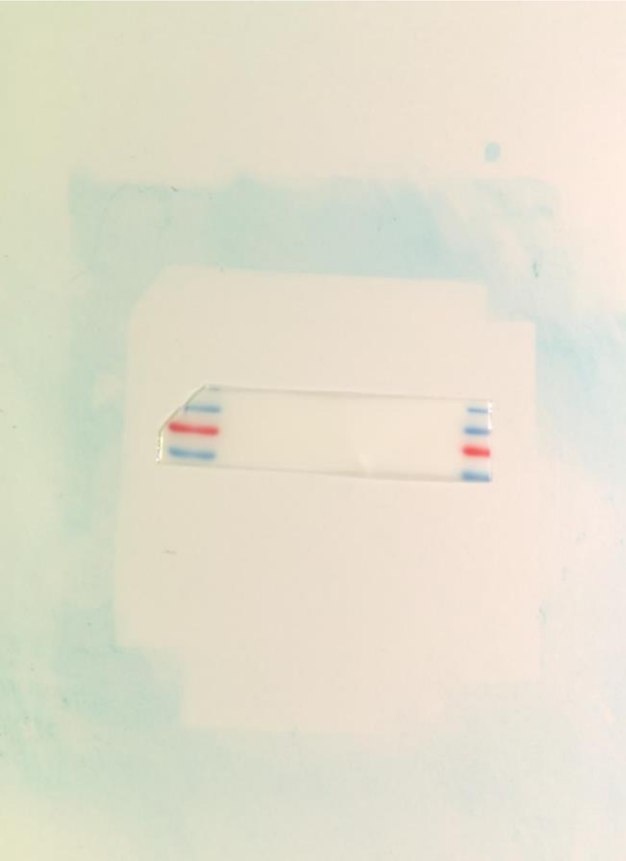

MDA-MB-231

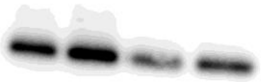

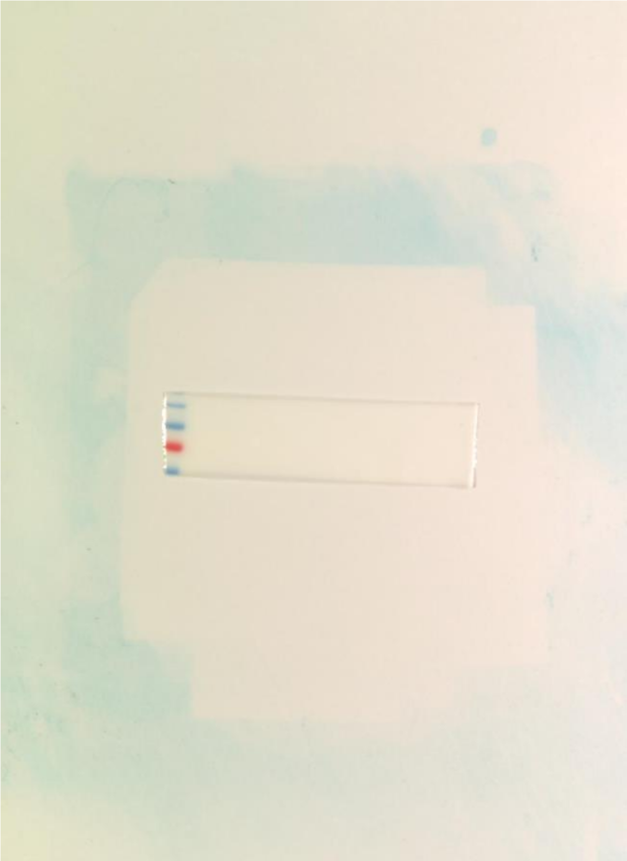

BT549

MDA-MB-231

CD133

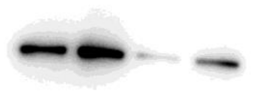

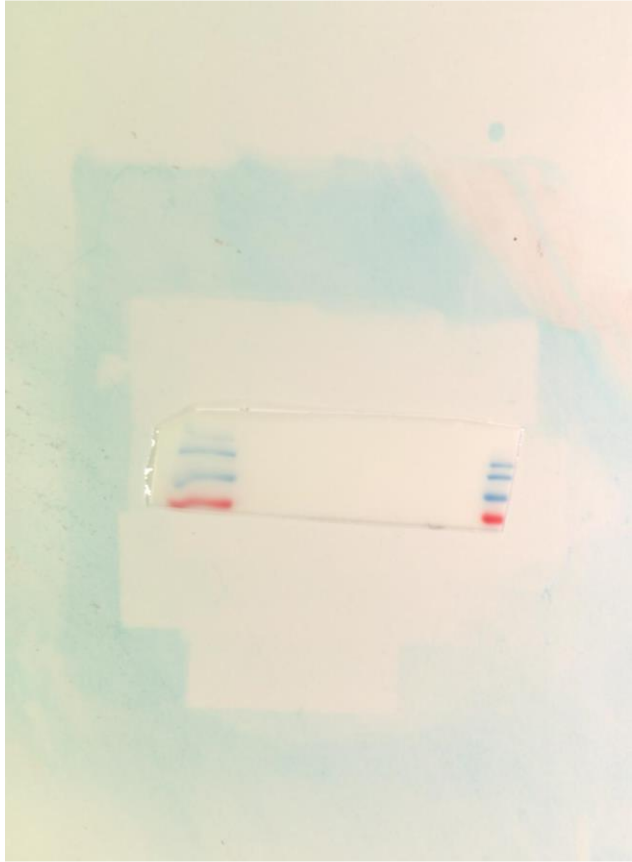

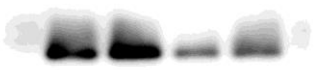

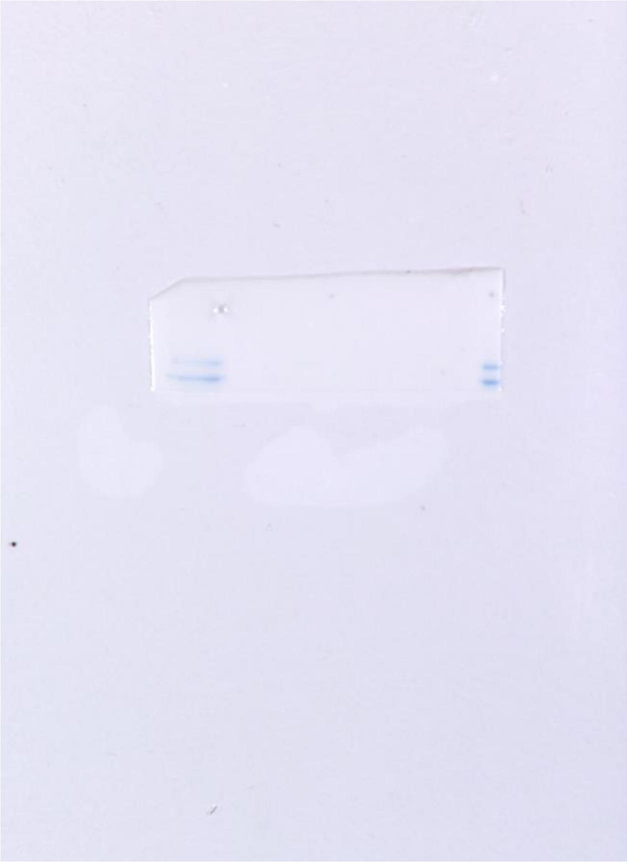

BT549

SOX2

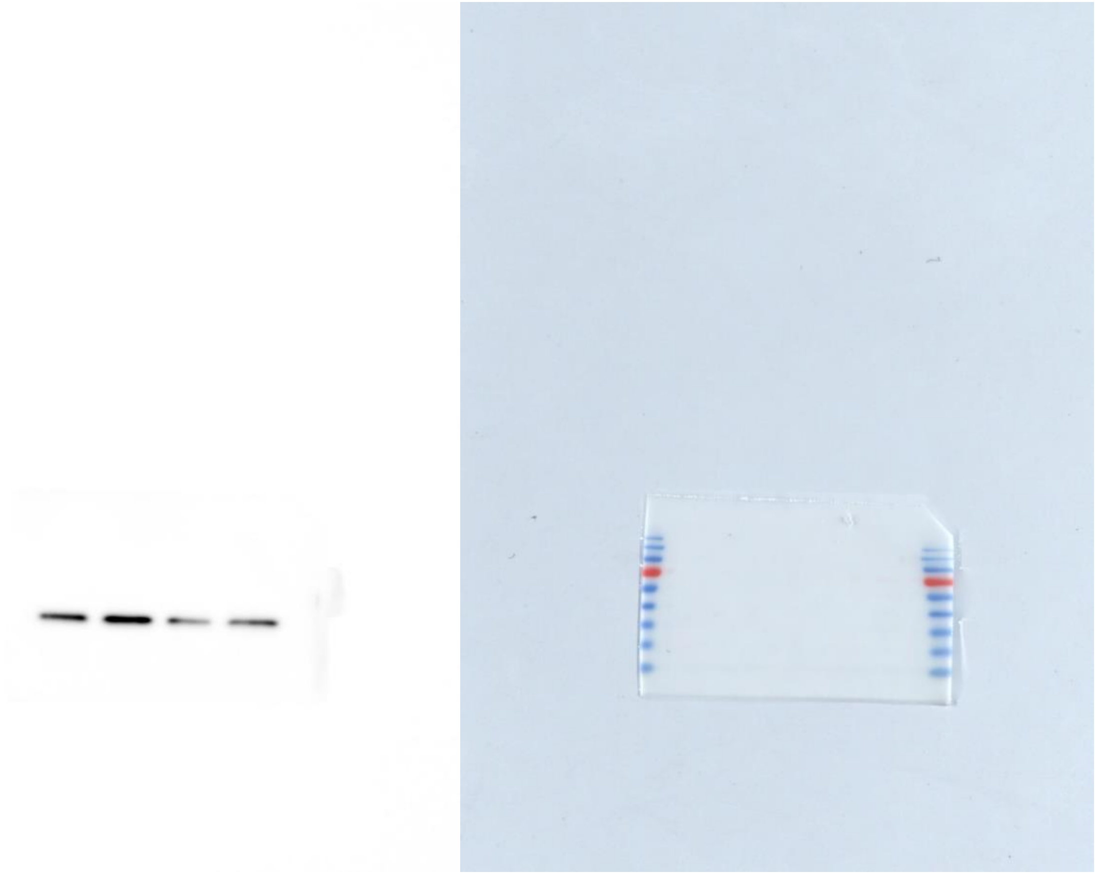

MDA-MB-231

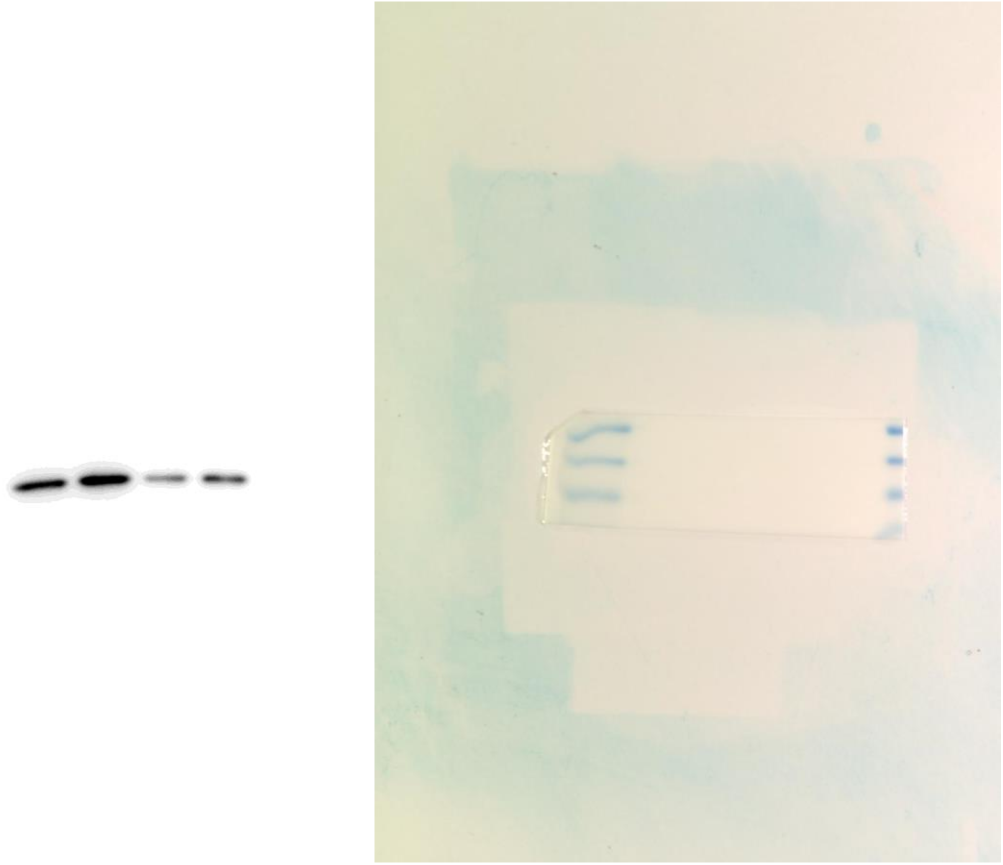

BT549

GAPDH

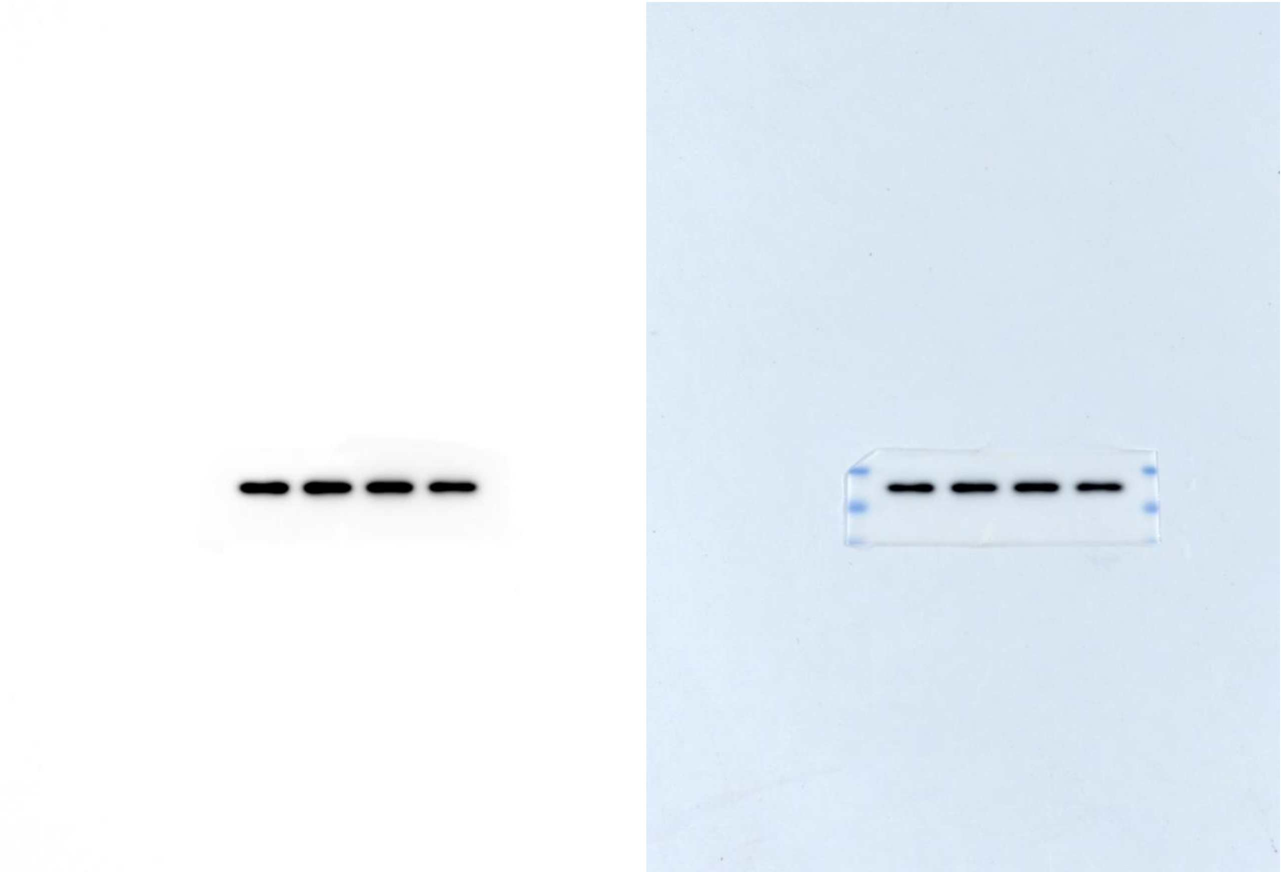

MDA-MB-231

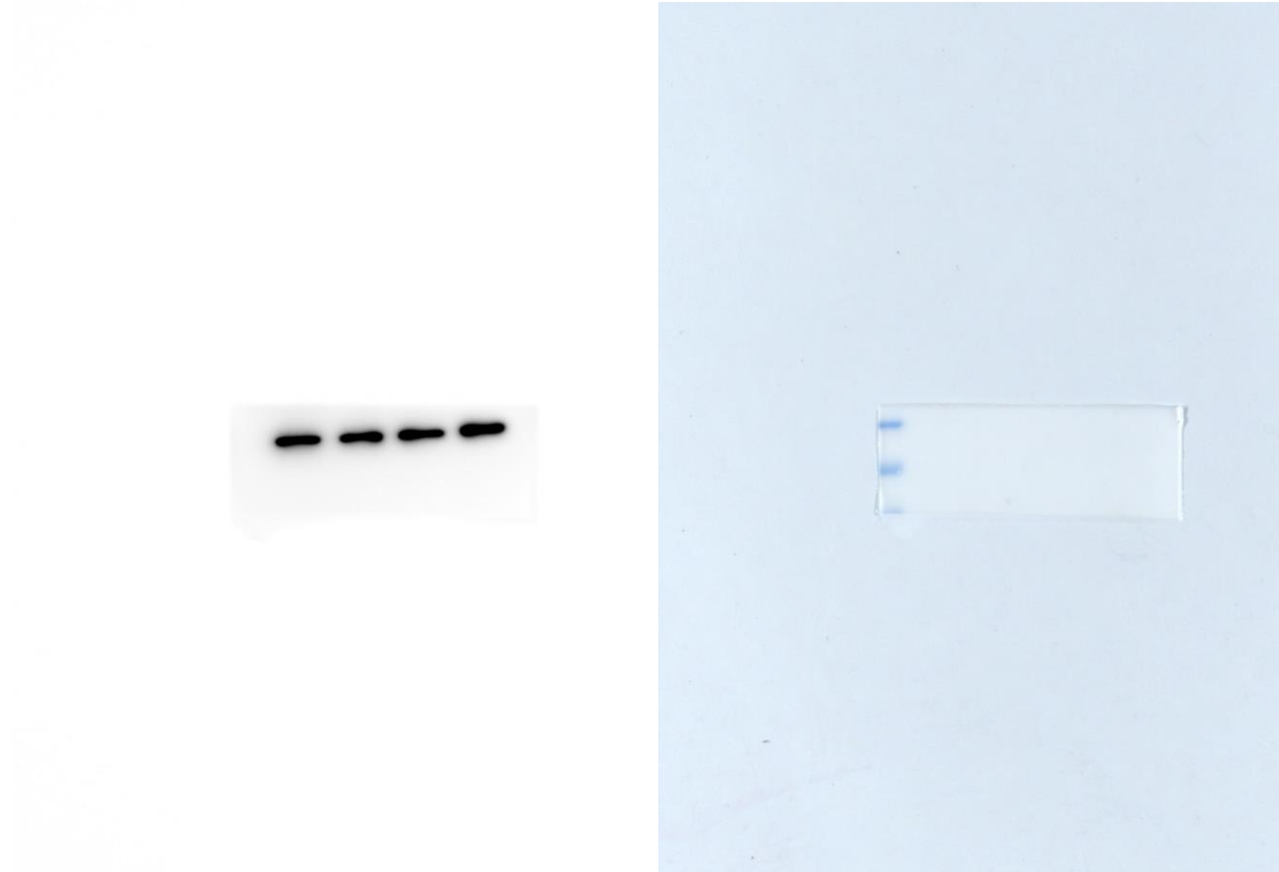

BT549

GSK-3β

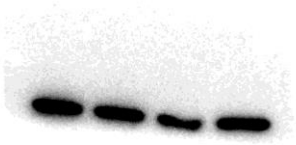

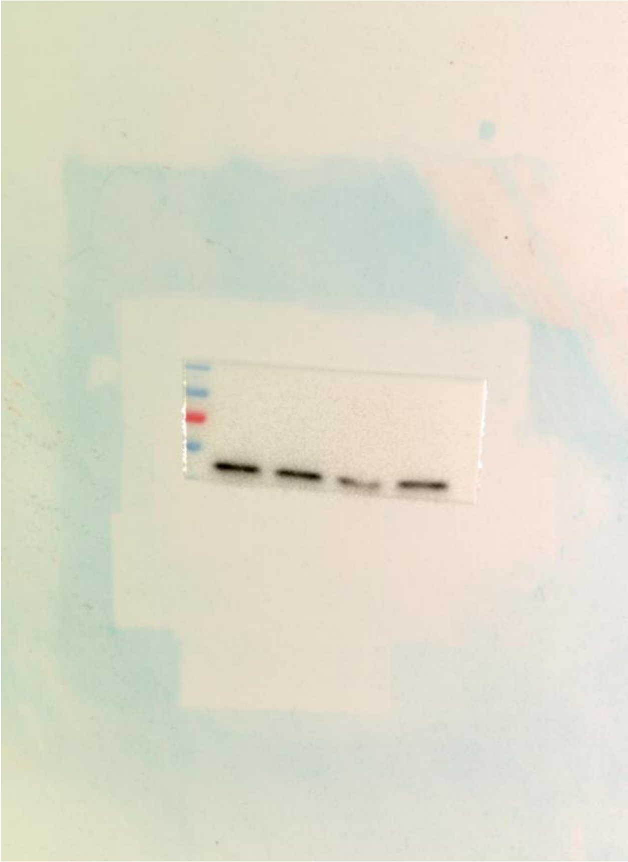

MDA-MB-231

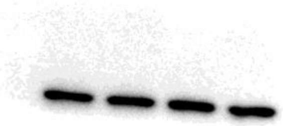

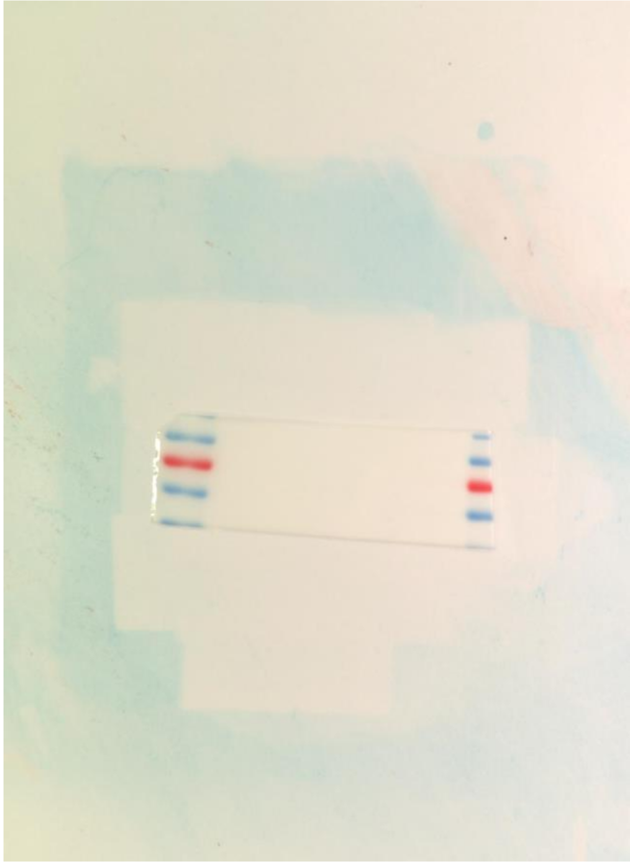

BT549

MDA-MB-231

p-GSK-3β

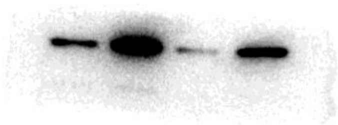

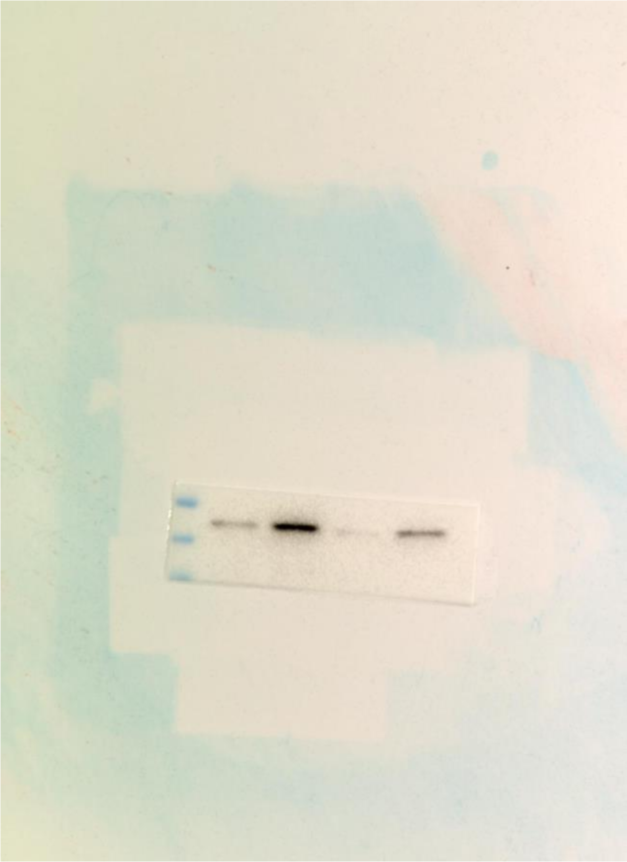

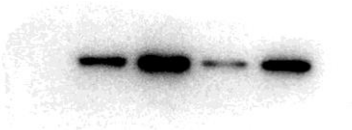

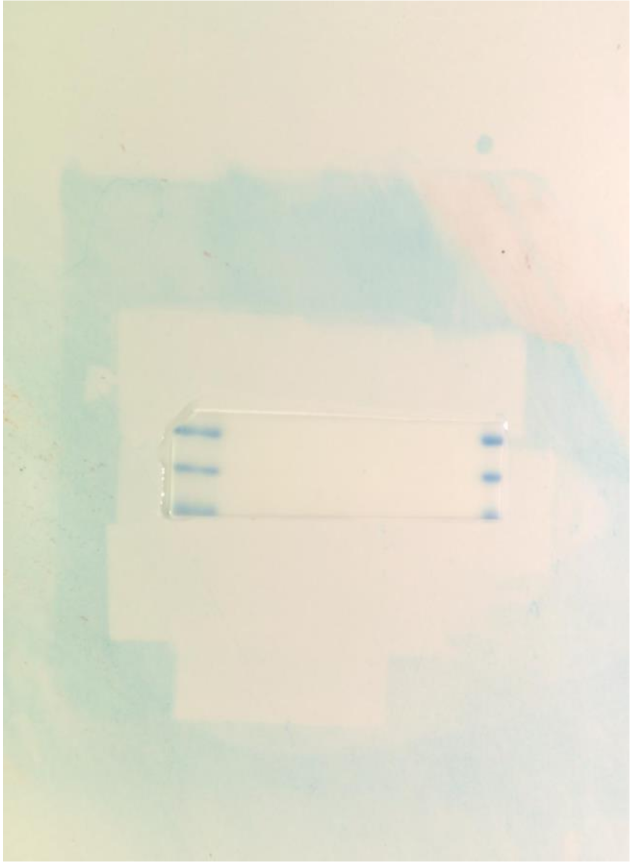

BT549

MDA-MB-231

β-catenin

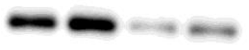

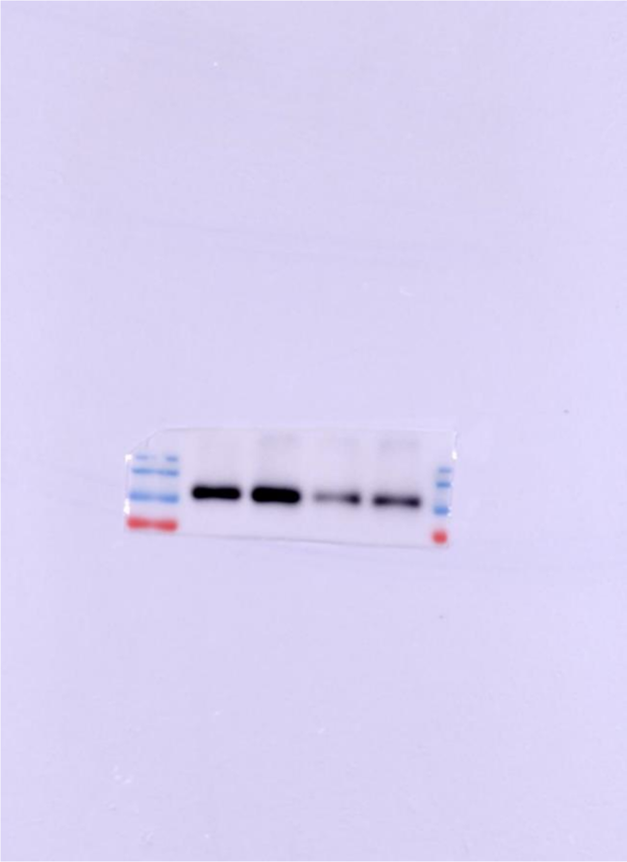

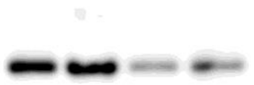

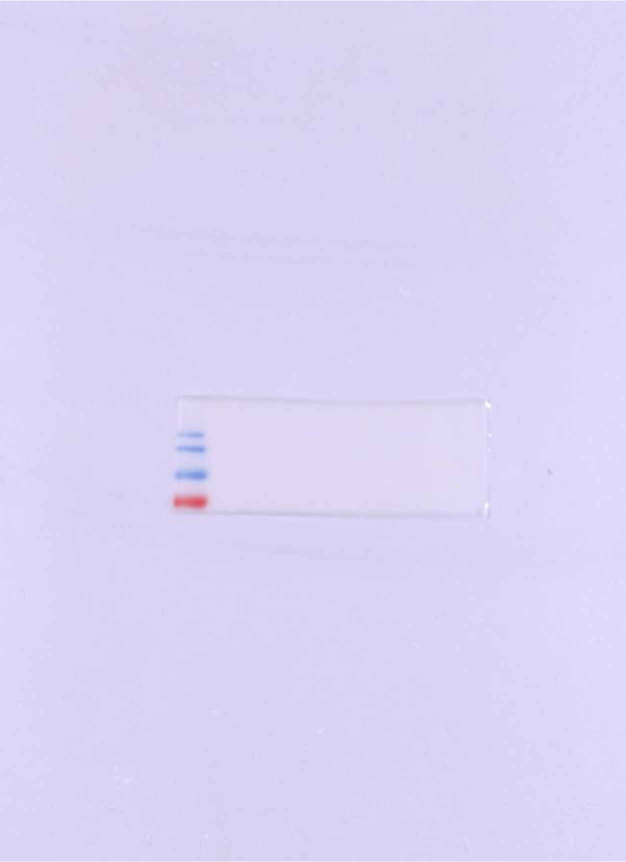

ANLN

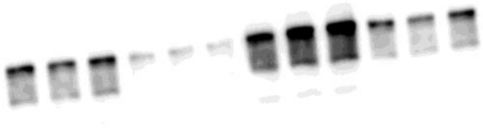

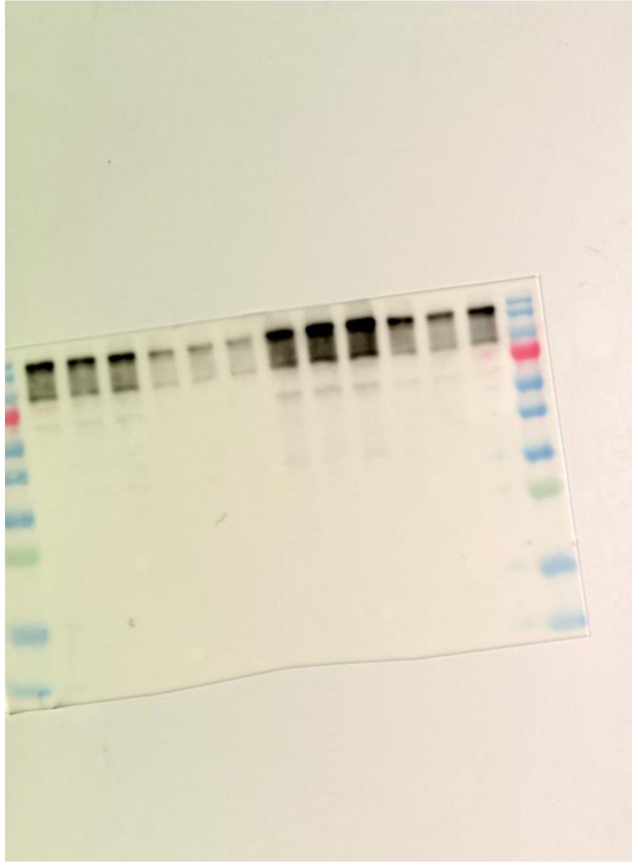

CCNE1

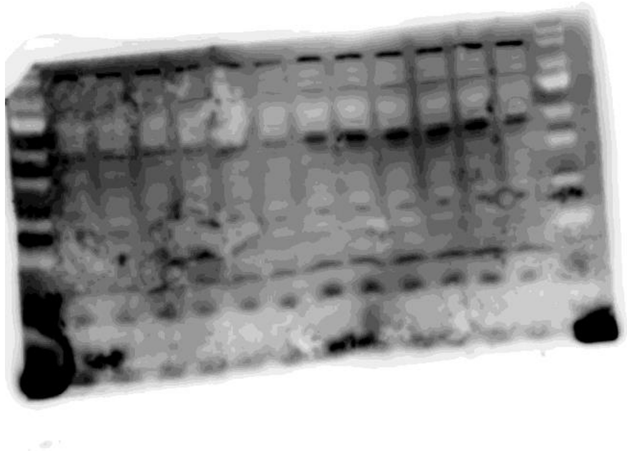

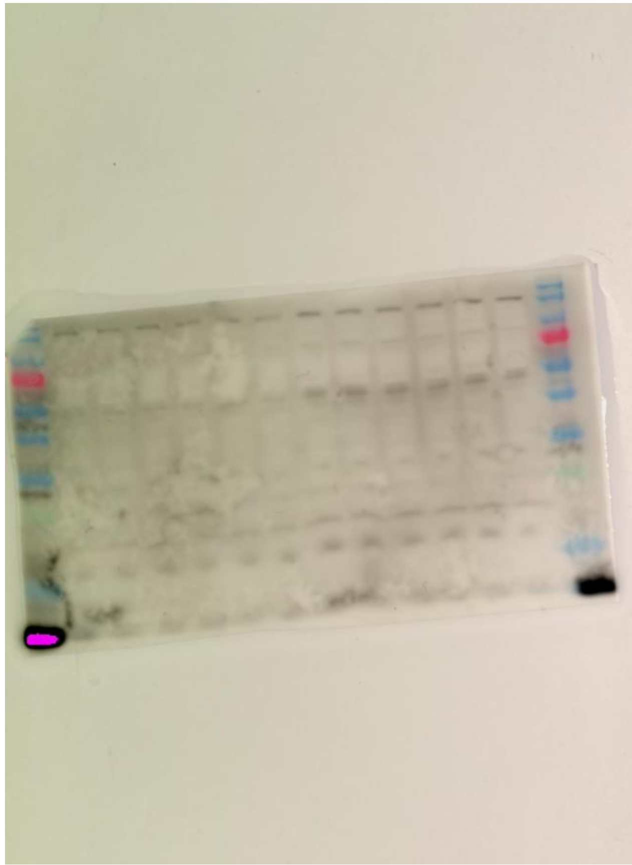

CD44

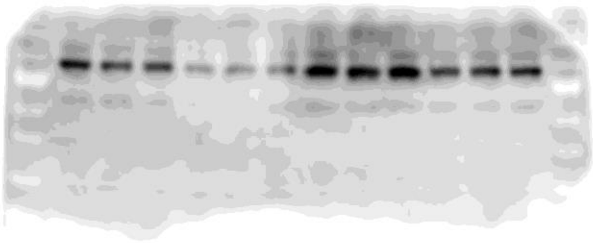

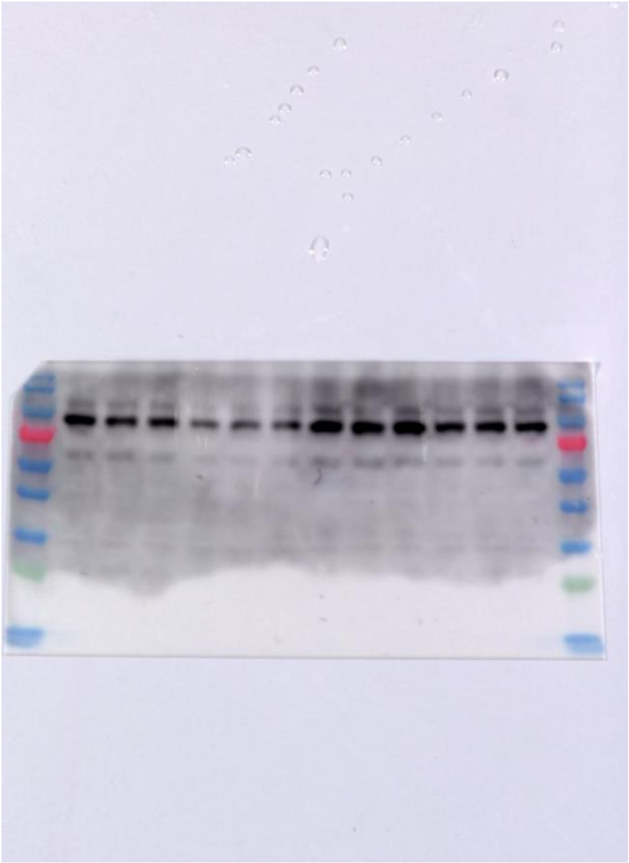

CD133

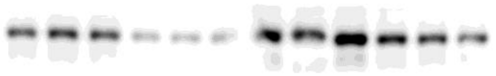

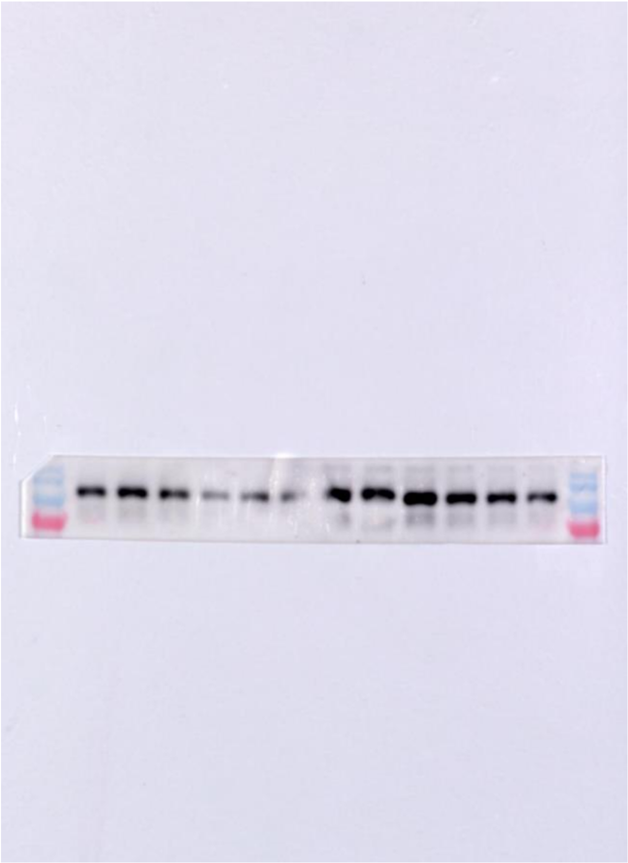

GSK3β

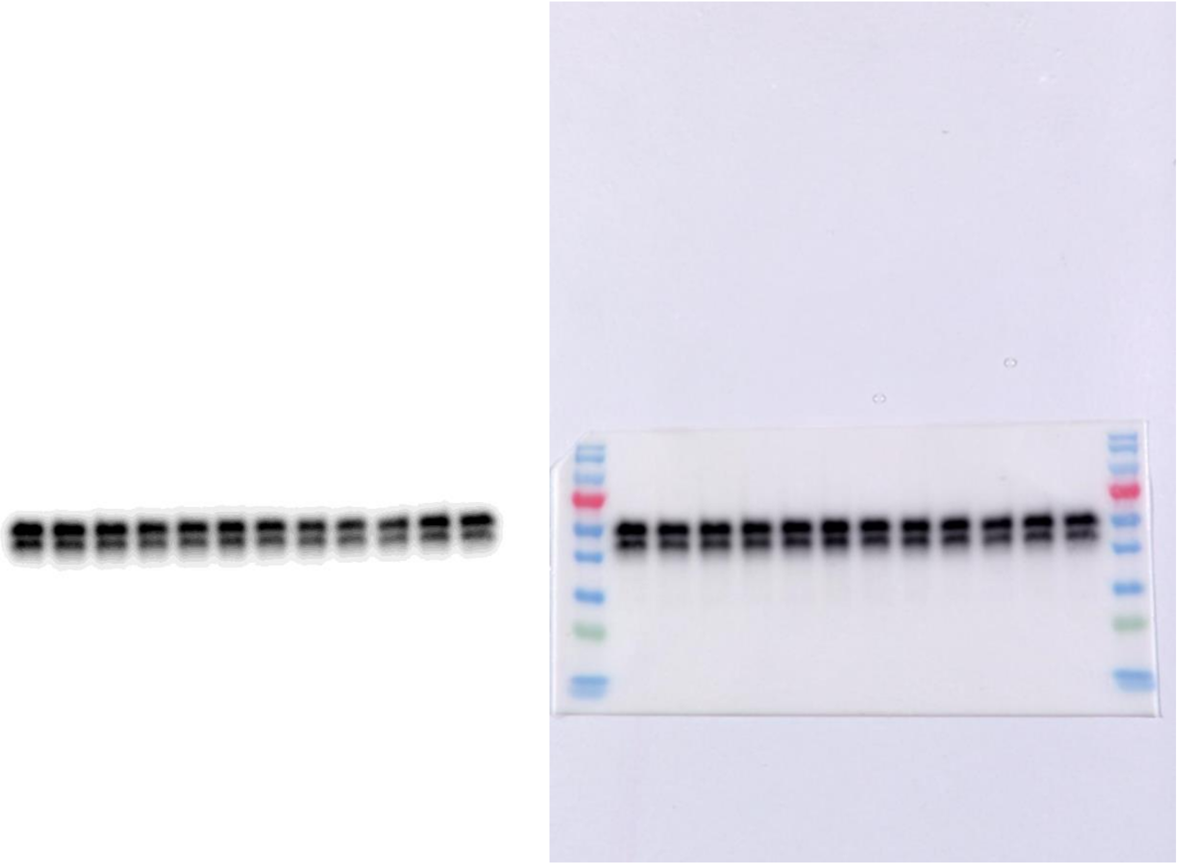

p-GSK3β

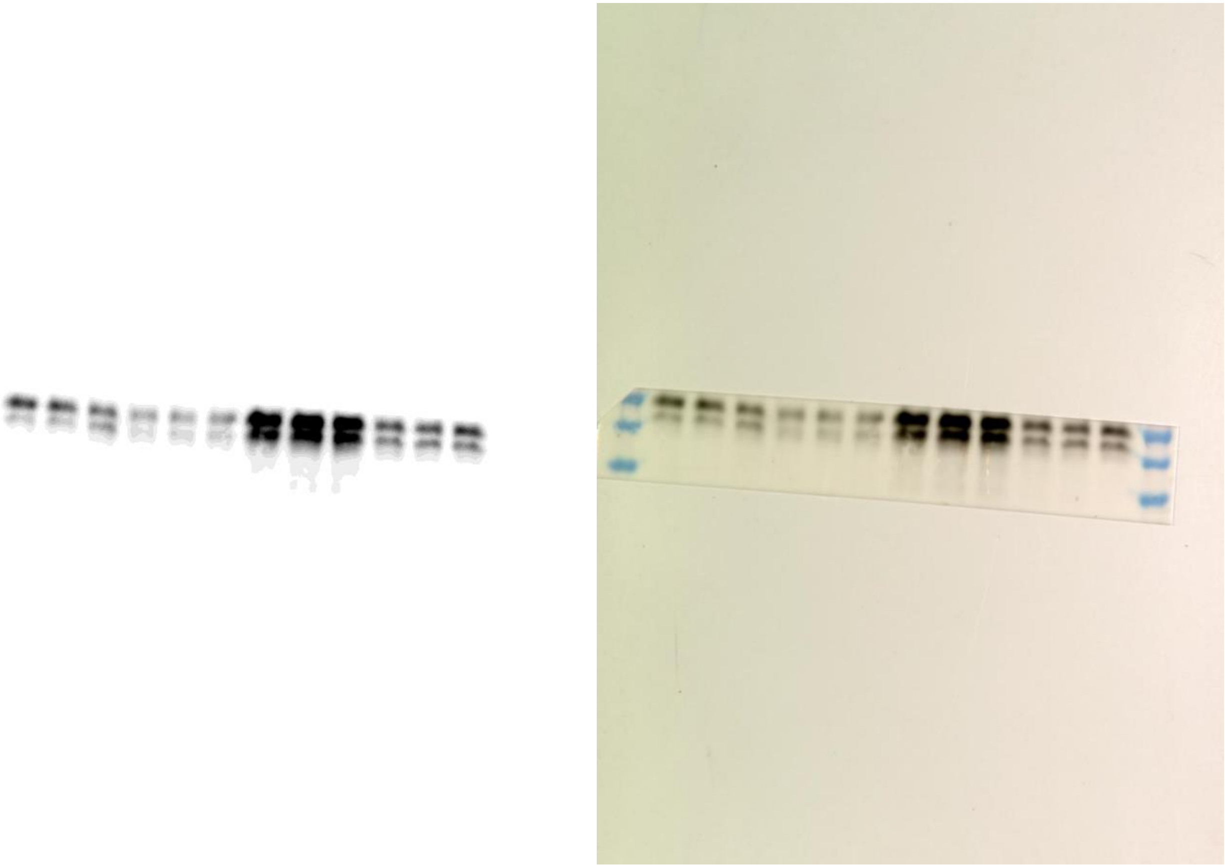

SOX2

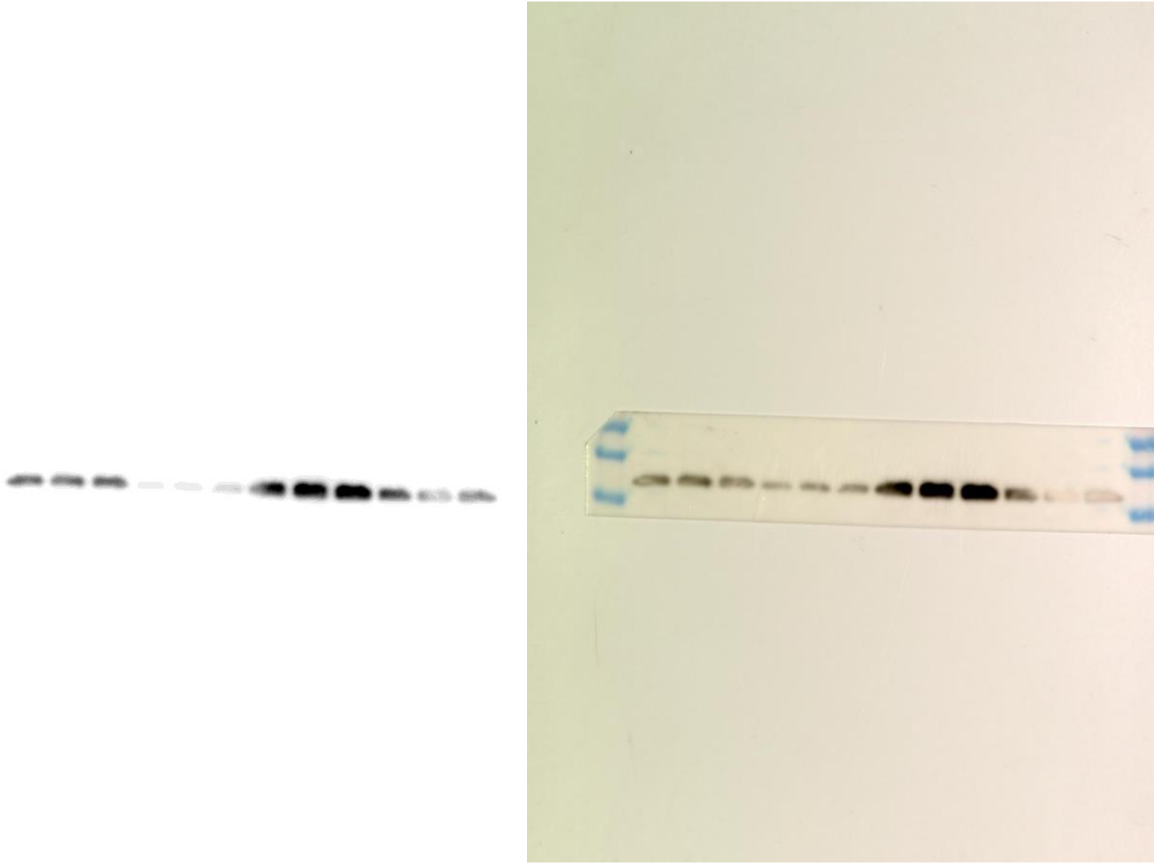

β-catenin

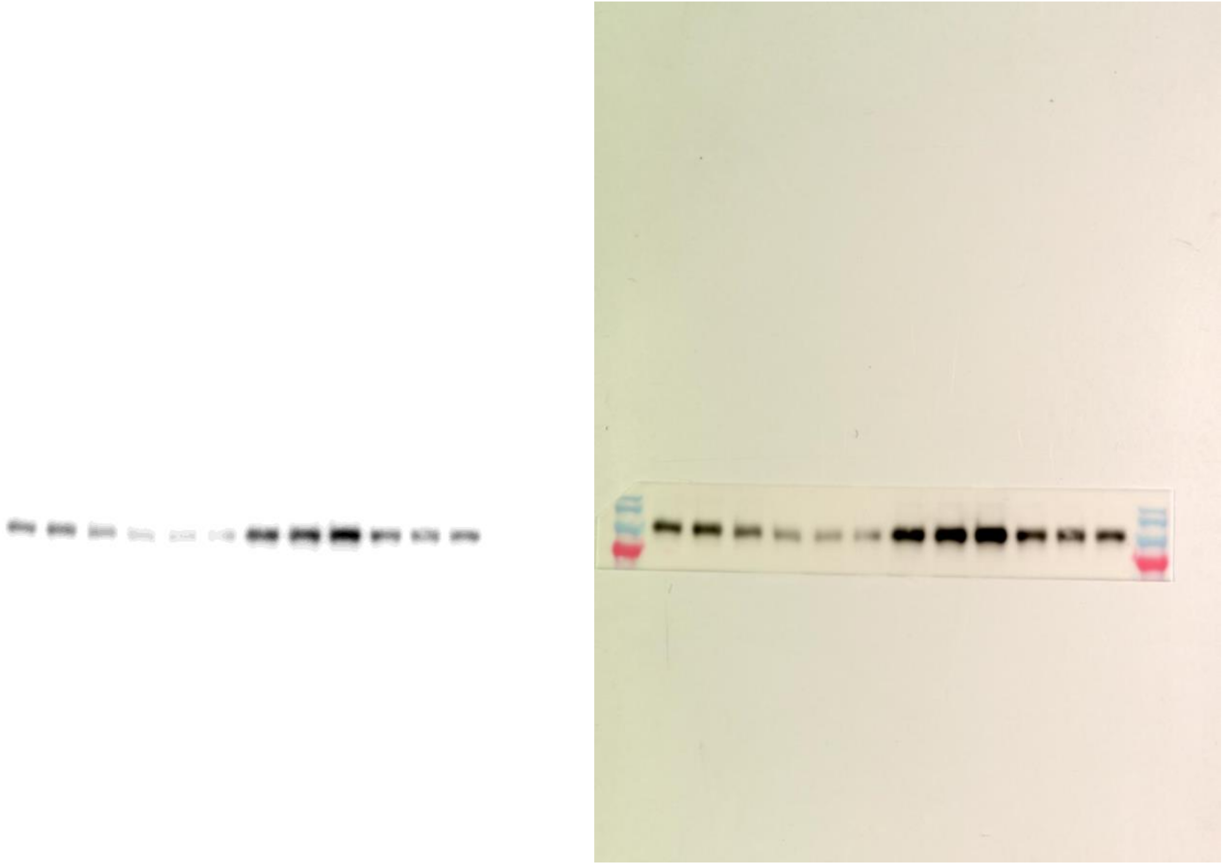

GAPDH

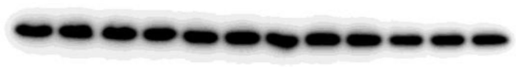

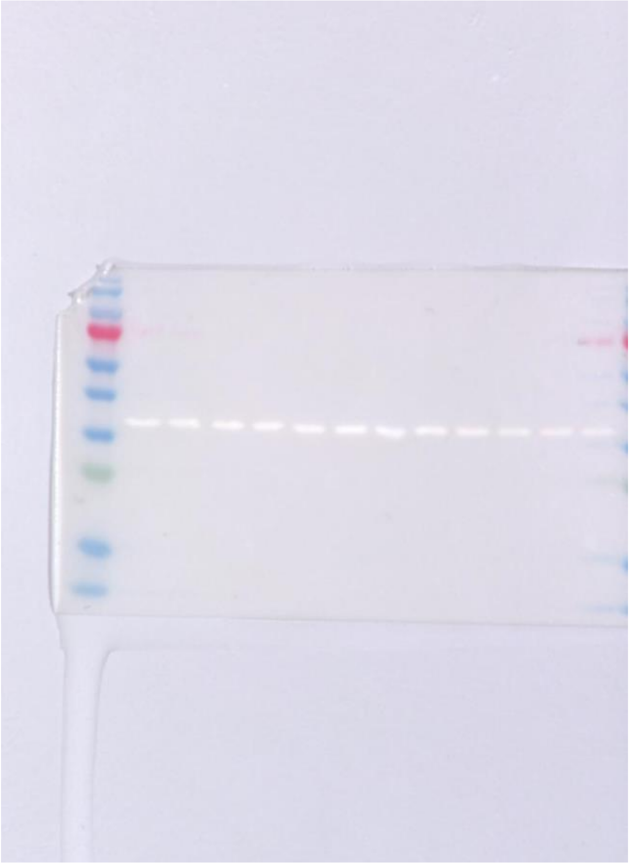

BT549

ANLN

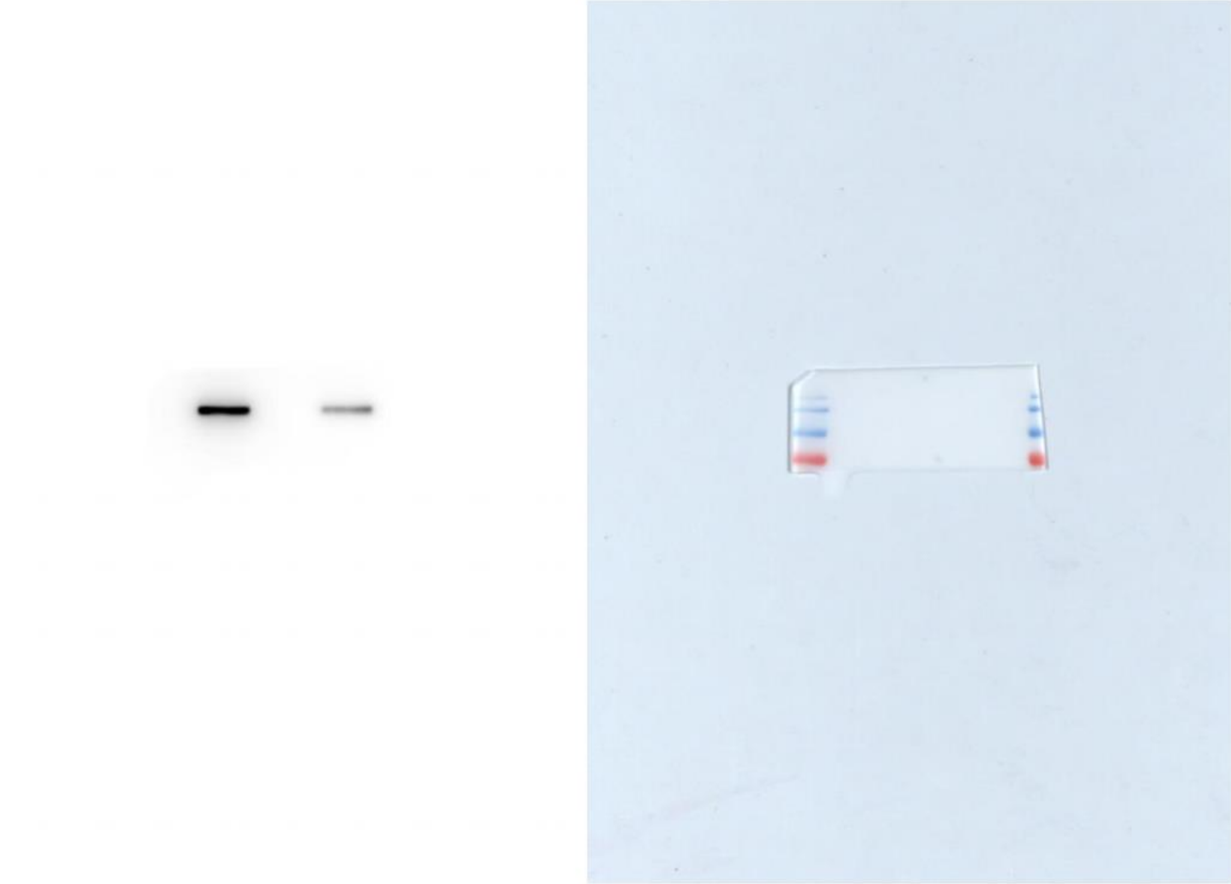

CCNE1

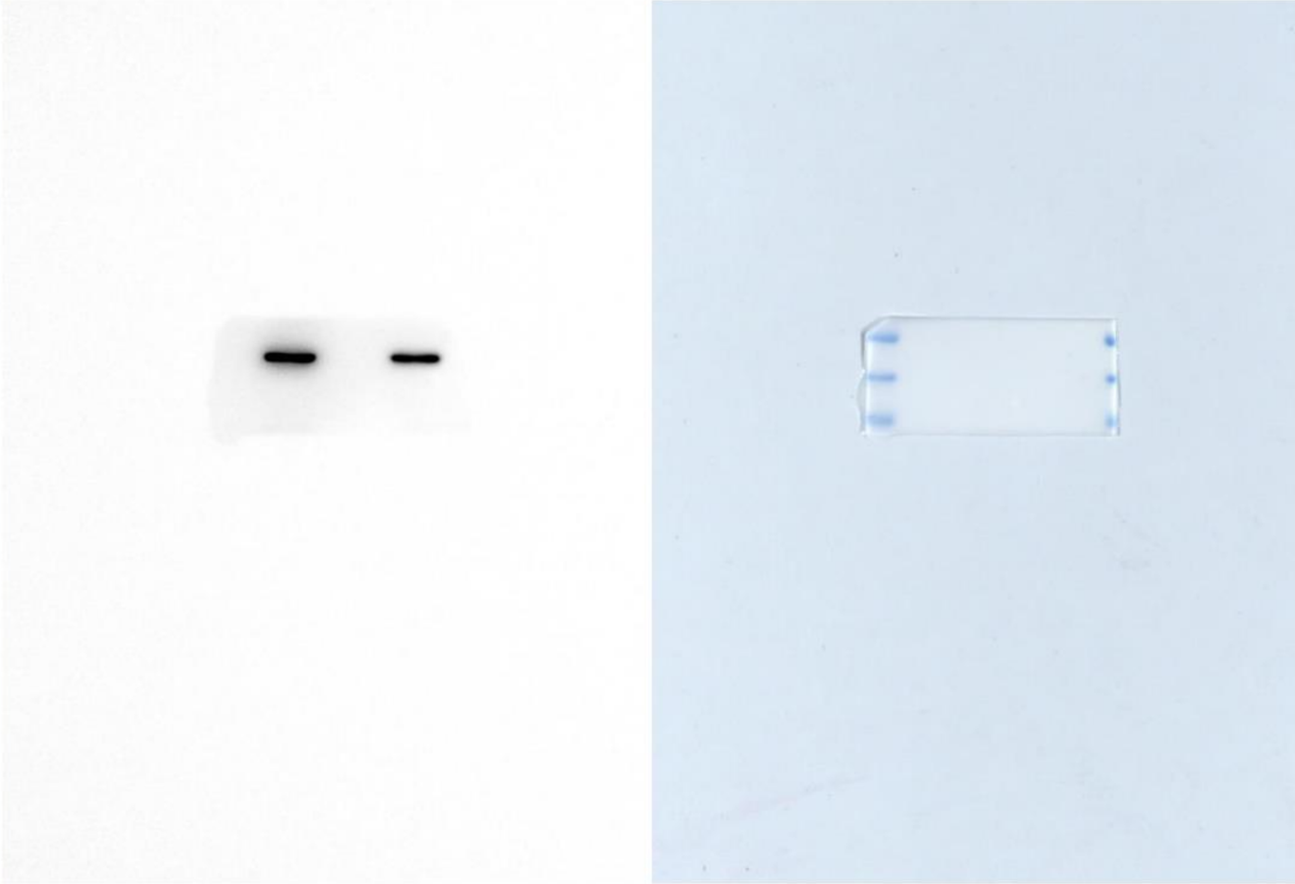

Left panel

MDA-MB-231

ANLN

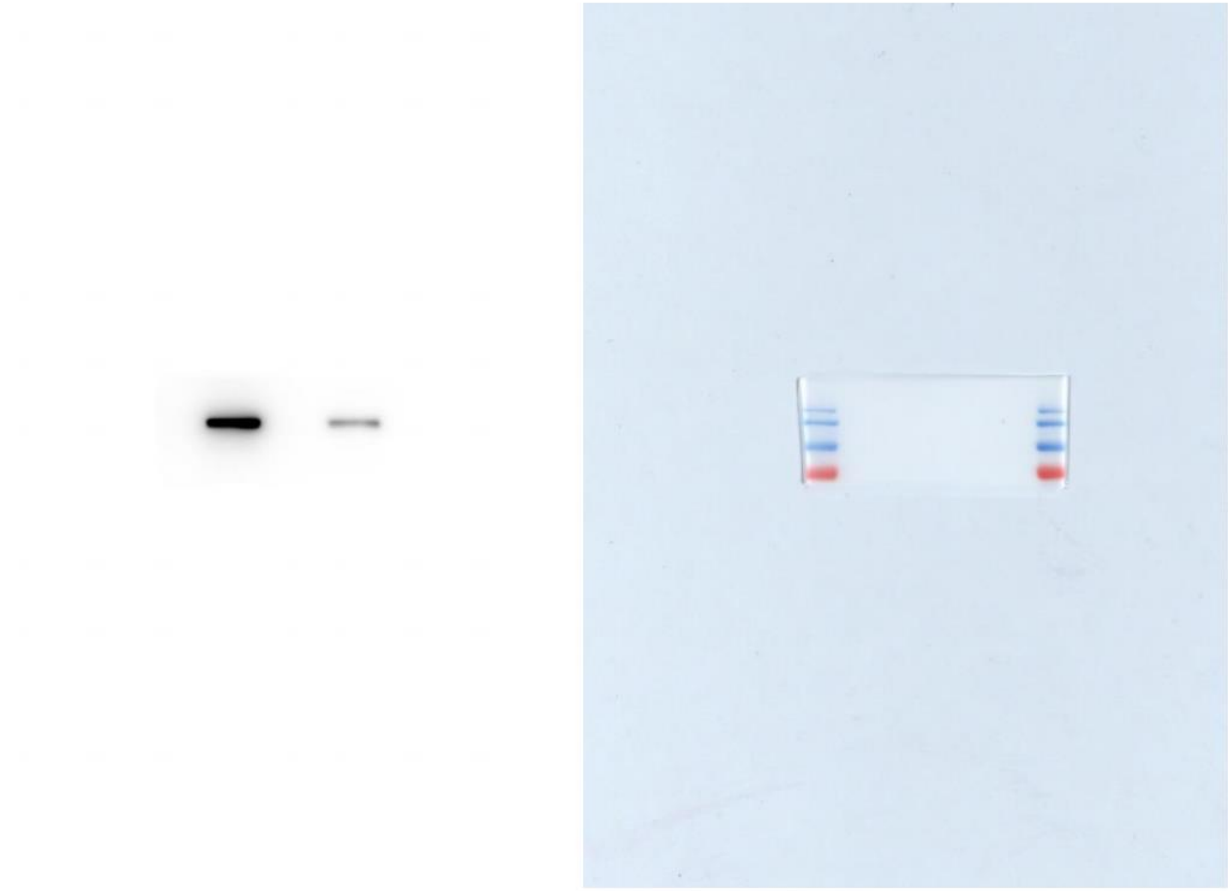

CCNE1

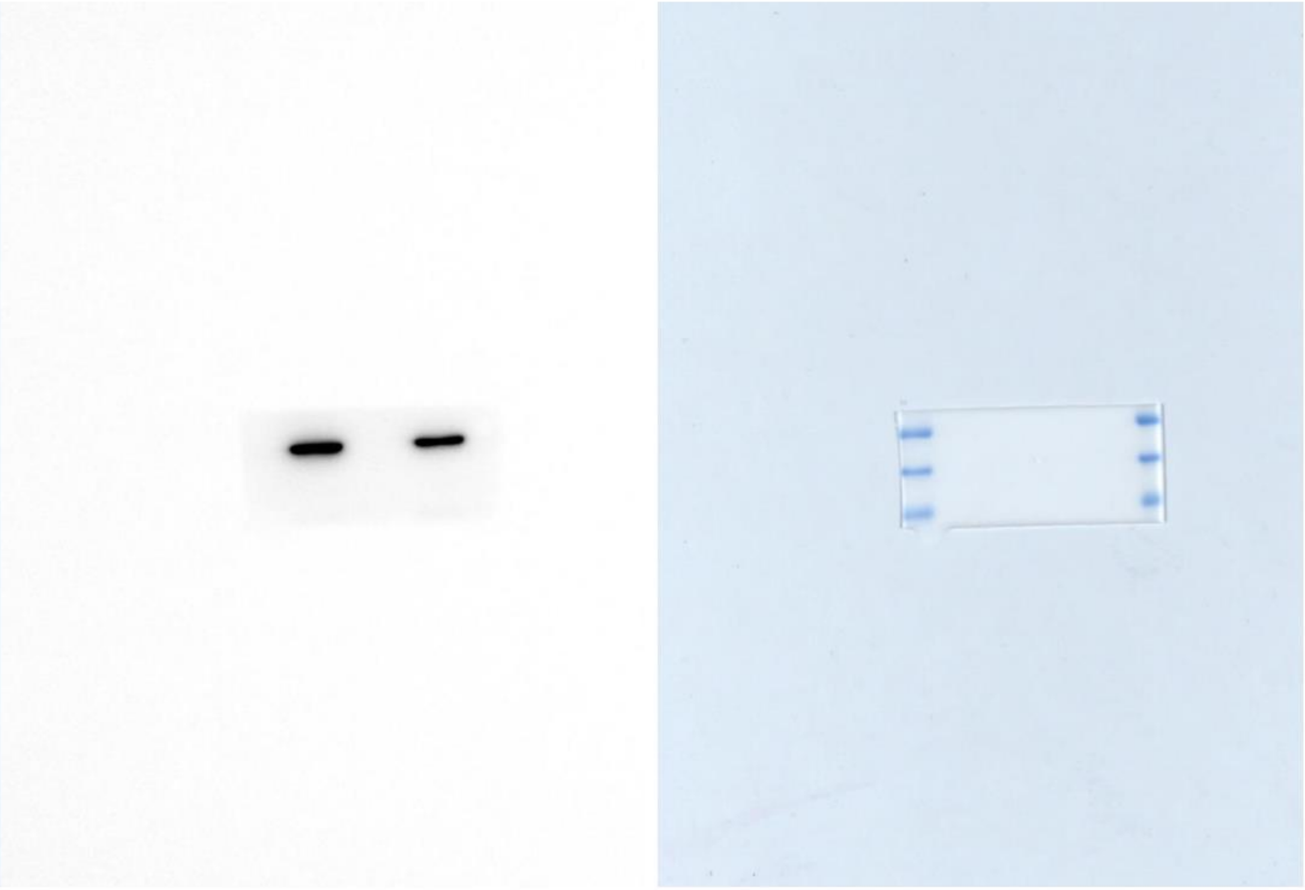

Left panel

BT-549

ANLN

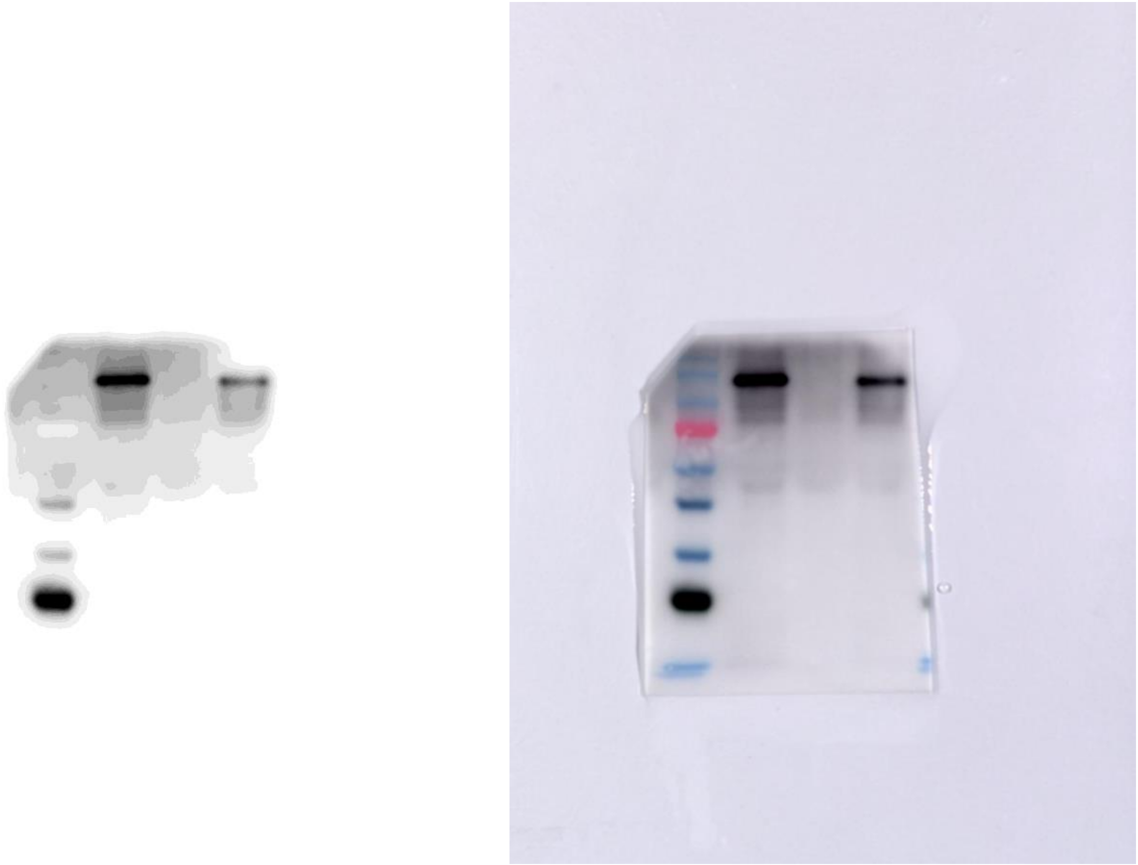

MDA-MB-231

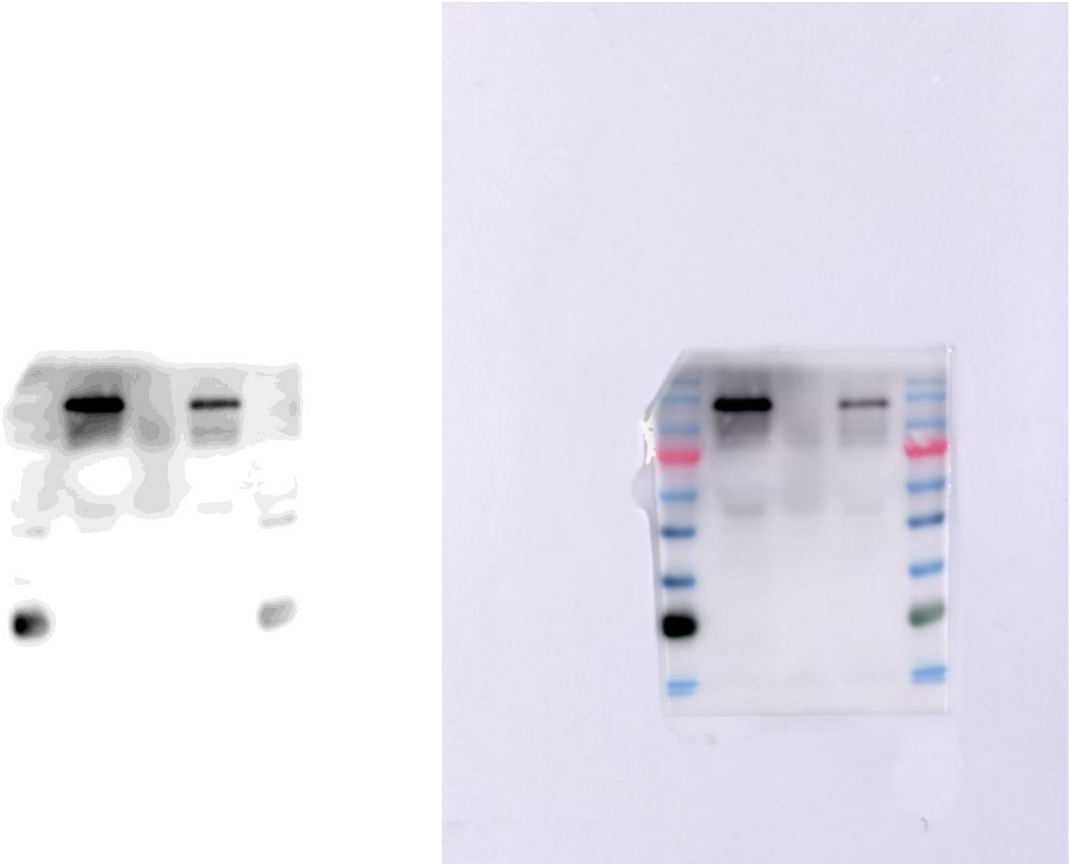

Right panel

BT-549

MDA-MB-231

CCNE1

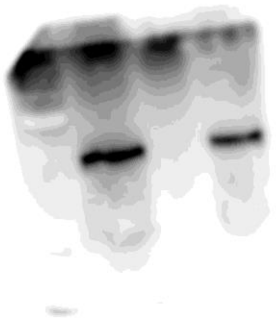

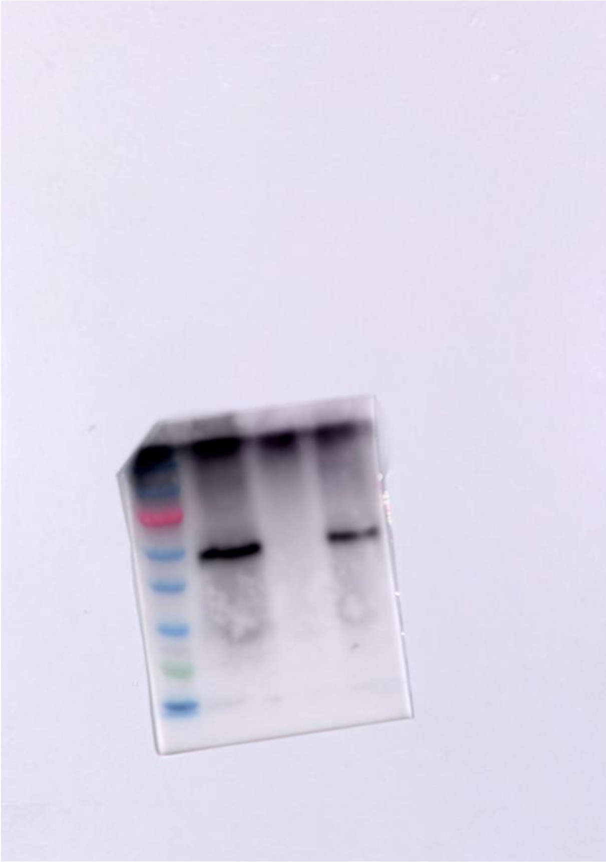

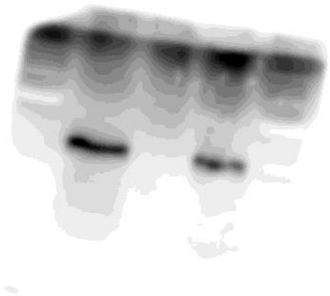

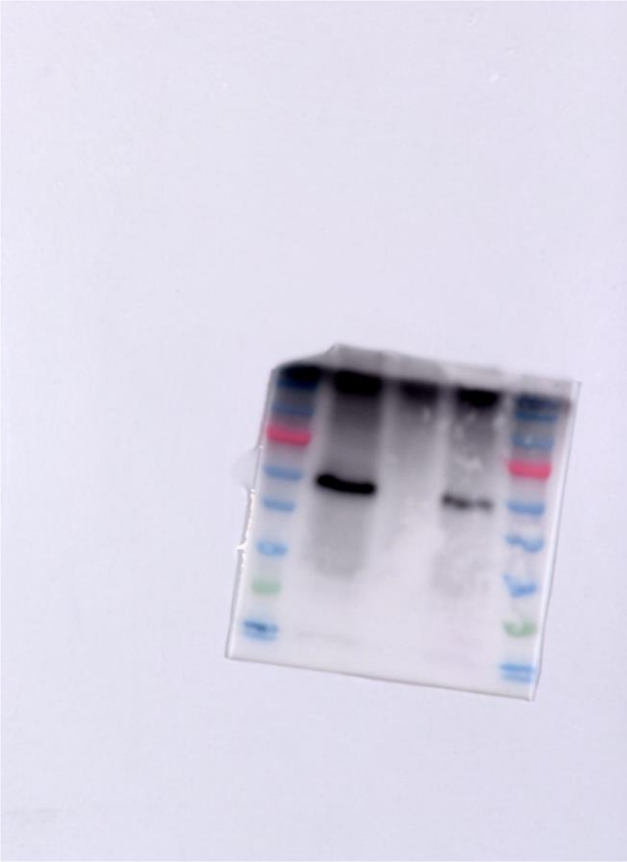

Right panel

BT549

MDA-MB-231

ANLN

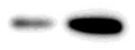

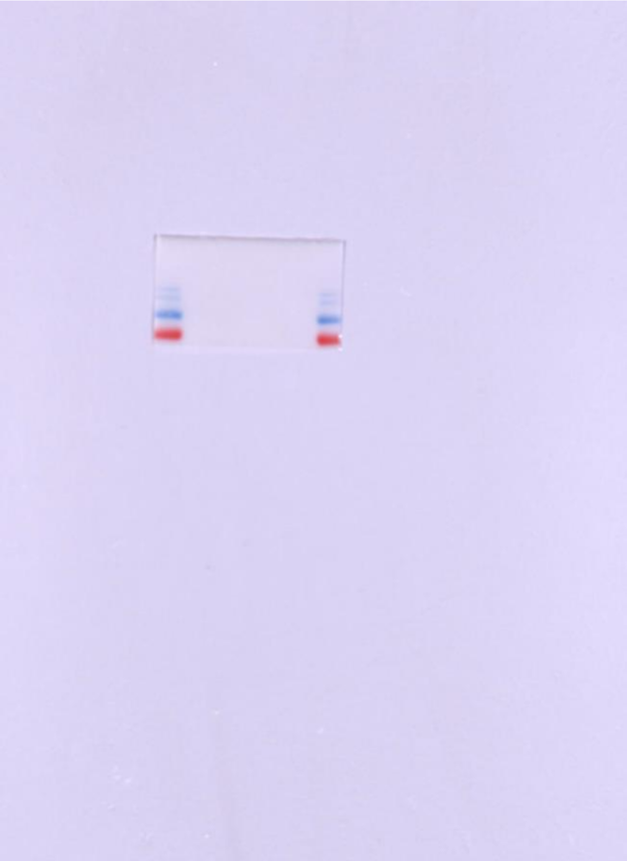

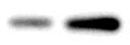

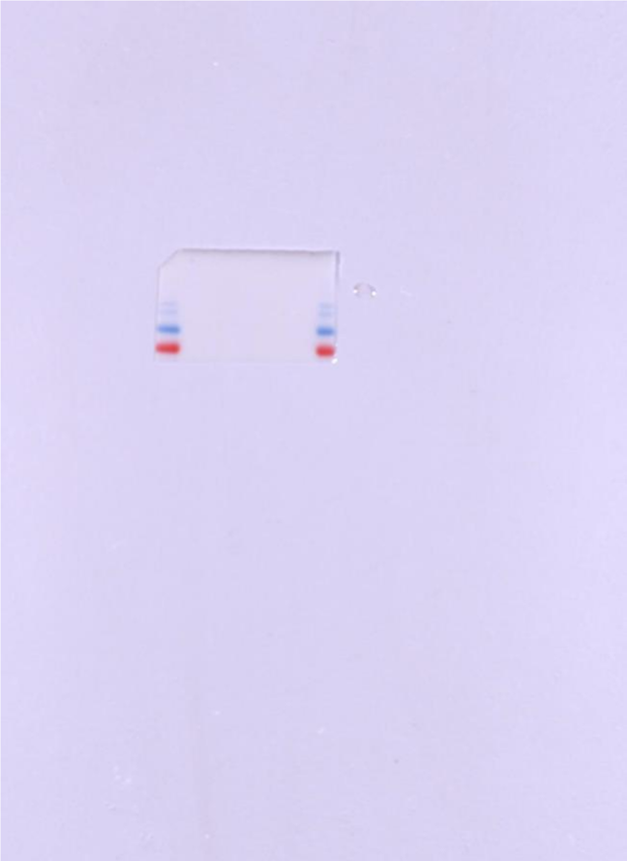

BT549

MDA-MB-231

CCNE1

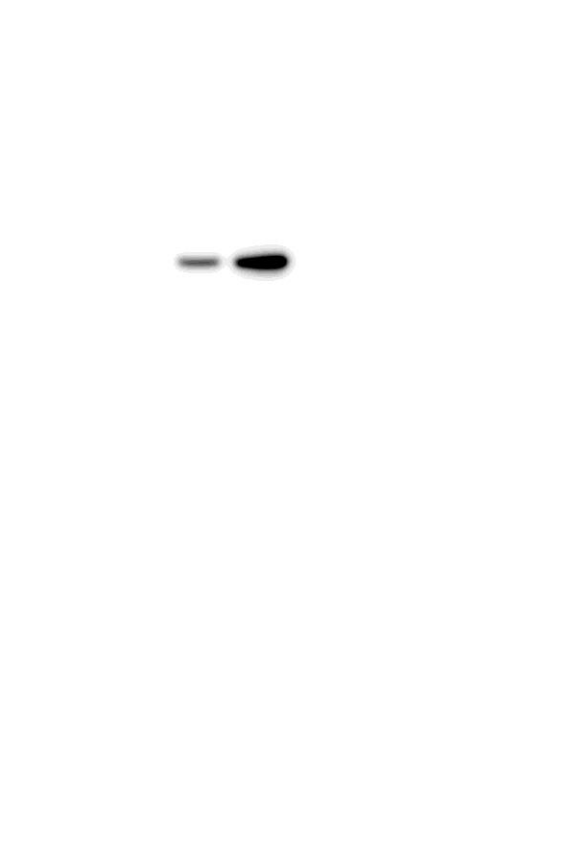

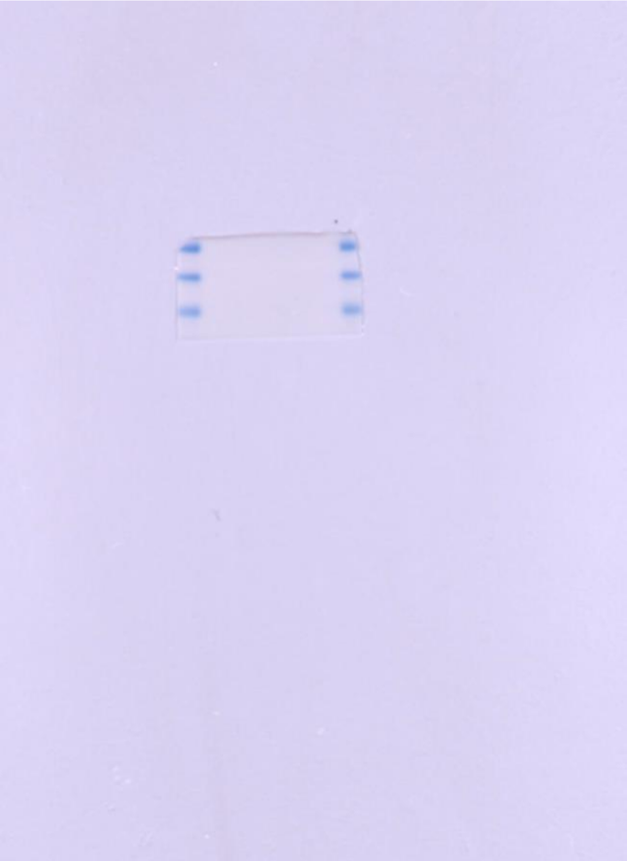

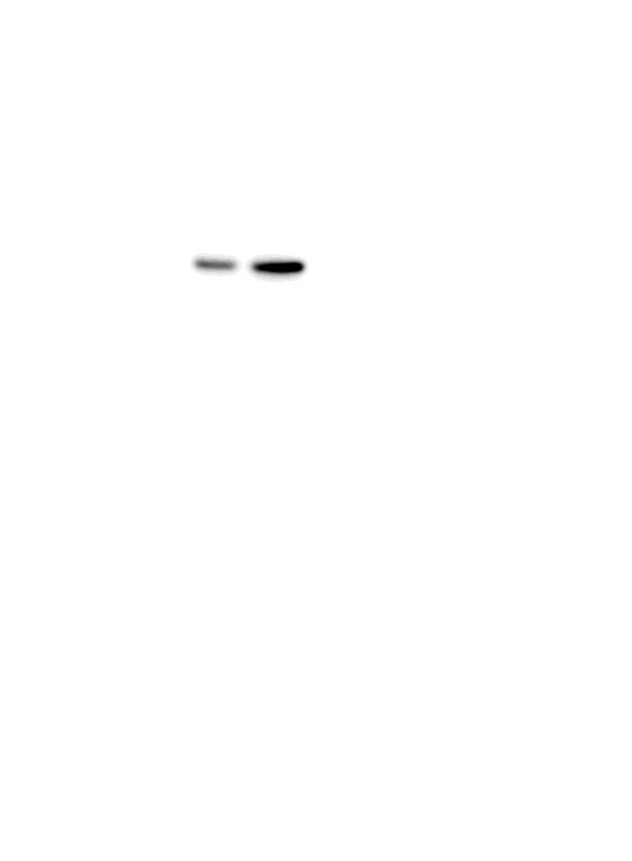

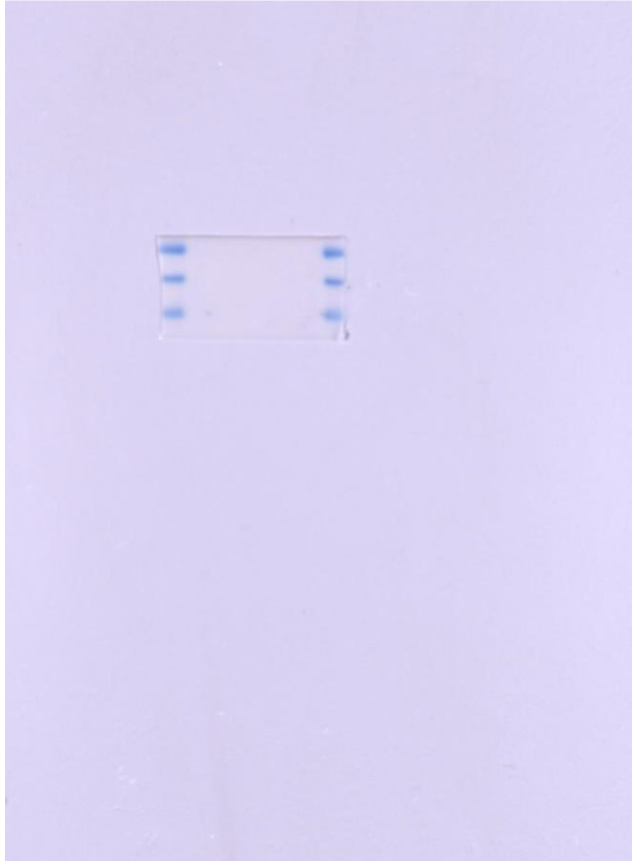

BT549

GAPDH

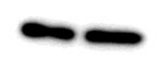

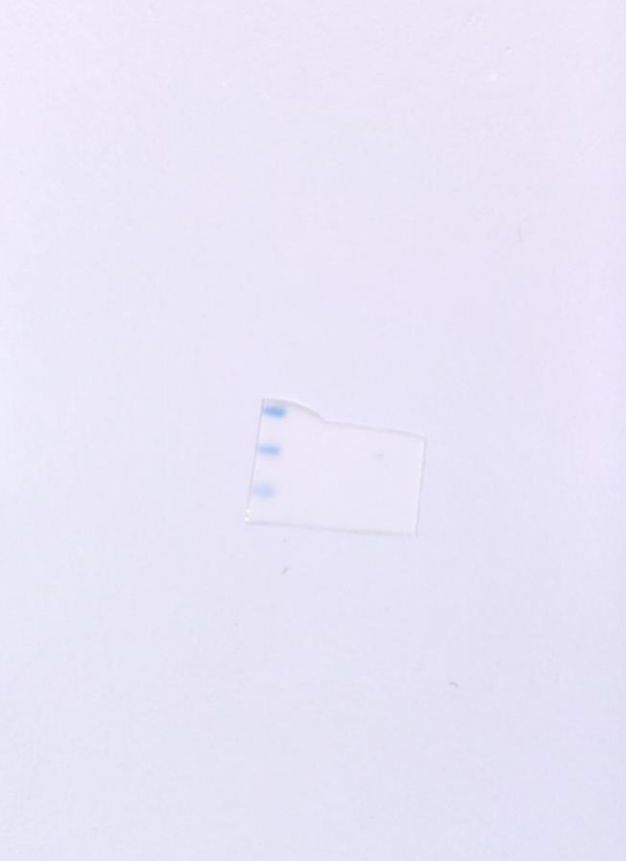

MDA-MB-231

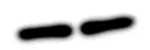

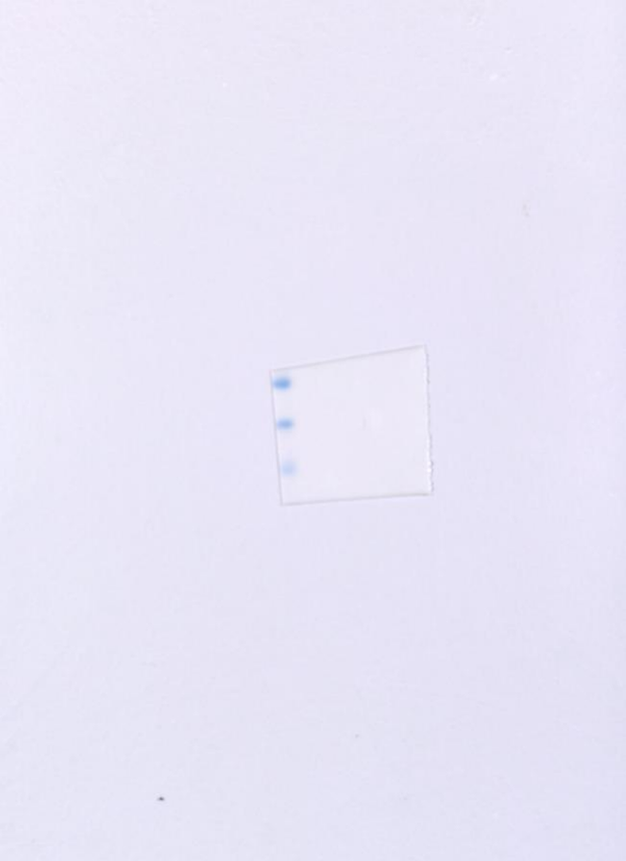

BT549

MDA-MB-231

ANLN

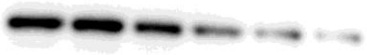

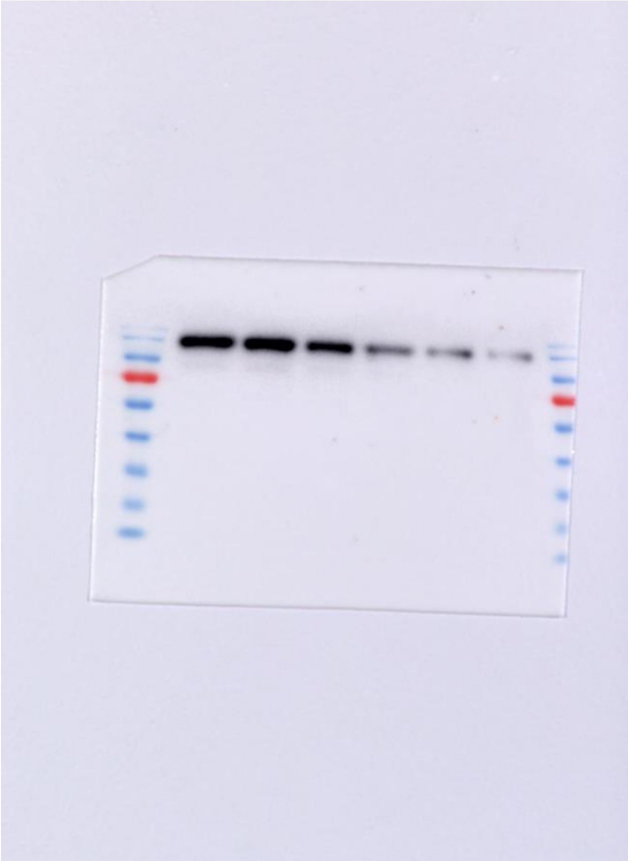

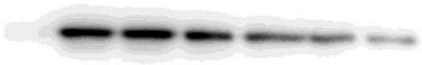

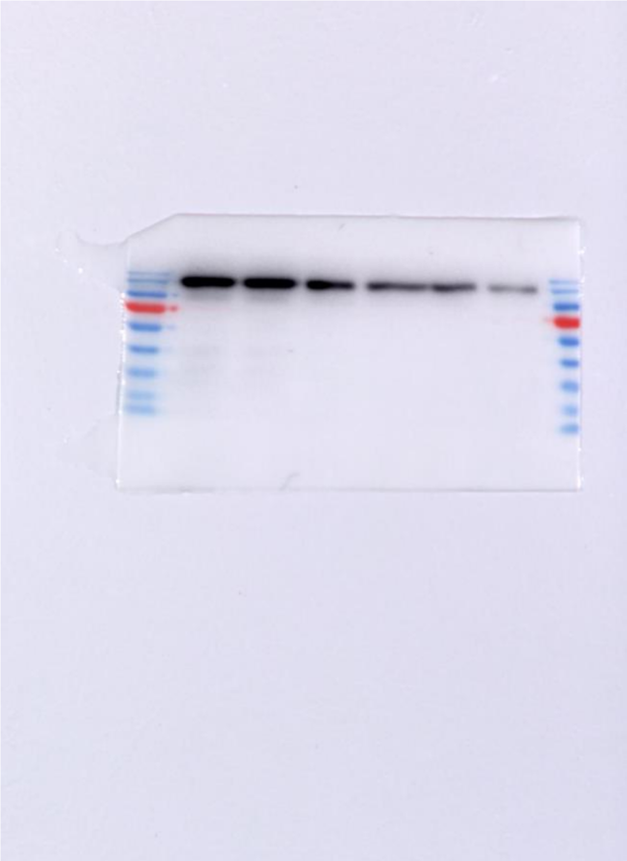

BT549

MDA-MB-231

GAPDH

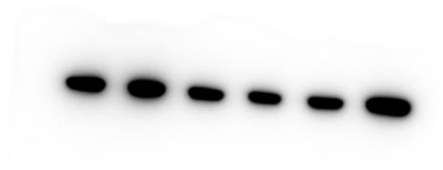

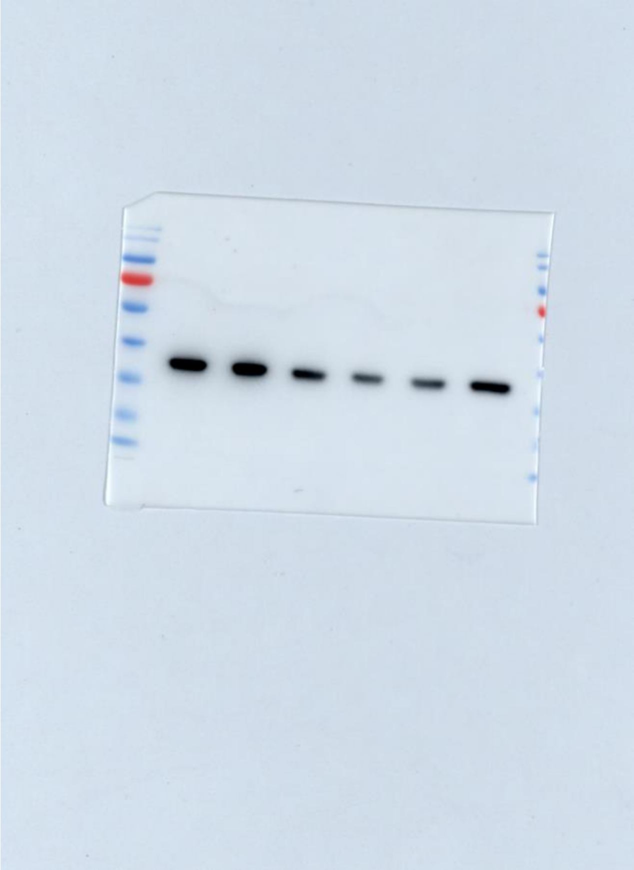

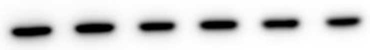

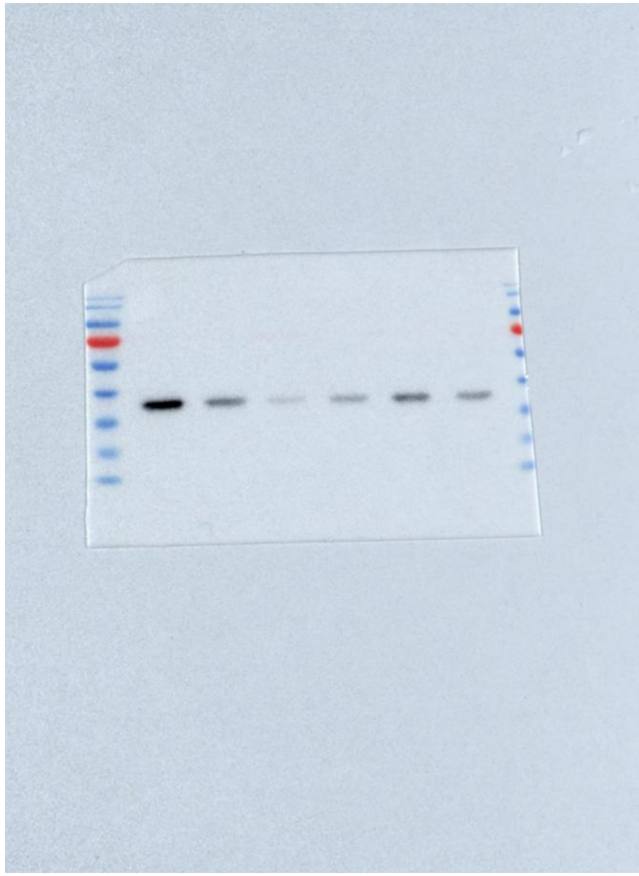

BT549

MDA-MB-231

ANLN

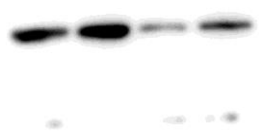

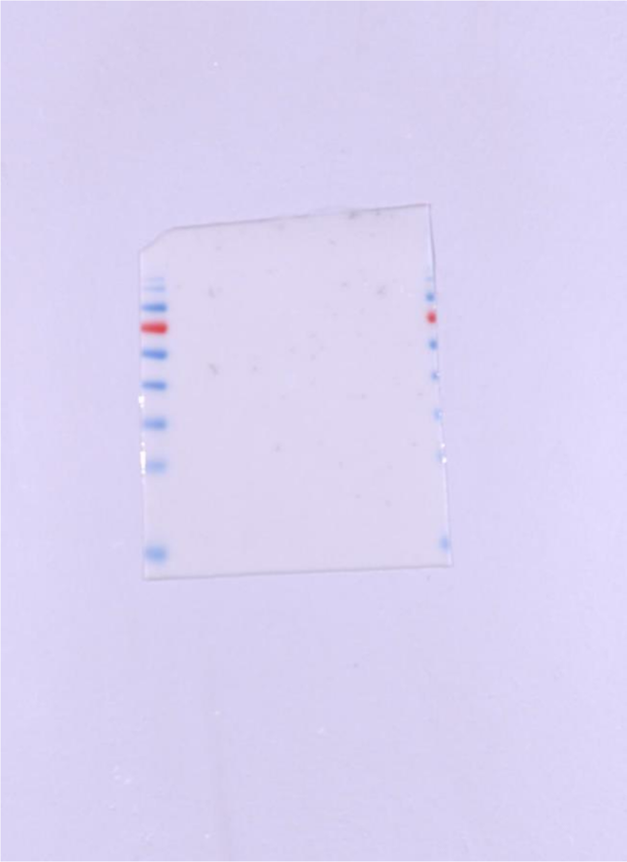

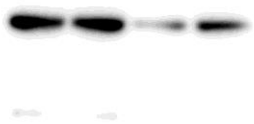

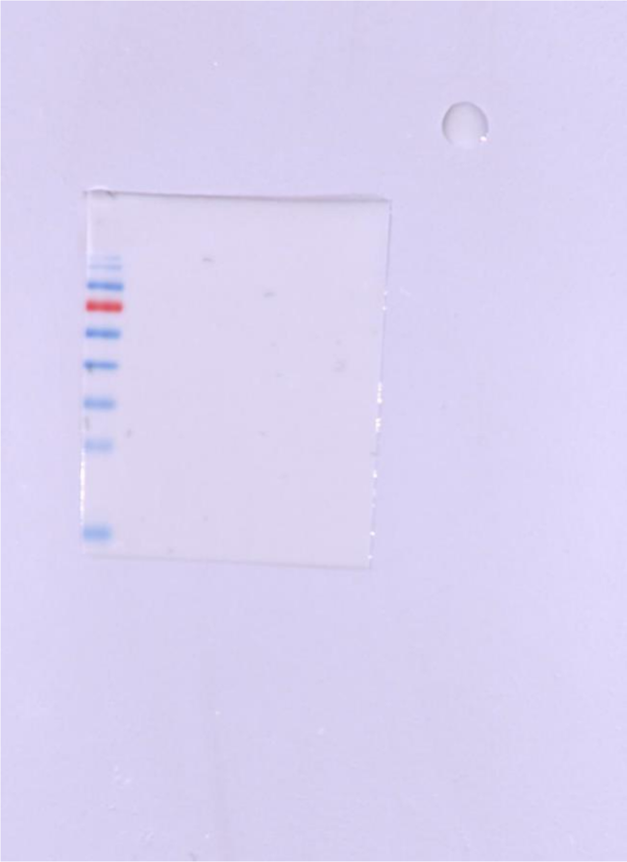

BT549

MDA-MB-231

GAPDH

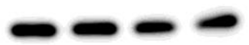

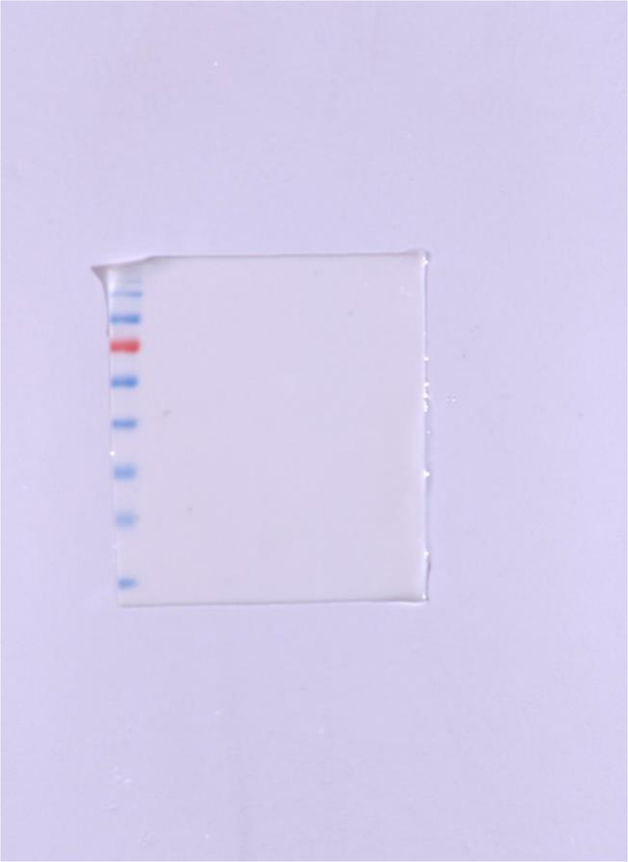

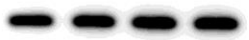

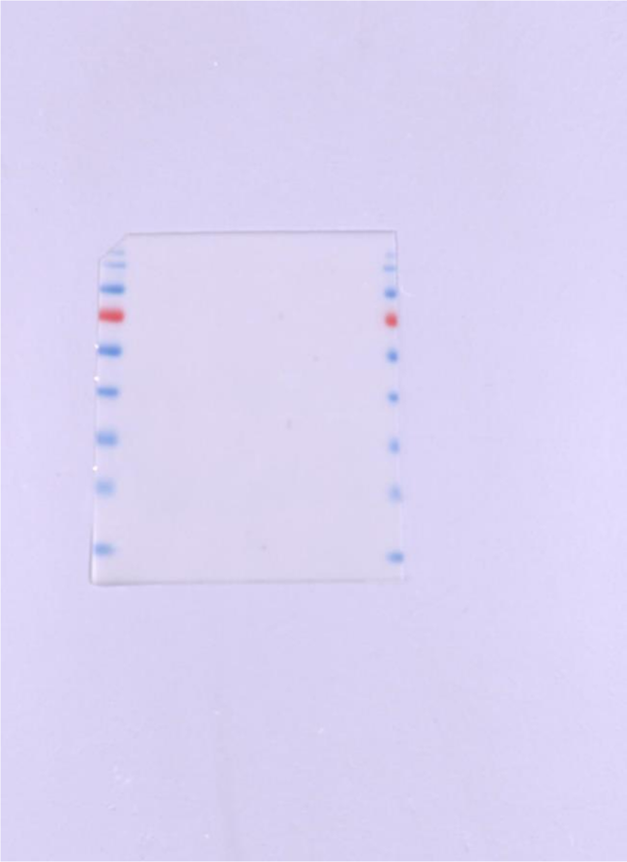

BT549

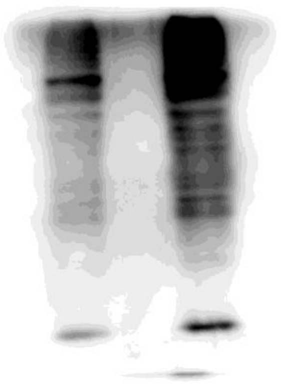

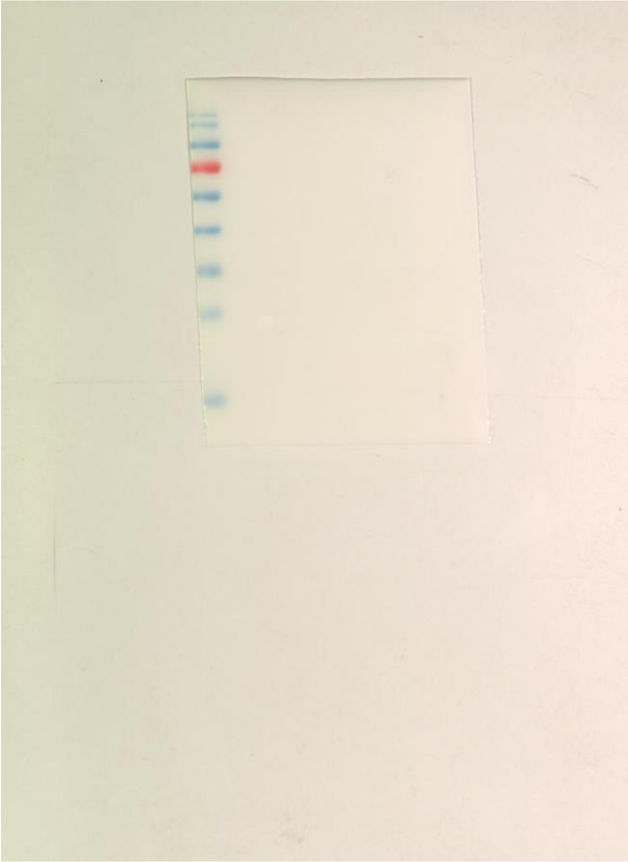

MDA-MB-231

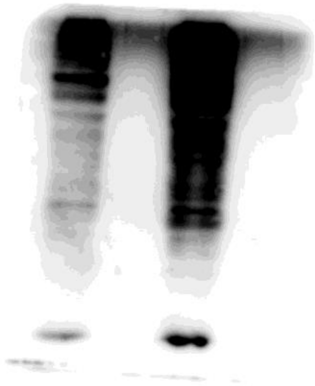

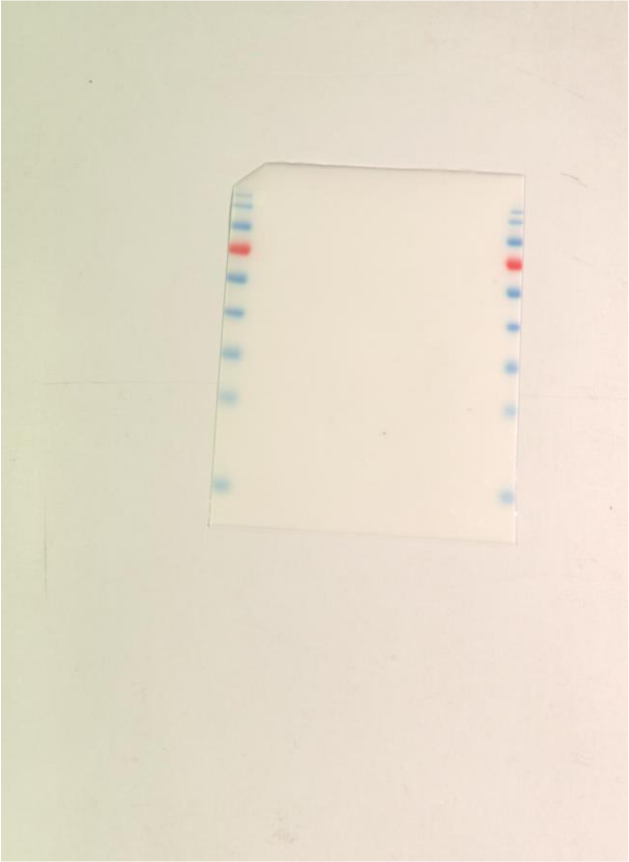

BT549

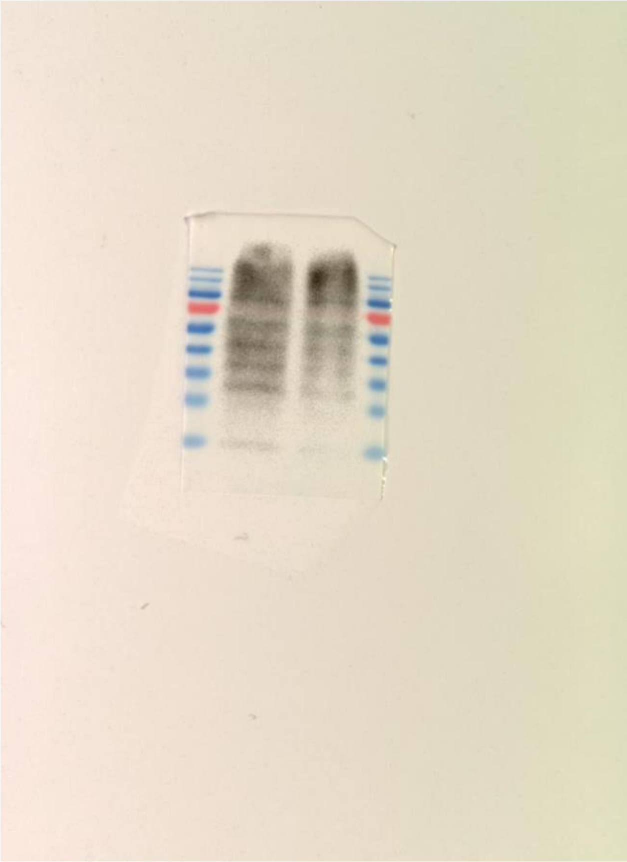

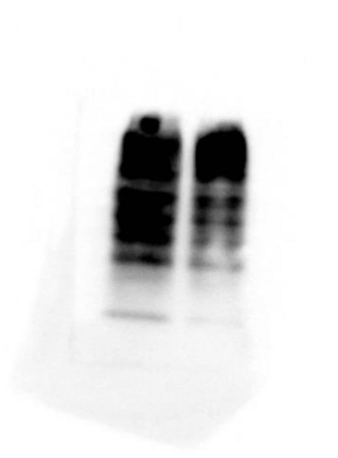

MDA-MB-231

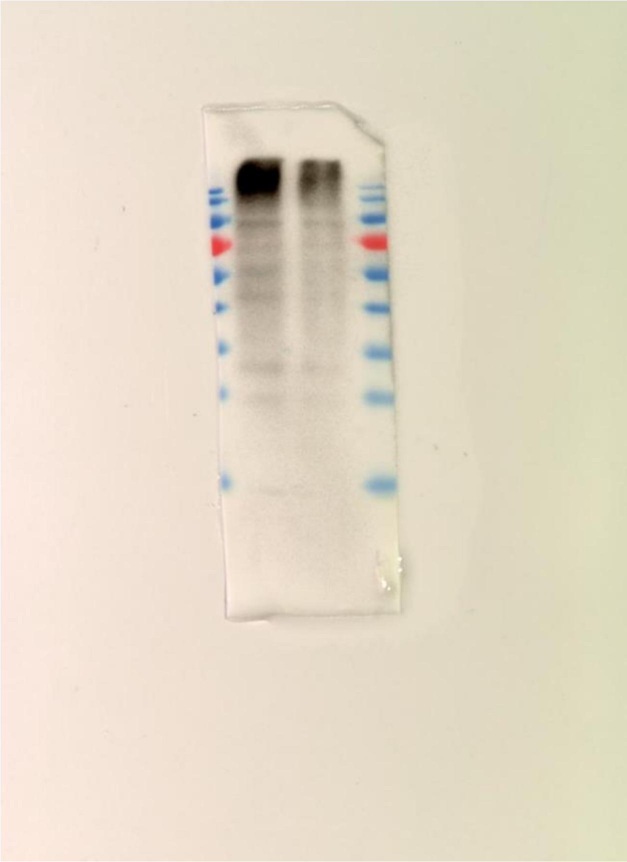

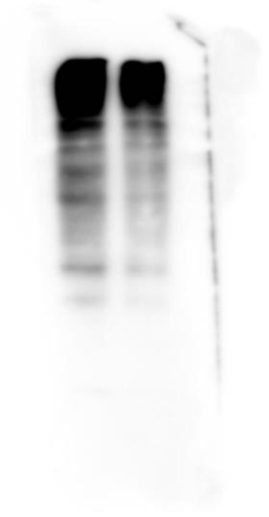

BT549

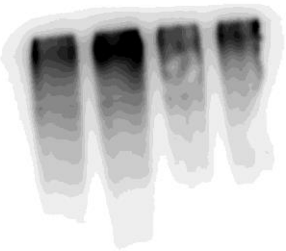

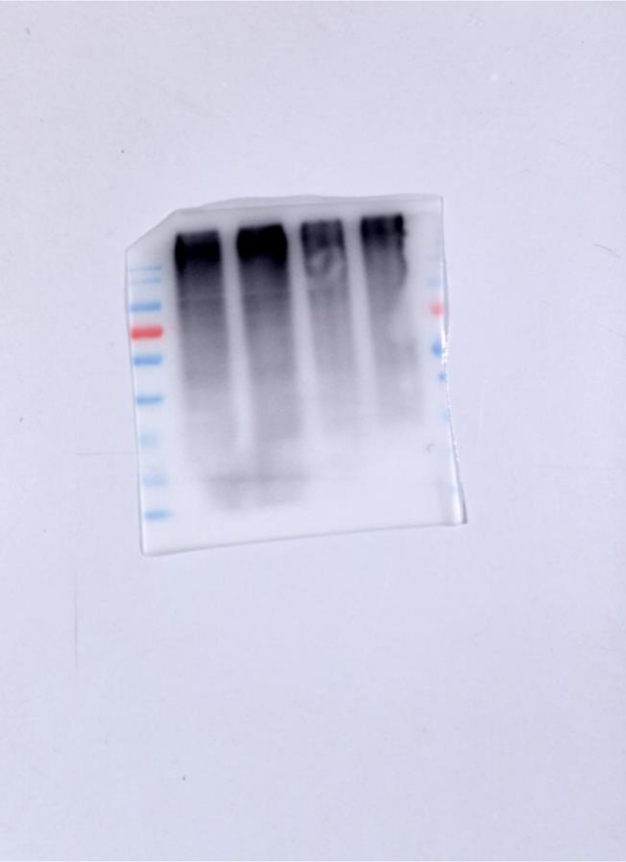

MDA-MB-231

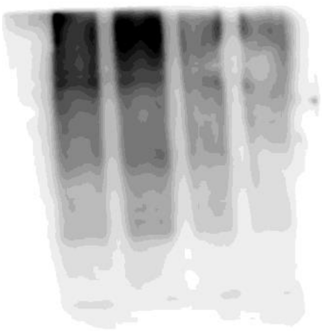

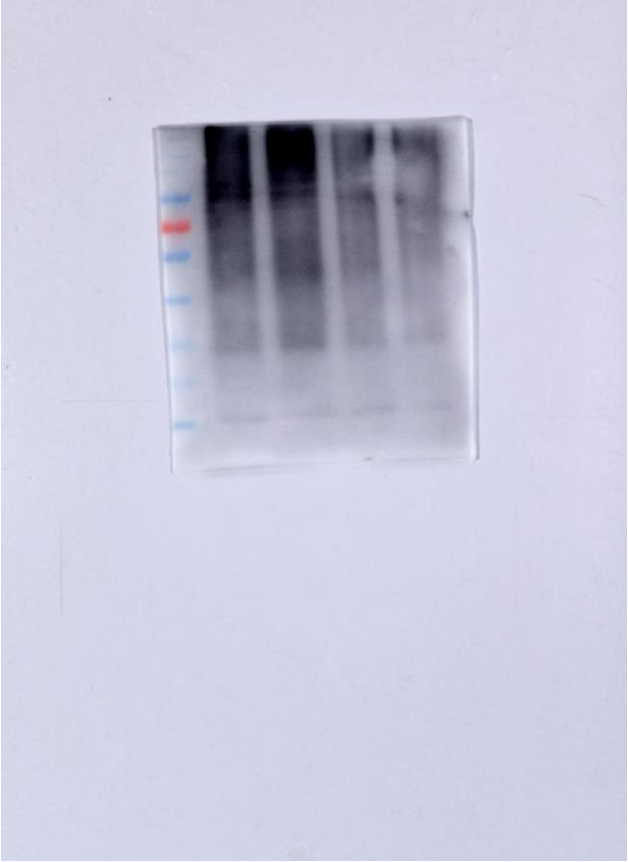

ANLN-FLAG

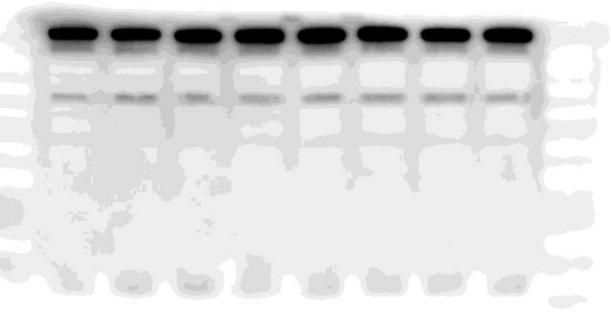

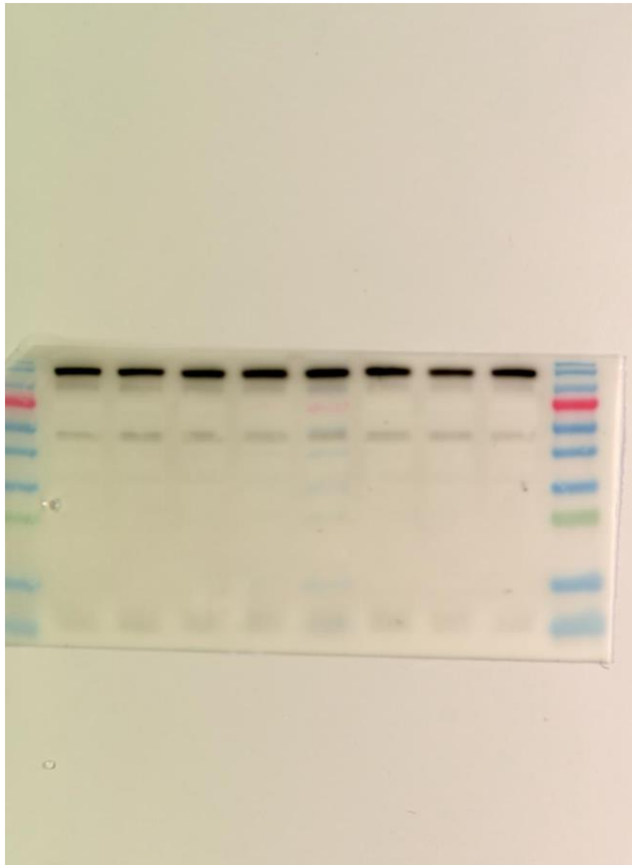

INPUT ANLN

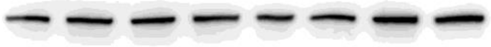

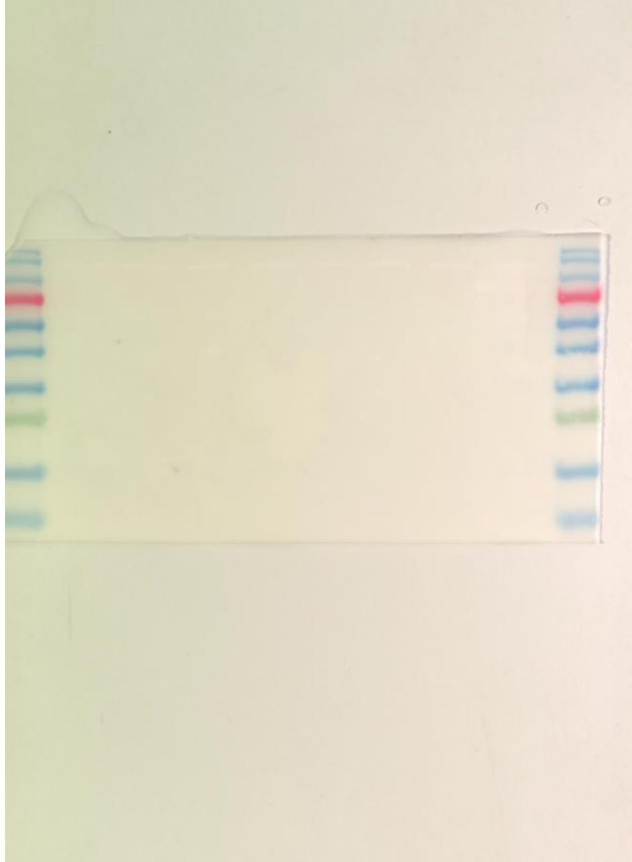

INPUT CCNE1

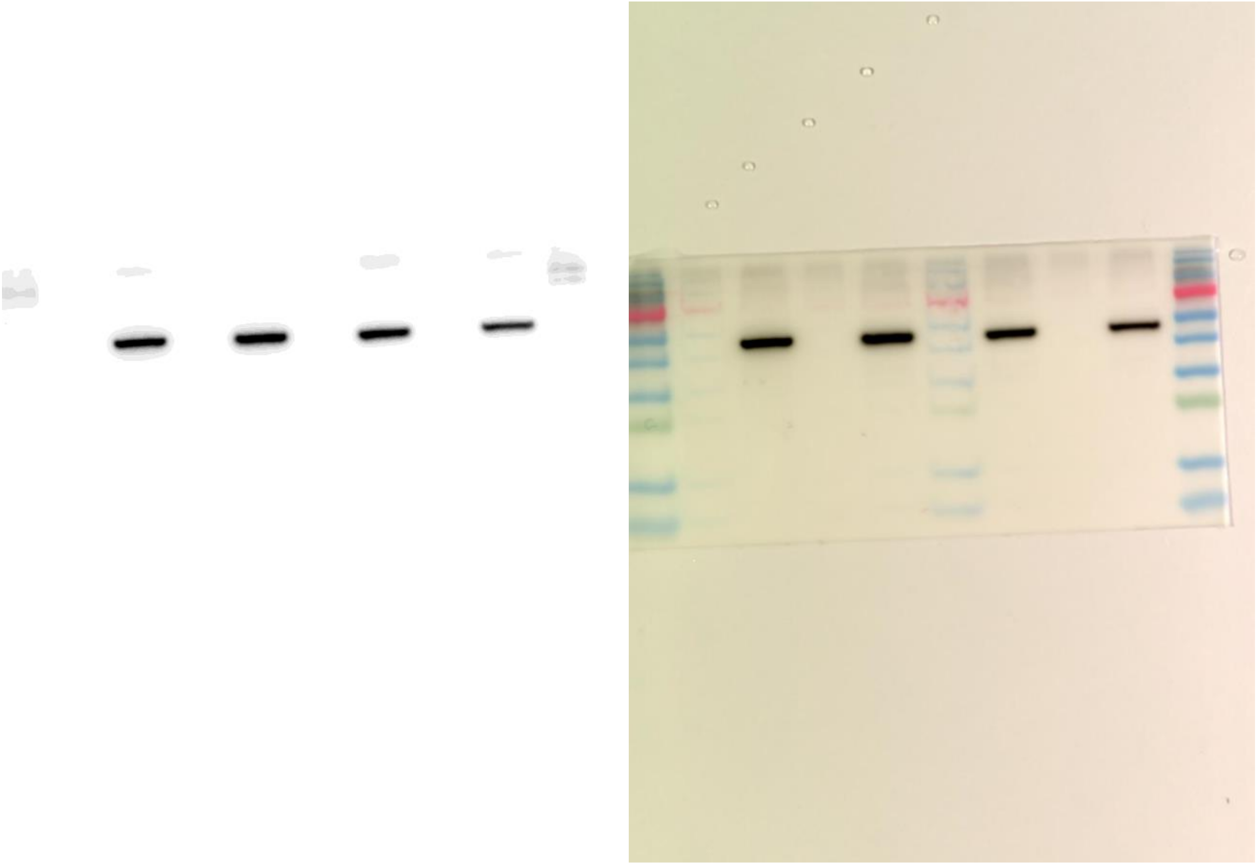

INPUT UB

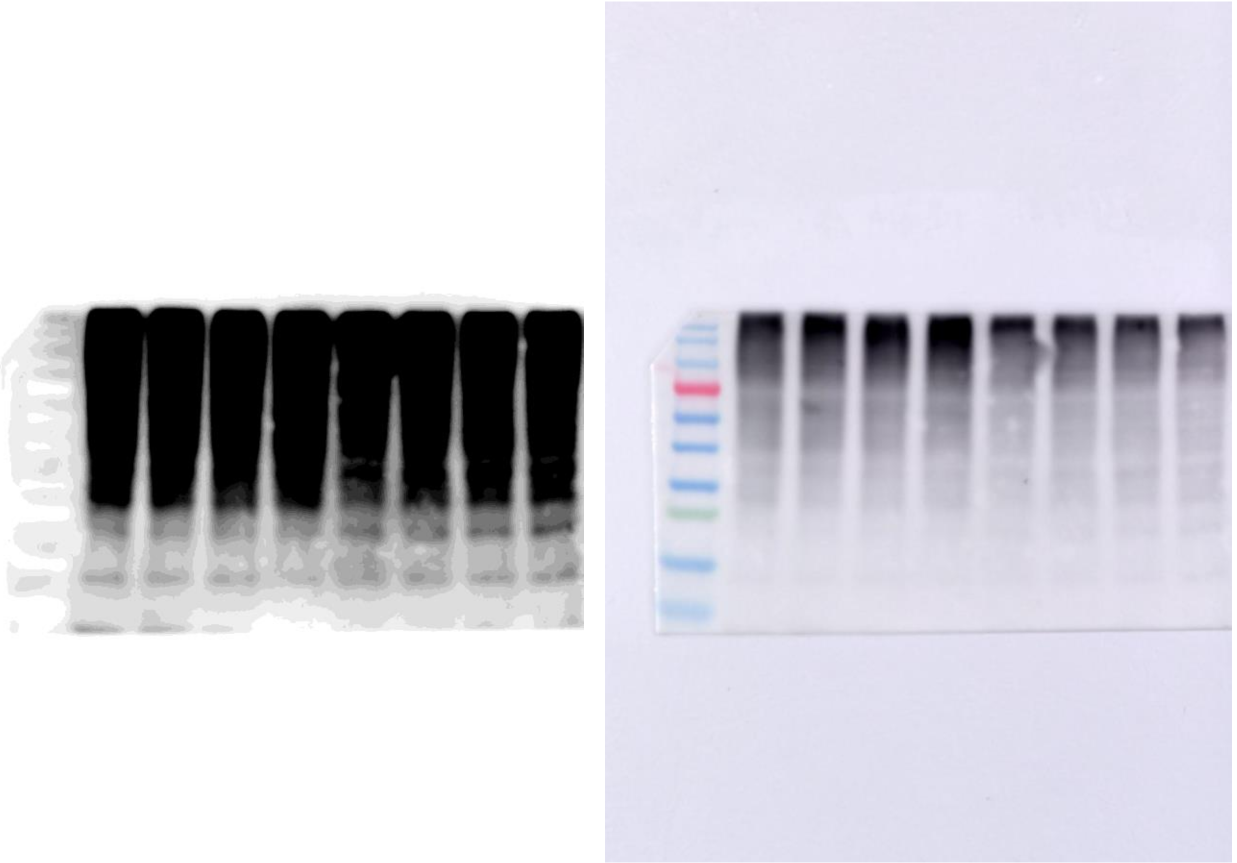

Ubiquitin

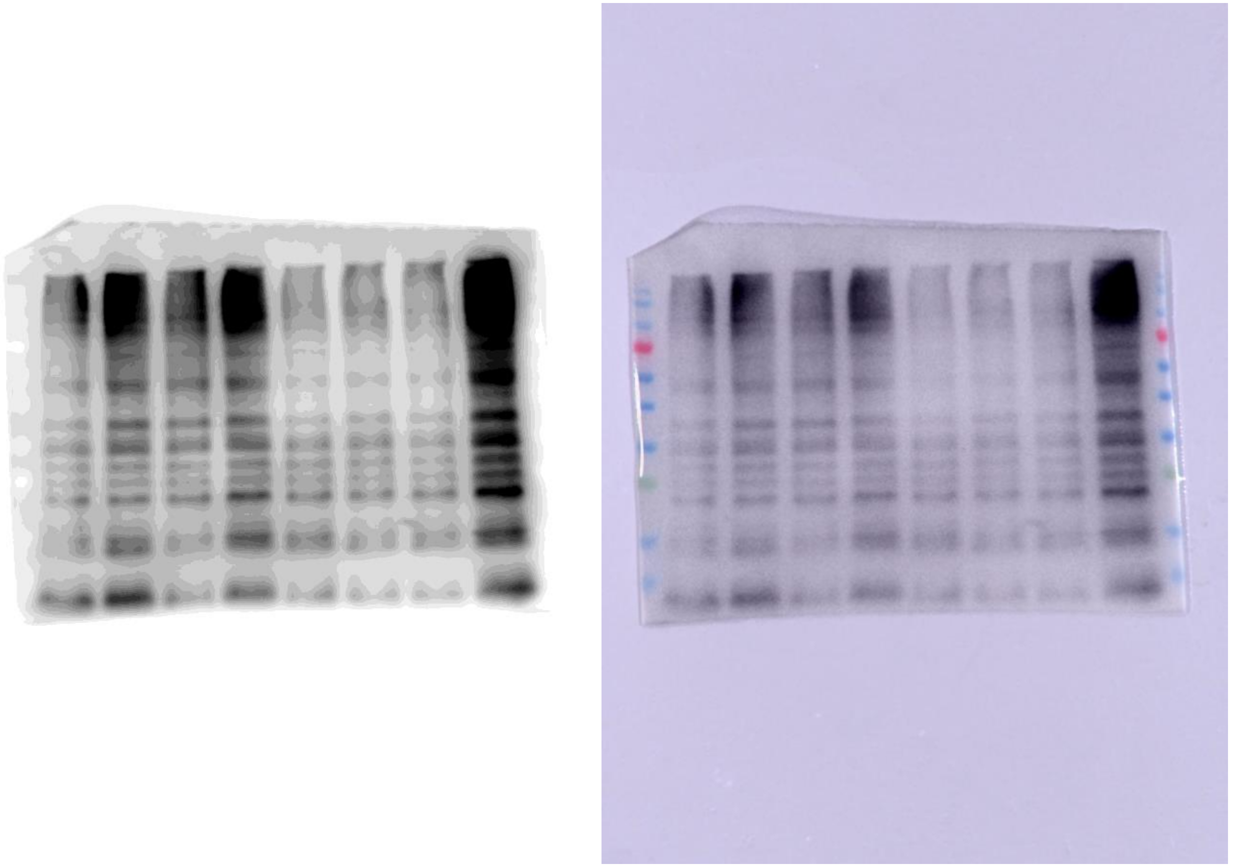

β-Actin

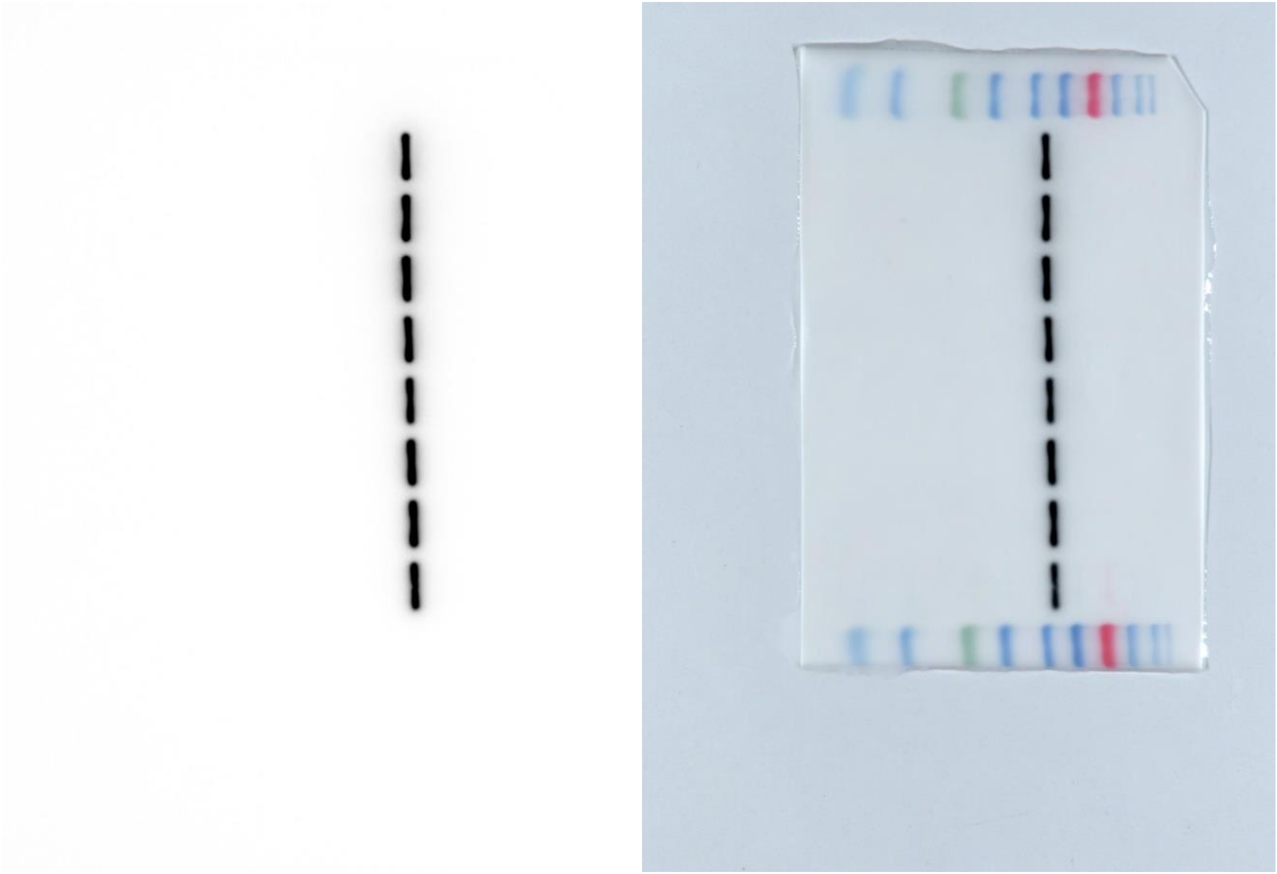

BT-549

MDA-MB-231

ANLN-3xflag

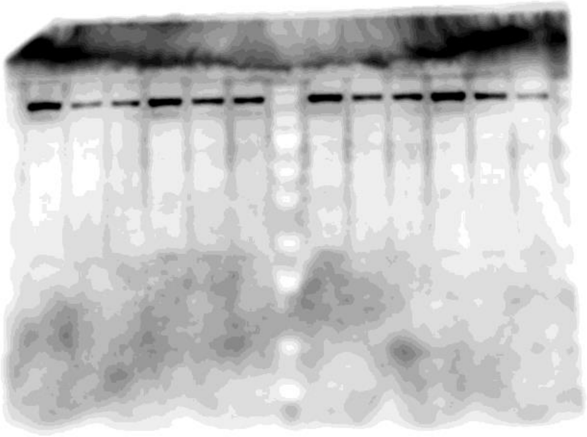

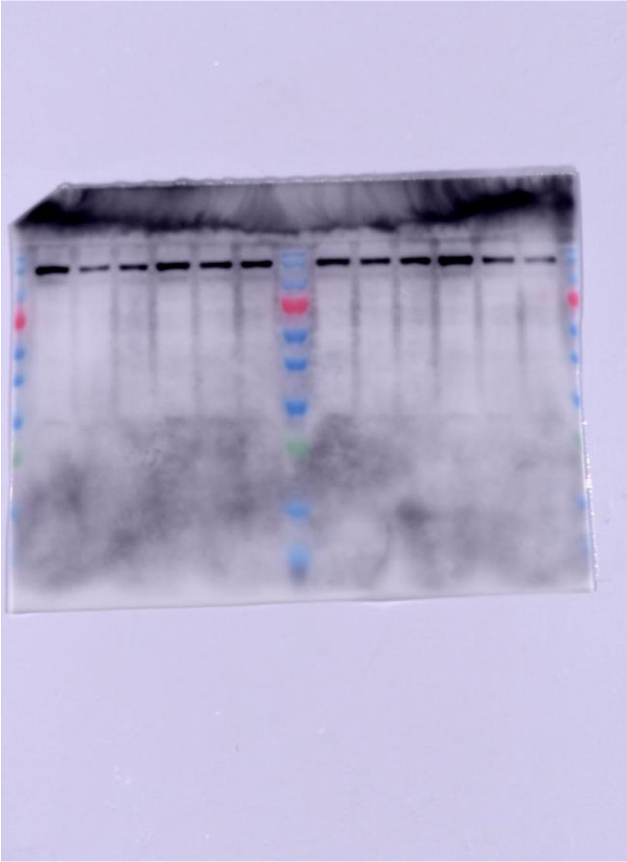

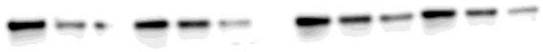

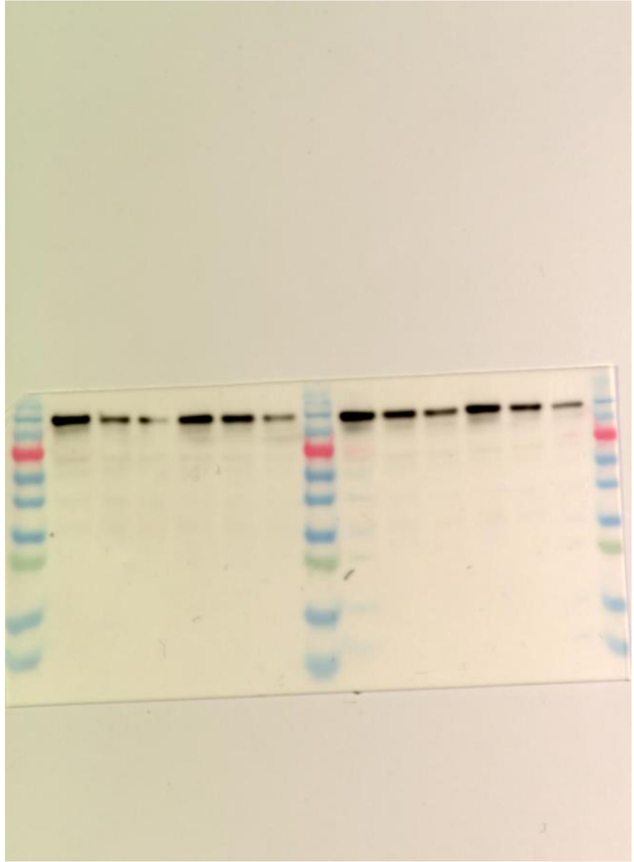

BT-549

MDA-MB-231

β-Actin

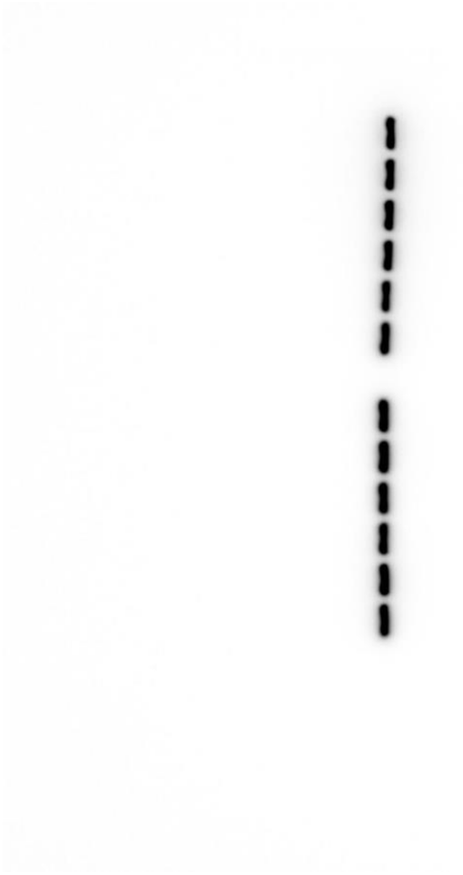

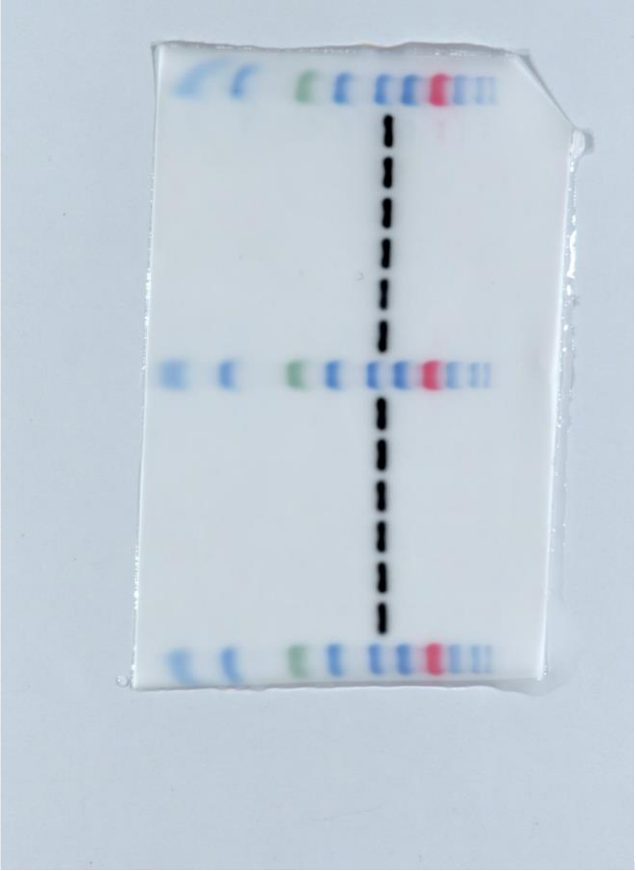

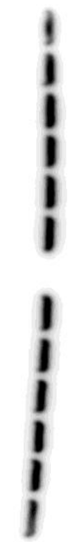

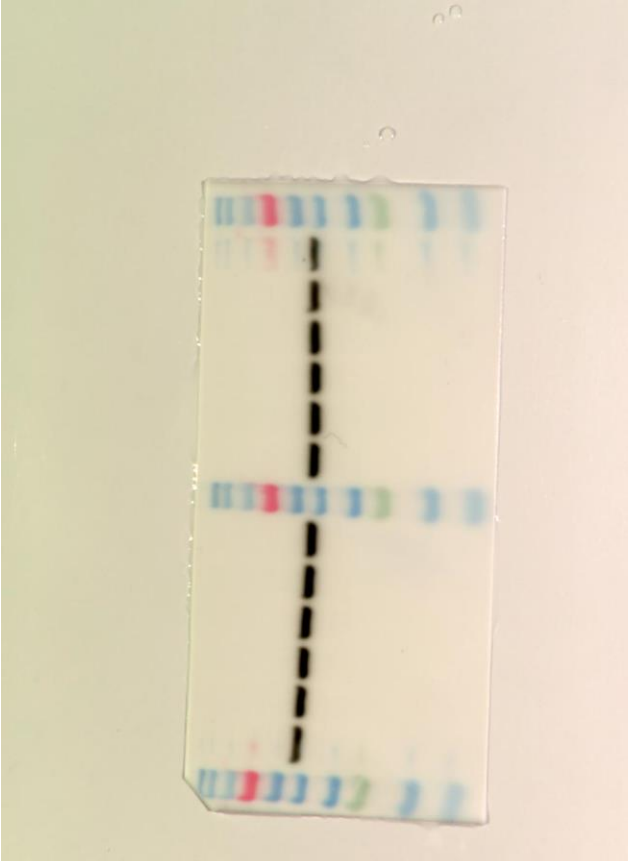

BT-549

MDA-MB-231

CD44

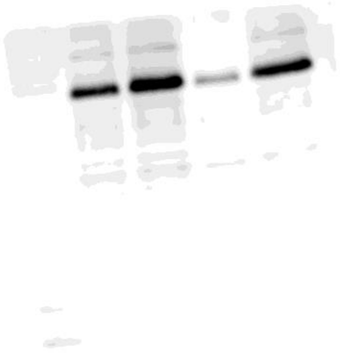

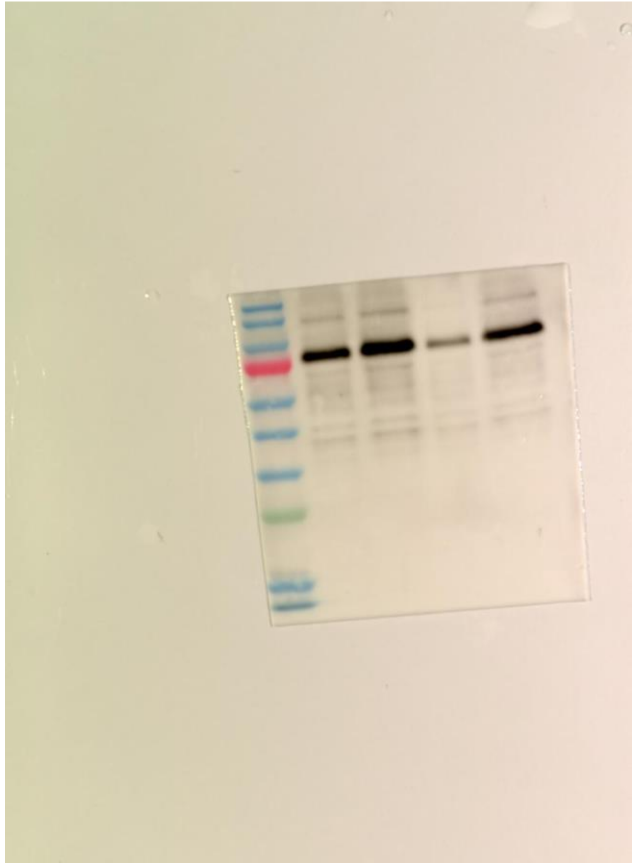

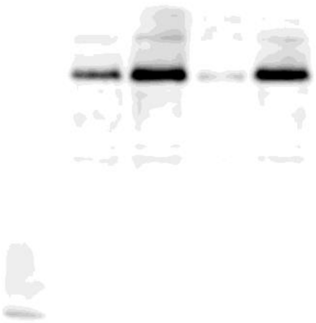

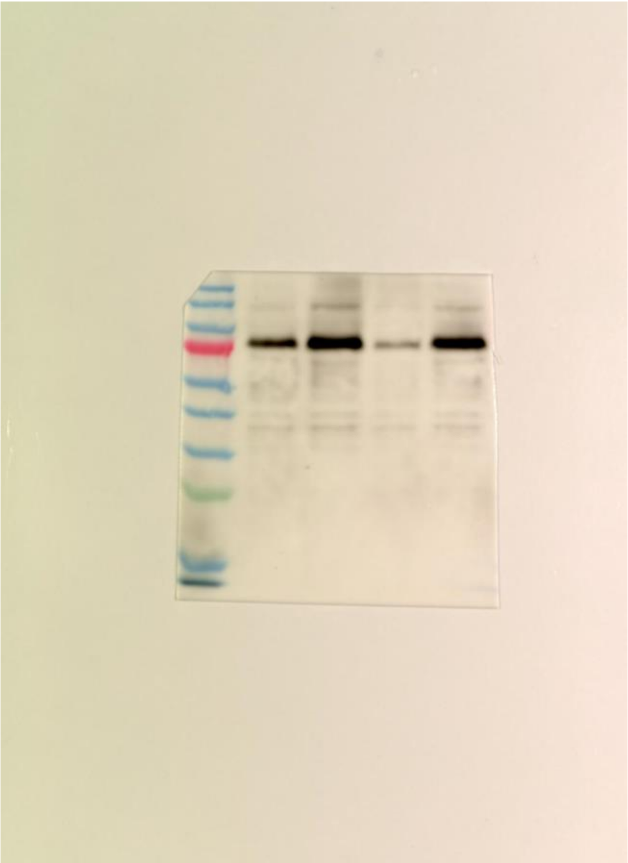

BT-549

CD133

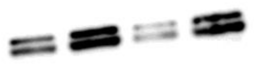

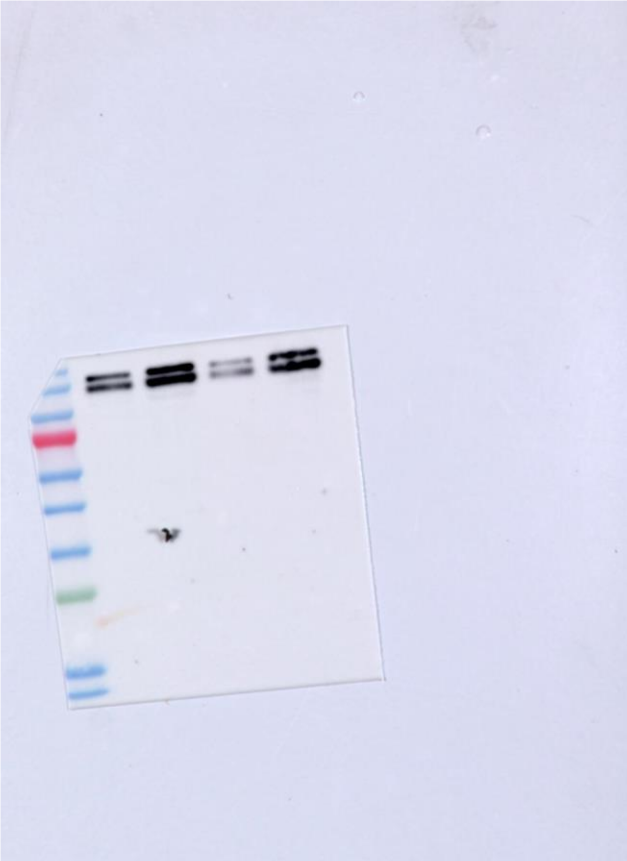

MDA-MB-231

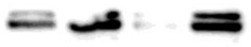

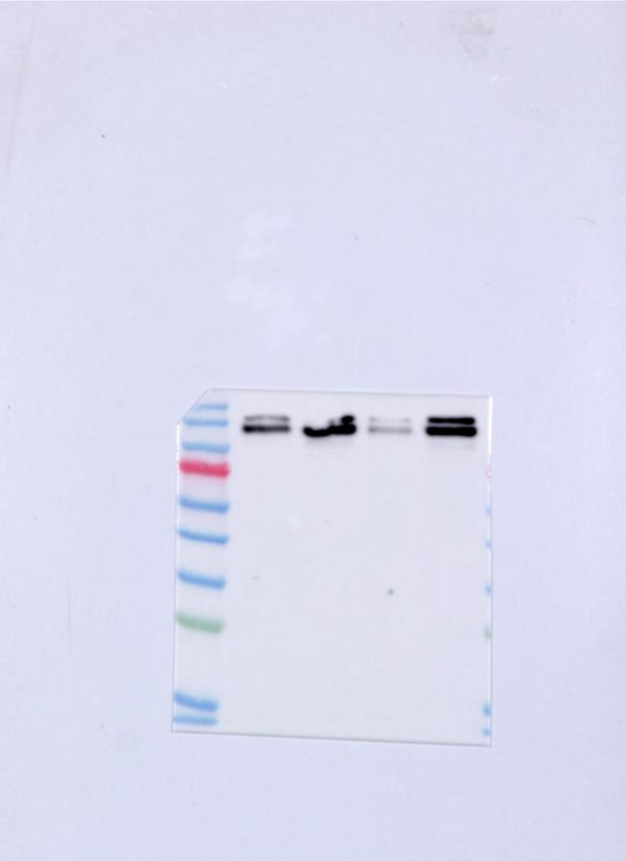

BT-549

SOX2

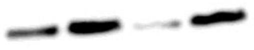

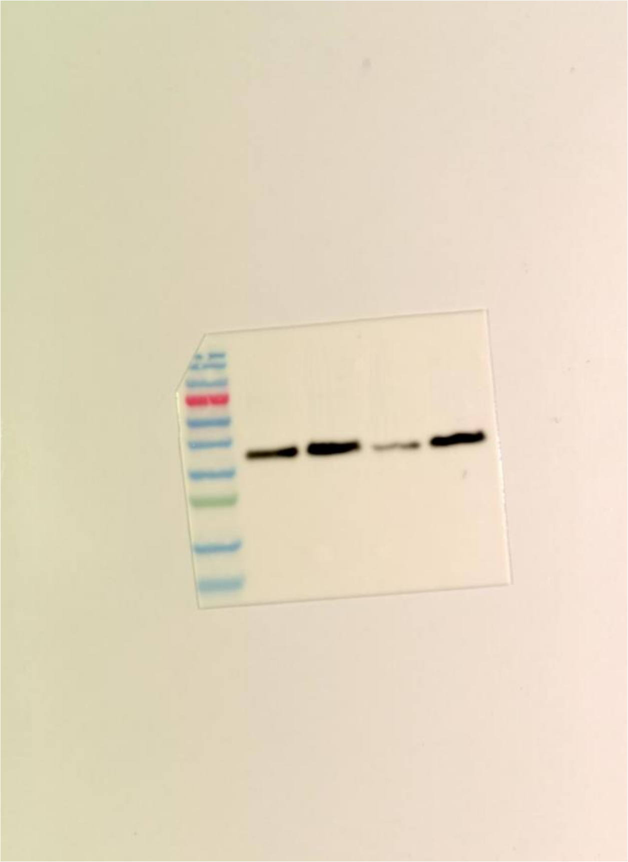

MDA-MB-231

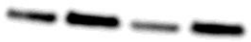

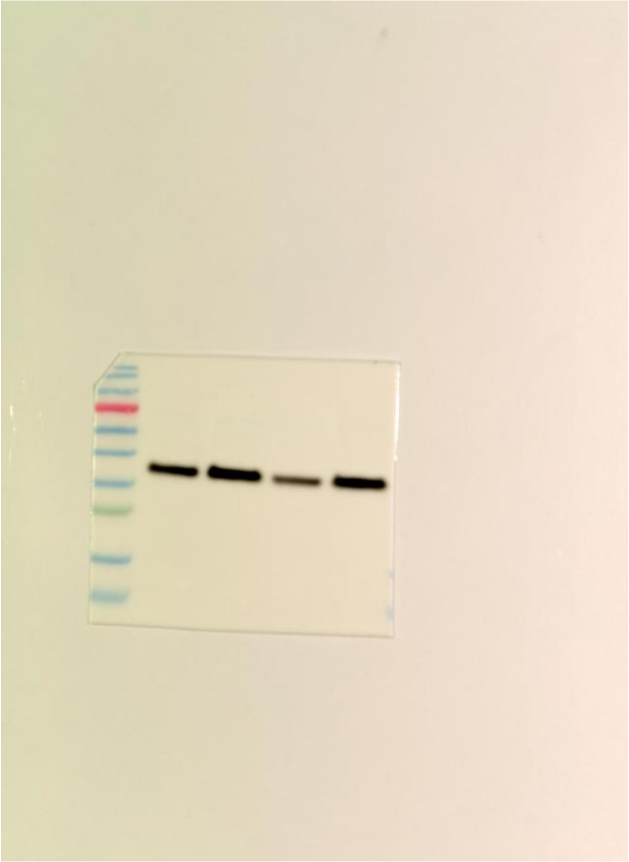

BT-549

MDA-MB-231

GSK3αβ

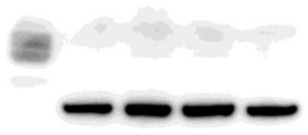

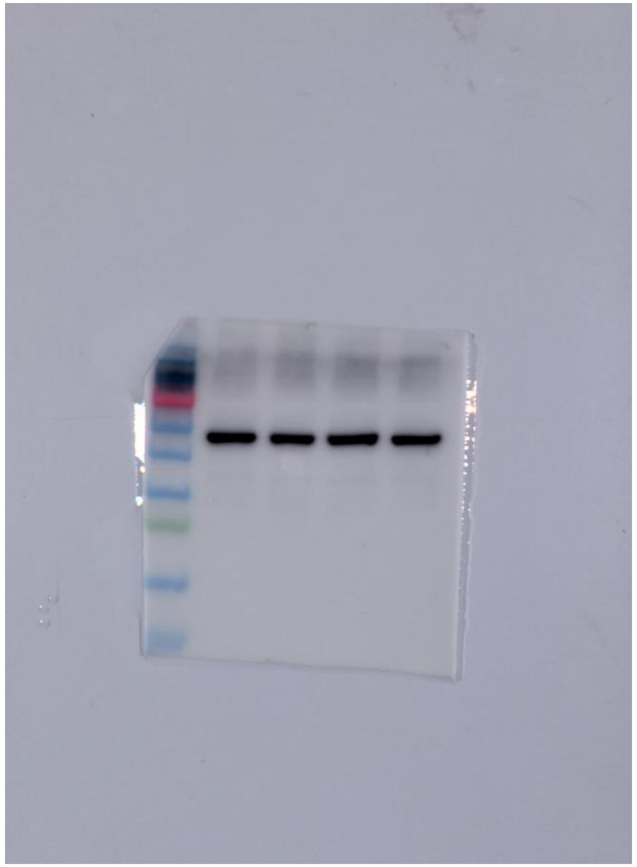

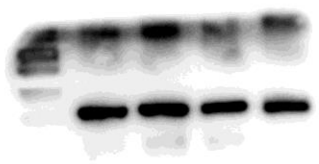

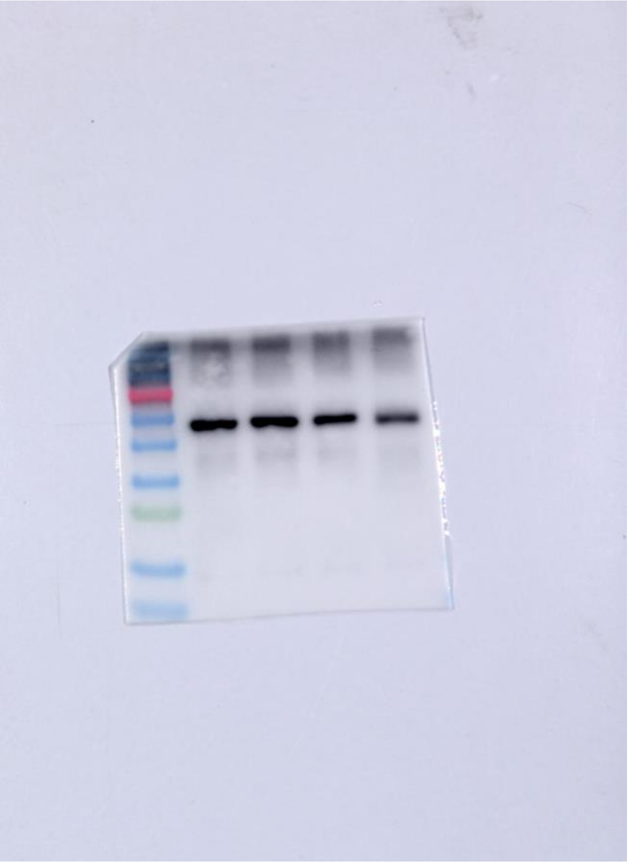

BT-549

MDA-MB-231

P-GSK3αβ

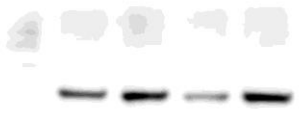

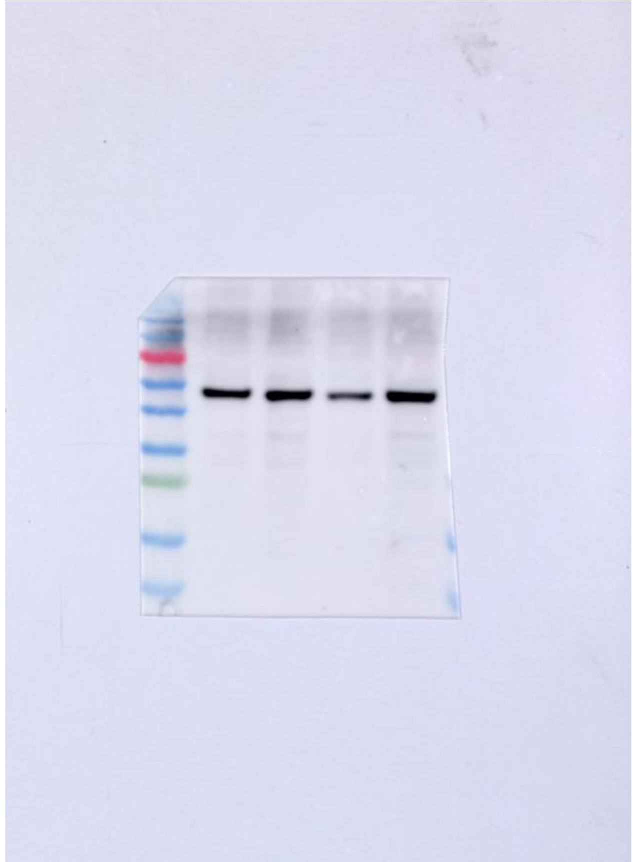

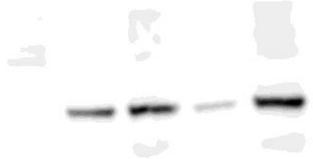

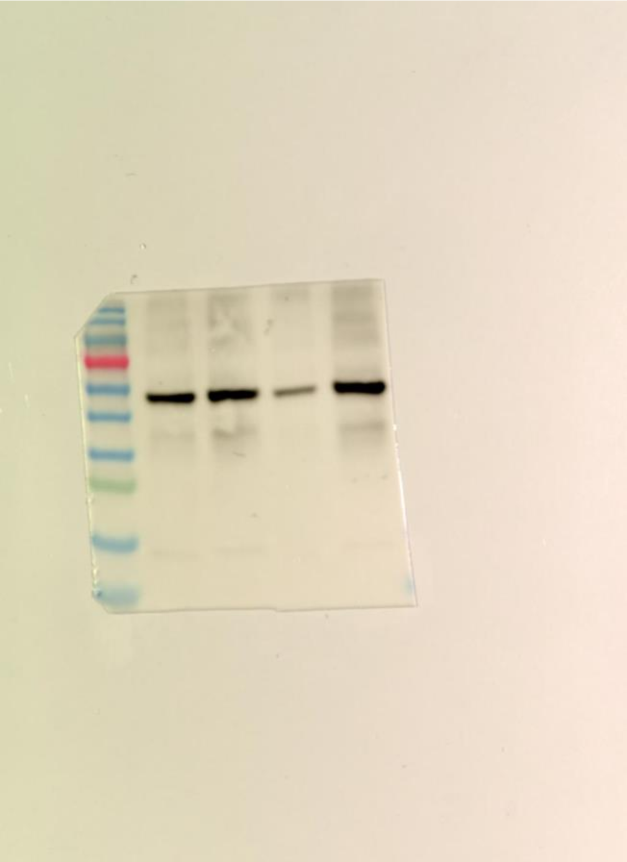

BT-549

β-Catenin

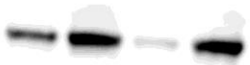

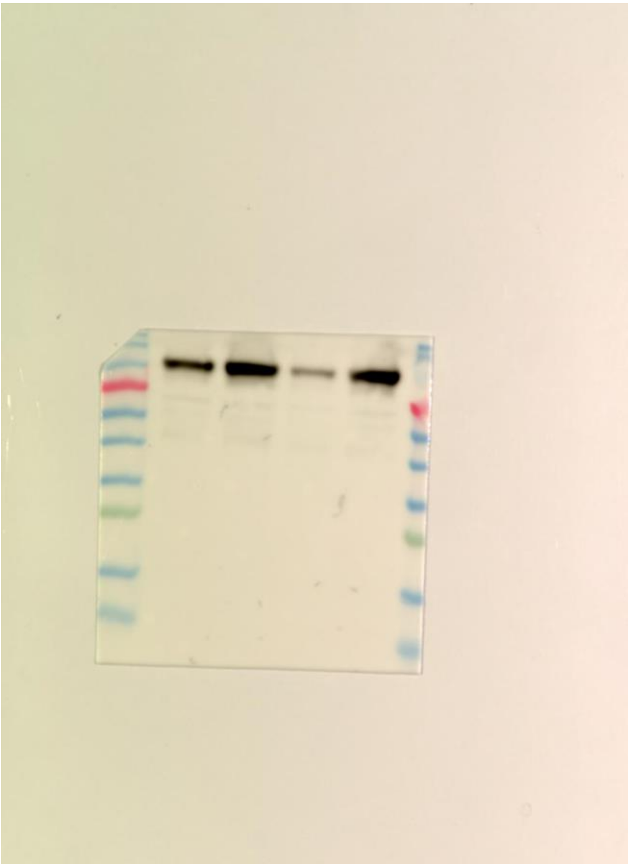

MDA-MB-231

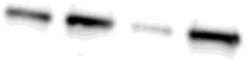

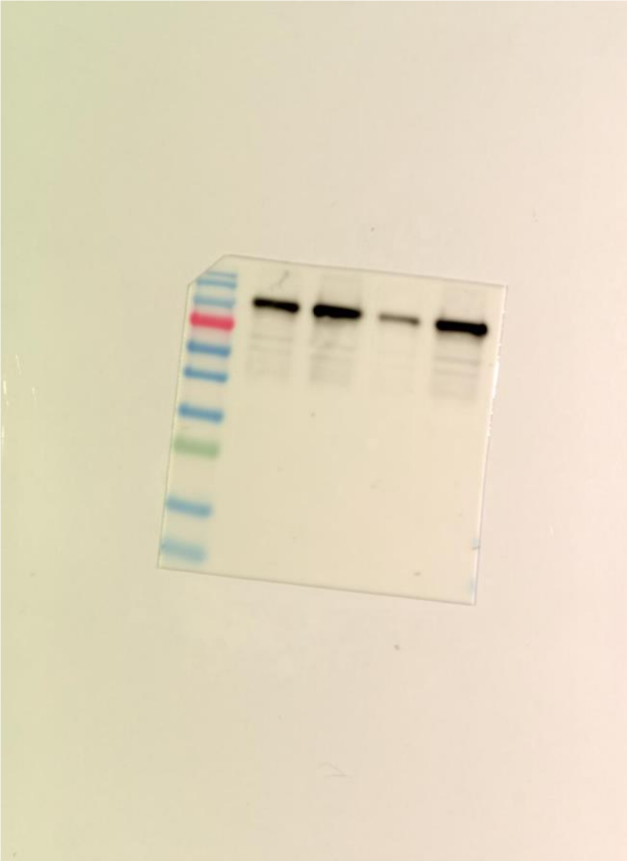

BT-549

MDA-MB-231

β-Actin

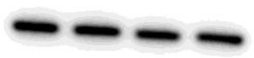

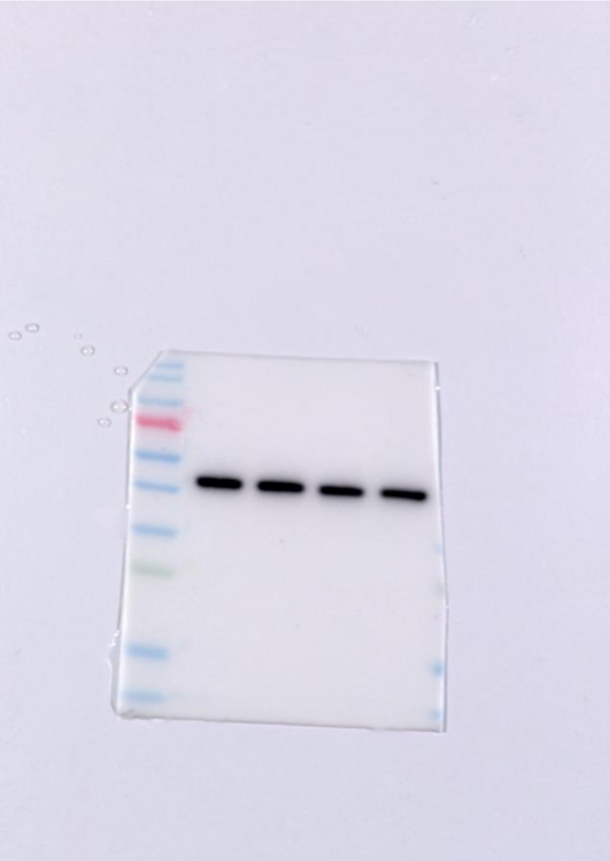

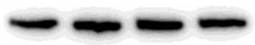

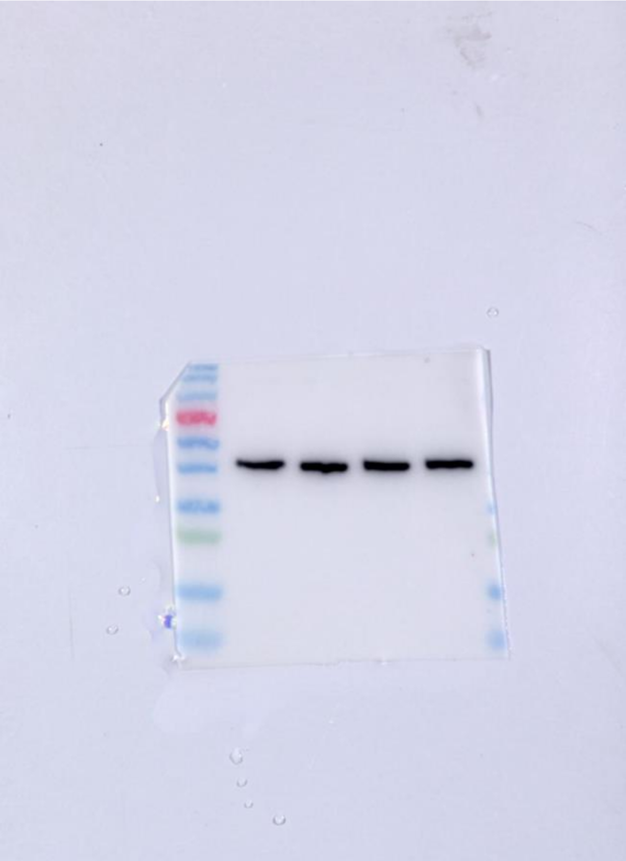

BT-549 ANLN

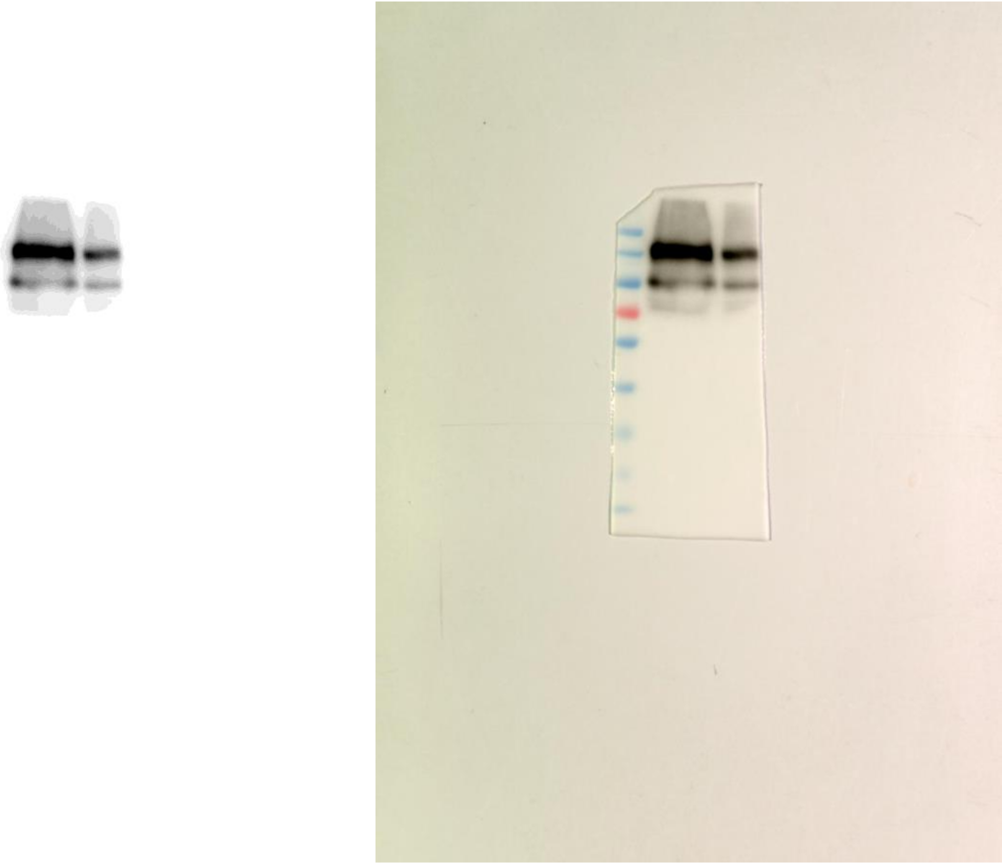

BT-549 GAPDH

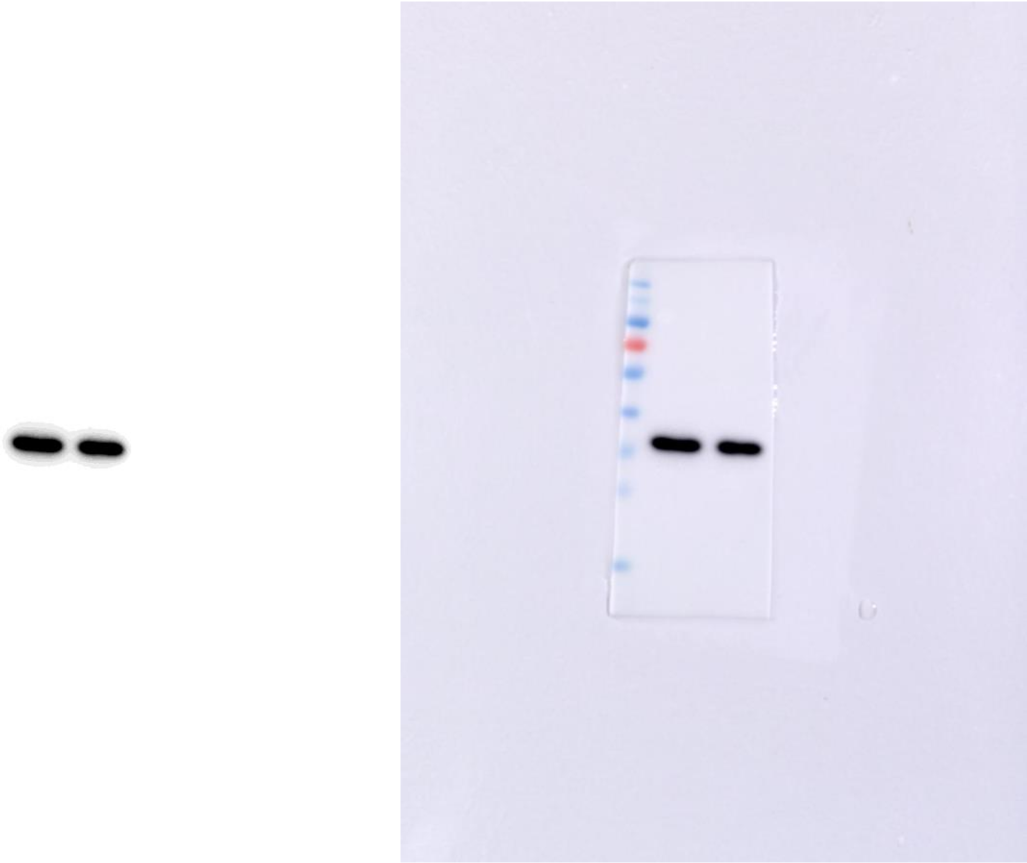

MDA-MB-231 ANLN

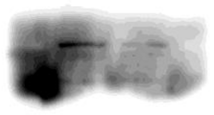

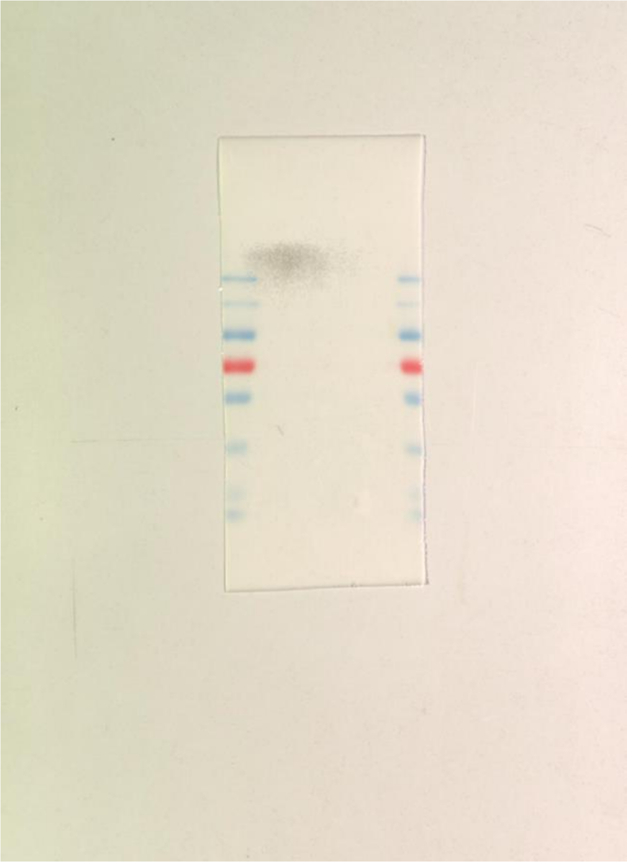

MDA-MB-231 GAPDH

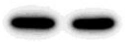

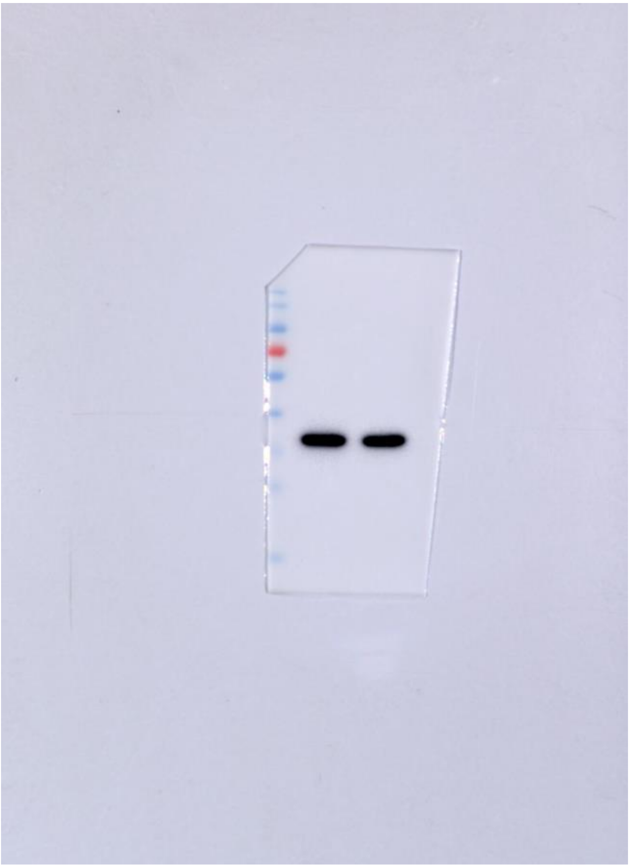

BT-549 CCNE1

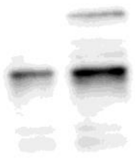

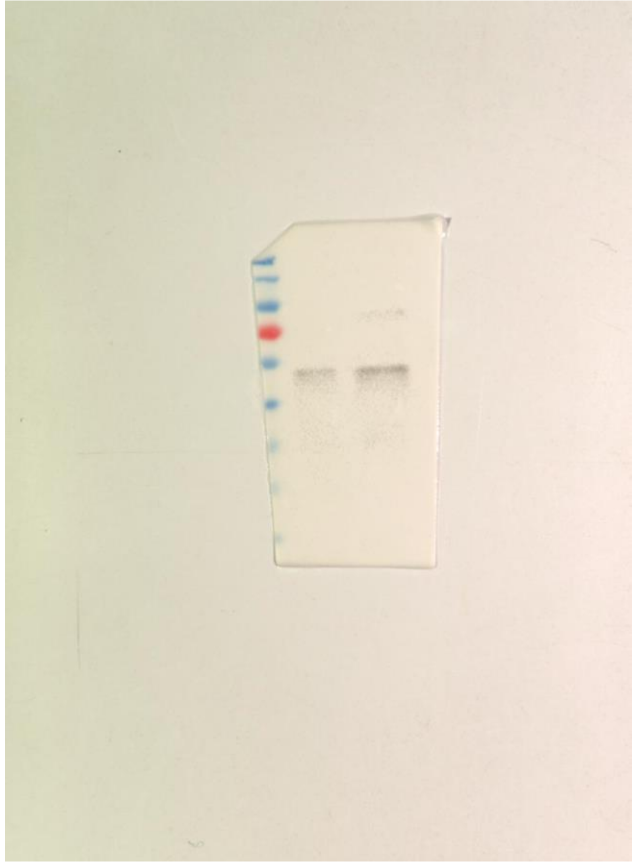

BT-549 GAPDH

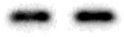

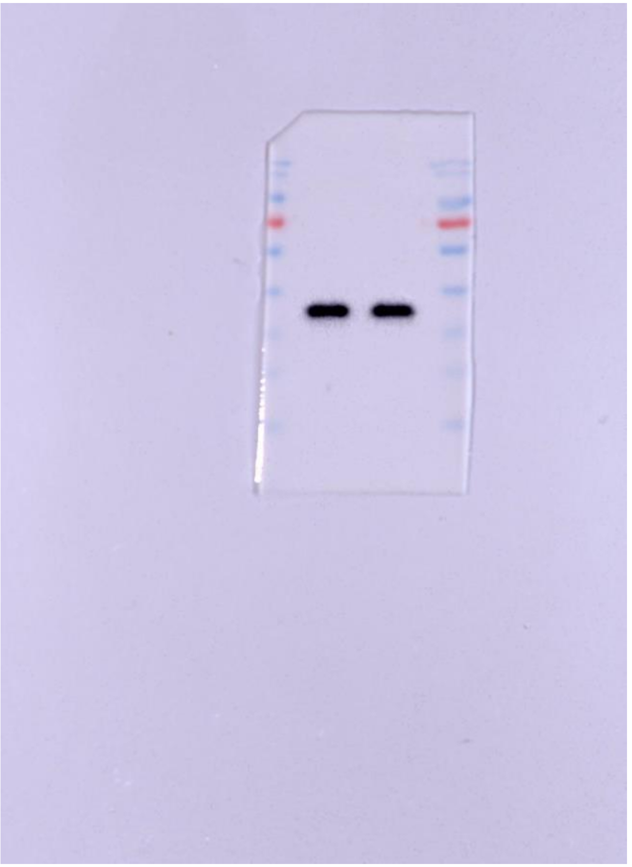

MDA-MB-231 CCNE1

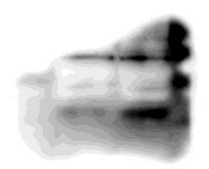

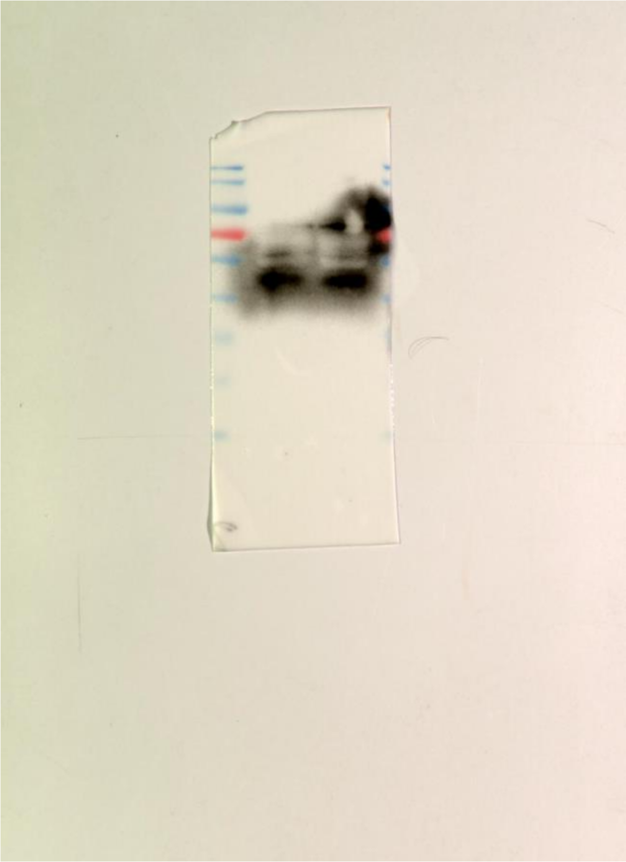

MDA-MB-231 GAPDH

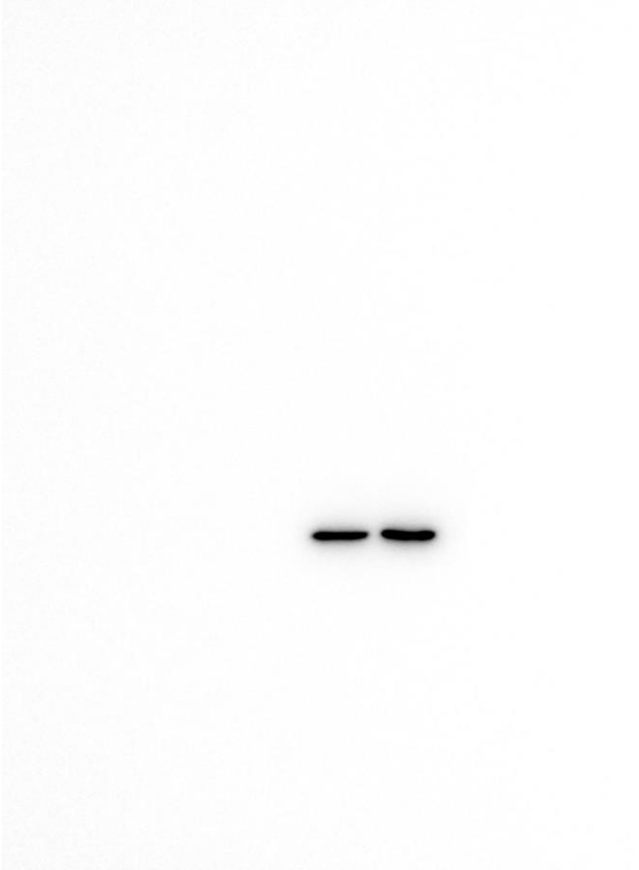

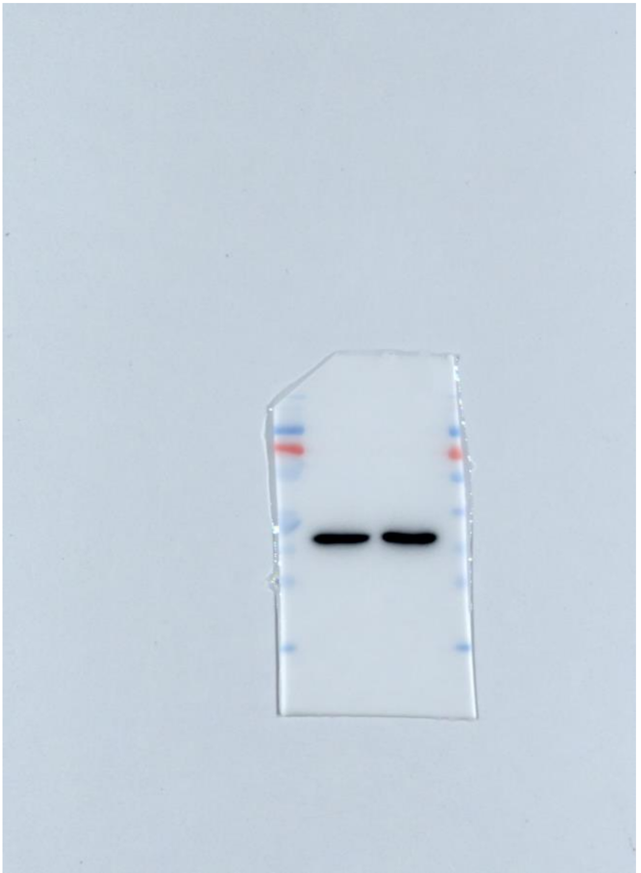

BT-549

MDA-MB-231

CCNE1

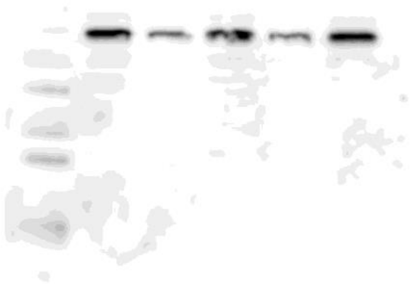

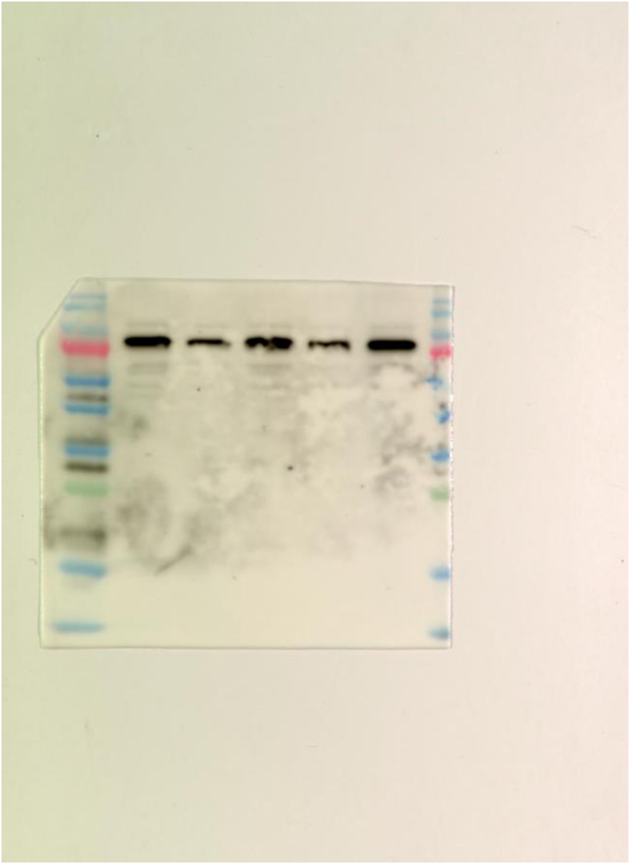

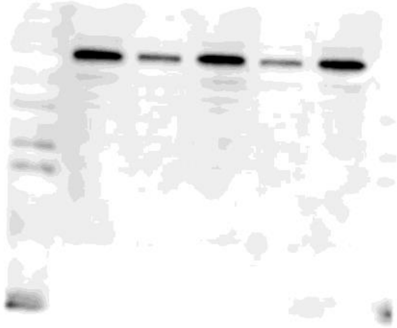

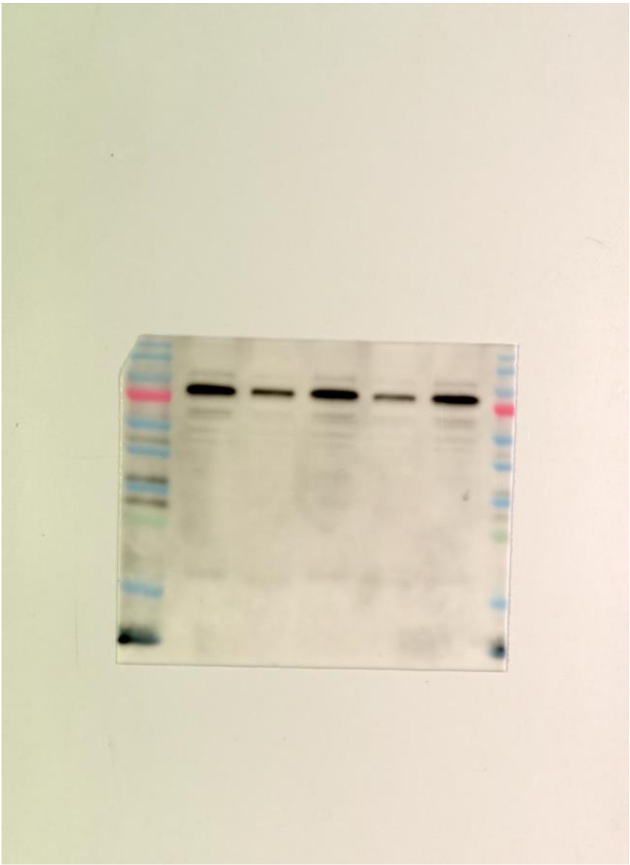

BT-549

MDA-MB-231

CCNE2

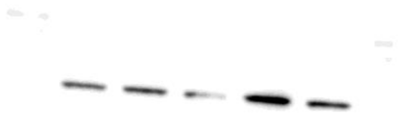

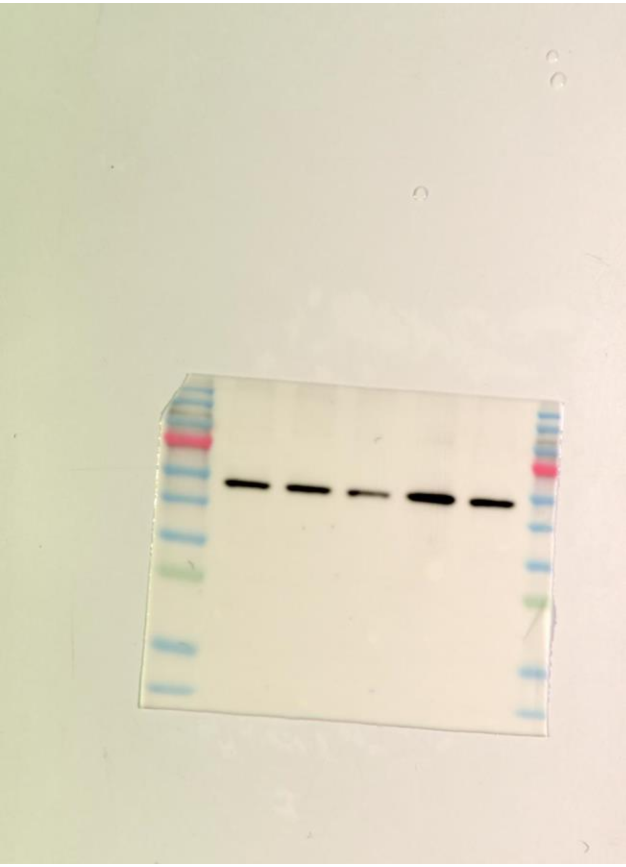

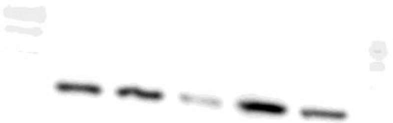

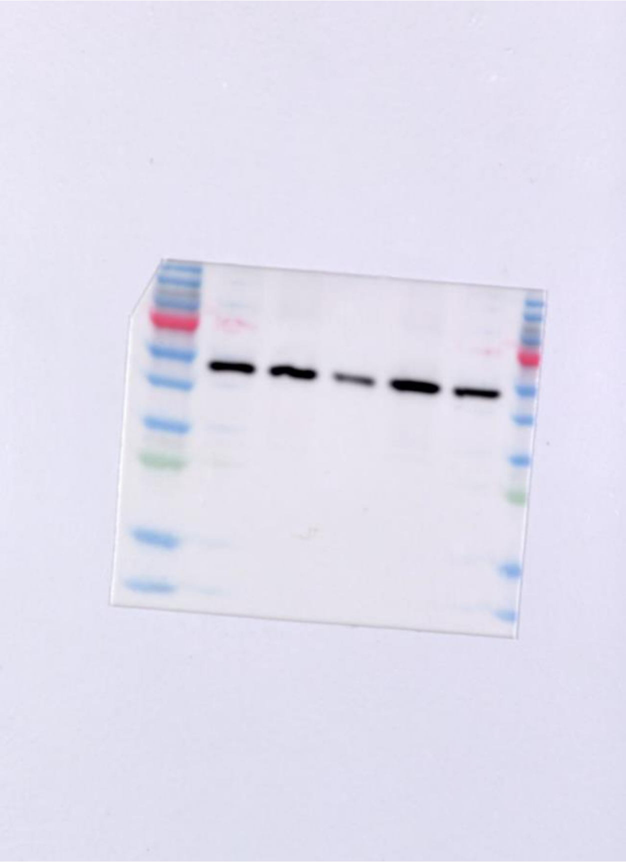

BT-549

MDA-MB-231

RB1

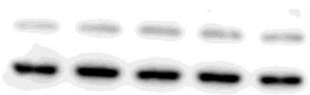

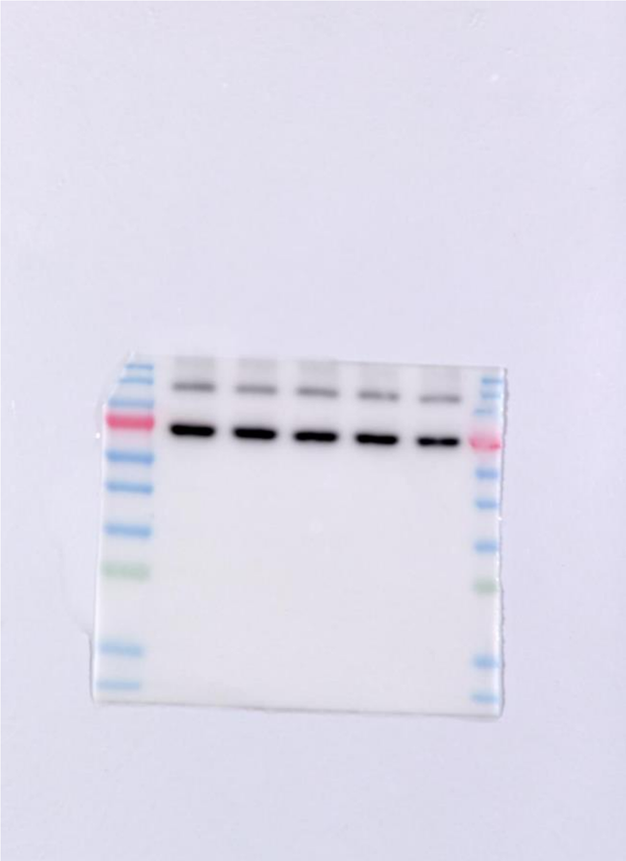

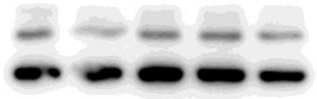

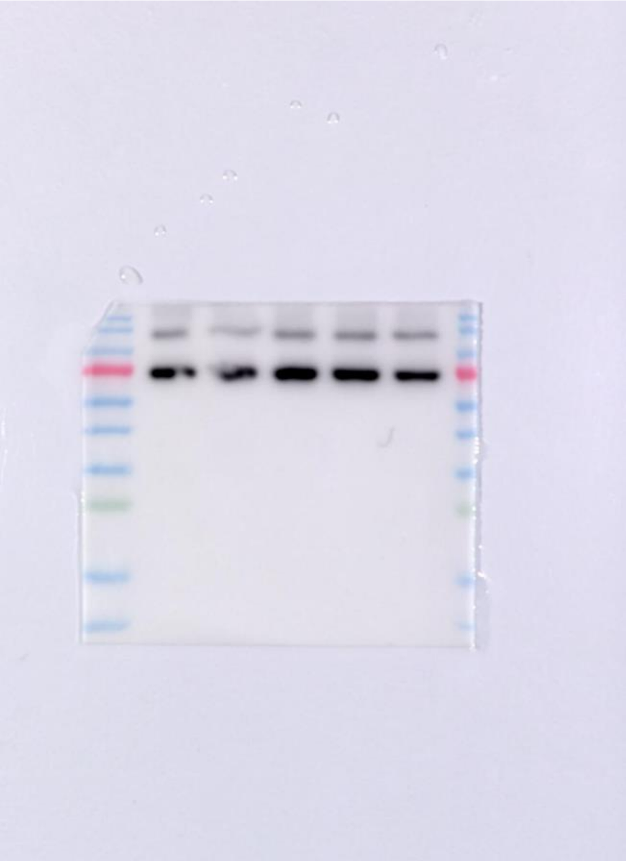

BT-549

MDA-MB-231

P-RB1

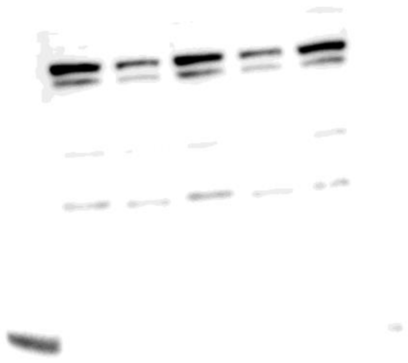

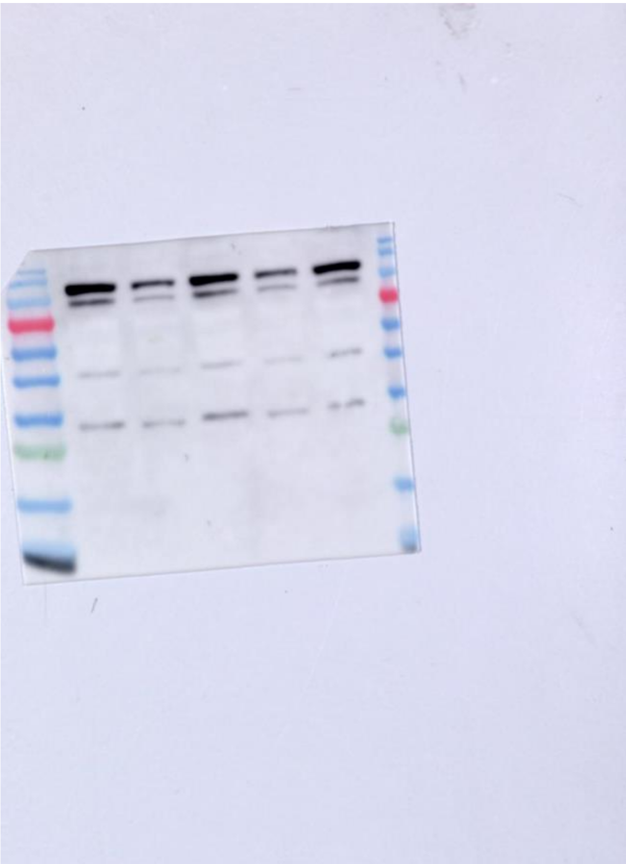

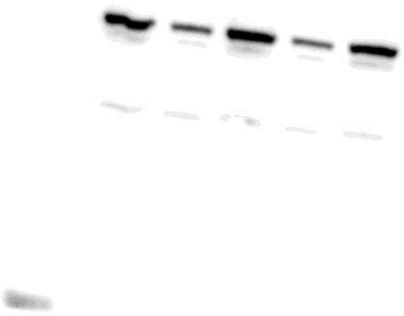

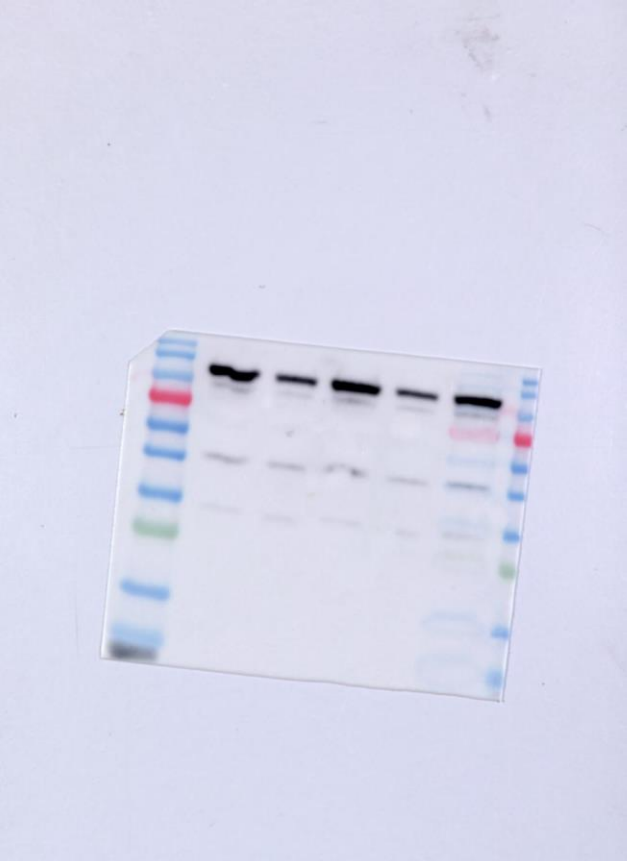

BT-549

MDA-MB-231

GAPDH

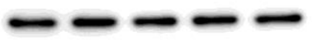

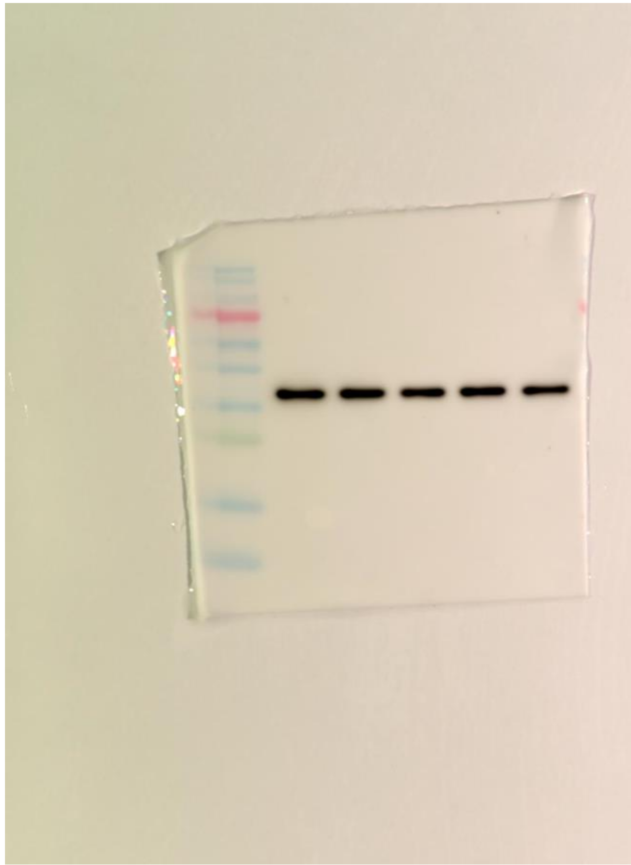

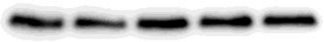

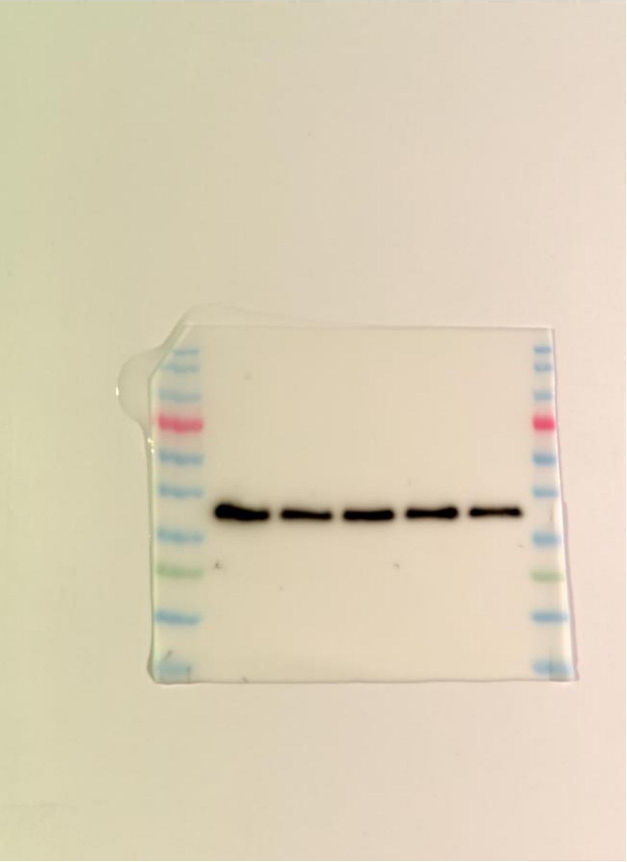

Supplement: Supplementary file 5 — WB inages [file 41420_2025_2518_MOESM5_ESM.pdf]
